# Model metamers reveal divergent invariances between biological and artificial neural networks

**DOI:** 10.1038/s41593-023-01442-0

**Published:** 2023-10-16

**Authors:** Jenelle Feather, Guillaume Leclerc, Aleksander Mądry, Josh H. McDermott

**Affiliations:** 1 Department of Brain and Cognitive Sciences, Massachusetts Institute of Technology, Cambridge, MA USA; 2 McGovern Institute, Massachusetts Institute of Technology, Cambridge, MA USA; 3 Center for Brains, Minds and Machines, Massachusetts Institute of Technology, Cambridge, MA USA; 4 Department of Electrical Engineering and Computer Science, Massachusetts Institute of Technology, Cambridge, MA USA; 5 Computer Science and Artificial Intelligence Laboratory, Massachusetts Institute of Technology, Cambridge, MA USA; 6 Speech and Hearing Bioscience and Technology, Harvard University, Cambridge, MA USA; 7 Present Address: Center for Computational Neuroscience, Flatiron Institute, Cambridge, MA USA

**Keywords:** Sensory processing, Object vision, Auditory system

## Abstract

Deep neural network models of sensory systems are often proposed to learn representational transformations with invariances like those in the brain. To reveal these invariances, we generated ‘model metamers’, stimuli whose activations within a model stage are matched to those of a natural stimulus. Metamers for state-of-the-art supervised and unsupervised neural network models of vision and audition were often completely unrecognizable to humans when generated from late model stages, suggesting differences between model and human invariances. Targeted model changes improved human recognizability of model metamers but did not eliminate the overall human–model discrepancy. The human recognizability of a model’s metamers was well predicted by their recognizability by other models, suggesting that models contain idiosyncratic invariances in addition to those required by the task. Metamer recognizability dissociated from both traditional brain-based benchmarks and adversarial vulnerability, revealing a distinct failure mode of existing sensory models and providing a complementary benchmark for model assessment.

## Abstract (web summary)

The authors test artificial neural networks with stimuli whose activations are matched to those of a natural stimulus. These ‘model metamers’ are often unrecognizable to humans, demonstrating a discrepancy between human and model sensory systems.

## Main

A central goal of neuroscience is to build models that reproduce brain responses and behavior. The hierarchical nature of biological sensory systems^1^ has motivated the use of hierarchical neural network models that transform sensory inputs into task-relevant representations^2,3^. As such models have become the top-performing machine perception systems over the last decade, they have also emerged as the leading models of both the visual and auditory systems^4,5^.

One hypothesis for why artificial neural network models might replicate computations found in biological sensory systems is that they instantiate invariances that mirror those in such systems^6,7^. For instance, visual object recognition must often be invariant to pose and to the direction of illumination. Similarly, speech recognition must be invariant to speaker identity and to details of the prosodic contour. Sensory systems are hypothesized to build up invariances^8,9^ that enable robust recognition. Such invariances plausibly arise in neural network models as a consequence of optimization for recognition tasks or other training objectives.

Although biological and artificial neural networks might be supposed to have similar internal invariances, there are some known human–model discrepancies that suggest that the invariances of the two systems do not perfectly match. For instance, model judgments are often impaired by stimulus manipulations to which human judgments are invariant, such as additive noise^10,11^ or small translations of the input^12,13^. Another such discrepancy is the vulnerability to adversarial perturbations (small changes to stimuli that alter model decisions despite being imperceptible to humans^14,15^). Although these findings illustrate that current task-optimized models lack some of the invariances of human perception, they leave many questions unresolved. For instance, because the established discrepancies rely on only the model’s output decisions, they do not reveal where in the model the discrepancies arise. It also remains unclear whether observed discrepancies are specific to supervised learning procedures that are known to deviate from biological learning. Finally, because we have lacked a general method to assess model invariances in the absence of a specific hypothesis, it remains possible that current models possess many other invariances that humans lack.

Here, we present a general test of whether the invariances present in computational models of the auditory and visual systems are also present in human perception. Rather than target particular known human invariances, we visualize or sonify model invariances by synthesizing stimuli that produce approximately the same activations in a model. We draw inspiration from human perceptual metamers (stimuli that are physically distinct but that are indistinguishable to human observers because they produce the same response at some stage of a sensory system), which have previously been characterized in the domains of color perception^16,17^, texture^18–20^, cue combination^21^, Bayesian decision-making^22^ and visual crowding^23,24^. We call the stimuli we generate ‘model metamers’ because they are metameric for a computational model^25^.

We generated model metamers from a variety of deep neural network models of vision and audition by synthesizing stimuli that yielded the same activations in a model stage as particular natural images or sounds. We then evaluated human recognition of the model metamers. If the model invariances match those of humans, humans should be able to recognize the model metamer as belonging to the same class as the natural signal to which it is matched.

Across both visual and auditory task-optimized neural networks, metamers from late model stages were nearly always misclassified by humans, suggesting that many of their invariances are not present in human sensory systems. The same phenomenon occurred for models trained with unsupervised learning, demonstrating that the model failure is not specific to supervised classifiers. Model metamers could be made more recognizable to humans with selective changes to the training procedure or architecture. However, late-stage model metamers remained much less recognizable than natural stimuli in every model we tested regardless of architecture or training. Some model changes that produced more recognizable metamers did not improve conventional neural prediction metrics or evaluations of robustness, demonstrating that the metamer test provides a complementary tool to guide model improvements. Notably, the human recognizability of a model’s metamers was well predicted by other models’ recognition of the same metamers, suggesting that the discrepancy with humans lies in idiosyncratic model-specific invariances. Model metamers demonstrate a qualitative gap between current models of sensory systems and their biological counterparts and provide a benchmark for future model evaluation.

## Results

### General procedure

**Fig. 1:**
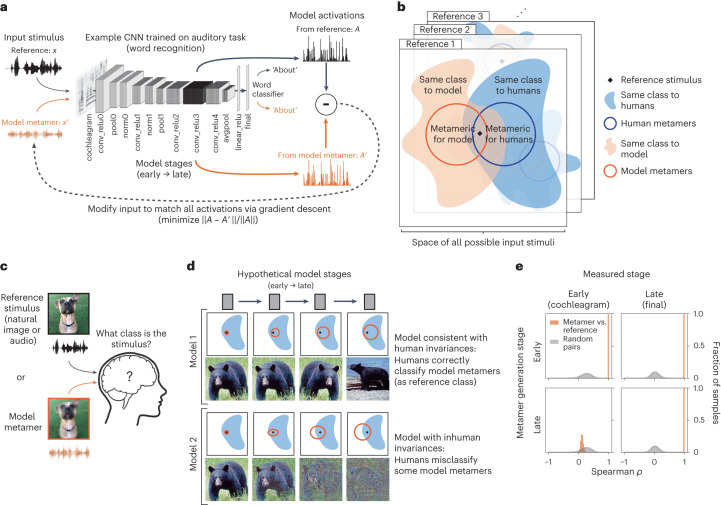
Overview of model metamers methodology. **a**, Model metamer generation. Metamers are synthesized by performing gradient descent on a noise signal to minimize the difference (normalized Euclidean distance) between its activations at a model stage and those of a natural signal. The architecture shown is the CochCNN9 auditory model. **b**, Each reference stimulus has an associated set of stimuli that are categorized as the same class by humans (blue) or by models (orange, if models have a classification decision). Metamers for humans and metamers for models are also sets of stimuli in the space of all possible stimuli (subsets of the set of same-class stimuli). Here, model metamers are derived for a specific model stage, taking advantage of access to the internal representations of the model at each stage. **c**, General experimental setup. Because we do not have high-resolution access to the internal brain representations of humans, we test for shared invariances behaviorally, asking humans to make classification judgments on natural stimuli or model metamers. See text for justification of the use of a classification task. **d**, Possible scenarios for how model metamers could relate to human classification decisions. Each square depicts sets of stimuli in the input space. Model 1 represents a model that passes our proposed behavioral test. The set of metamers for a reference stimulus grows over the course of the model, but even at the last stage, all model metamers are classified as the reference category by humans. Model 2 represents a model whose invariances diverge from those of humans. By the late stages of the model, many model metamers are no longer recognizable by humans as the reference stimulus class. The metamer test results thus reveal the model stage at which model invariances diverge from those of humans. **e**, Example distributions of activation similarity for pairs of metamers (a natural reference stimulus and its corresponding metamer) along with random pairs of natural stimuli from the training set. The latter provides a null distribution that we used to verify the success of the model metamer generation. Distributions were generated from the first and last stage of the CochCNN9 auditory model.

The goal of our metamer generation procedure (Fig. 1a) was to generate stimuli that produce nearly identical activations at some stage within a model but that were otherwise unconstrained and thus could differ in ways to which the model was invariant. We first measured the activations evoked by a natural image or sound at a particular model stage. The metamer for the natural image or sound was then initialized as a white noise signal (either an image or a sound waveform; white noise was chosen to sample the metamers as broadly as possible subject to the model constraints without biasing the initialization toward a specific object class). The noise signal was then modified to minimize the difference between its activations at the model stage of interest and those for the natural signal to which it was matched. The optimization procedure performed gradient descent on the input, iteratively updating the input while holding the model parameters fixed. Model metamers can be generated in this way for any model stage constructed from differentiable operations. Because the models that we considered are hierarchical, if the image or sound was matched with high fidelity at a particular stage, all subsequent stages were also matched (including the final classification stage in the case of supervised models, yielding the same decision).

### Experimental logic

The logic of our approach can be related to four sets of stimuli. For a given ‘reference’ stimulus, there is a set of stimuli for which humans produce the same classification judgment as the reference (Fig. 1b). A subset of these are stimuli that are indistinguishable from the reference stimulus (that is, metameric) to human observers. If a model performs a classification task, it will also have a set of stimuli judged to be the same category as the reference stimulus, and a subset of these stimuli will produce the same activations at a given model stage (model metamers). Even if the model does not perform classification, it could instantiate invariances that define sets of model metamers for the reference stimulus at each model stage.

In our experiments, we generate stimuli (sounds or images) that are metameric to a model and present these stimuli to humans performing a classification task (Fig. 1c). Because we have access to the internal representations of the model, we can generate metamers for each model stage (Fig. 1d). In many models there is limited invariance in the early stages (as is believed to be true of early stages of biological sensory systems^9^), with model metamers closely approximating the stimulus from which they are generated (Fig. 1d, left). But successive stages of a model may build up invariance, producing successively larger sets of model metamers. In a feedforward model, if two distinct inputs map onto the same representation at a given model stage, then any differences in the inputs cannot be recovered in subsequent stages, such that invariance cannot decrease from one stage to the next. If a model replicates a human sensory system, every model metamer from each stage should also be classified as the reference class by human observers (Fig. 1d, top). Such a result does not imply that all human invariances will be shared by the model, but it is a necessary condition for a model to replicate human invariances.

Discrepancies in human and model invariances could result in model metamers that are not recognizable by human observers (Fig. 1d, bottom). The model stage at which this occurs could provide insight into where any discrepancies with humans arise within the model.

Our approach differs from classical work on metamers^17^ in that we do not directly assess whether model metamers are also metamers for human observers (that is, indistinguishable). The reason for this is that a human judgment of whether two stimuli are the same or different could rely on any representations within their sensory system that distinguish the stimuli (rather than just those that are relevant to a particular behavior). By contrast, most current neural network models of sensory systems are trained to perform a single behavioral task. As a result, we do not expect metamers of such models to be fully indistinguishable to a human, and the classical metamer test is likely to be too sensitive for our purposes. Models might fail the classical test even if they capture human invariances for a particular task. But if a model succeeds in reproducing human invariances for a task, its metamers should produce the same human behavioral judgment on that task because they should be indistinguishable to the human representations that mediate the judgment. We thus use recognition judgments as the behavioral assay of whether model metamers reflect the same invariances that are instantiated in an associated human sensory system. We note that if humans cannot recognize a model metamer, they would also be able to discriminate it from the reference stimulus, and the model would also fail a traditional metamerism test.

We sought to answer several questions. First, we asked whether the learned invariances of commonly used neural network models are shared by human sensory systems. Second, we asked where any discrepancies with human perception arise within models. Third, we asked whether any discrepancies between model and human invariances would also be present in models obtained without supervised learning. Fourth, we explored whether model modifications intended to improve robustness would also make model metamers more recognizable to humans. Fifth, we asked whether metamer recognition identifies model discrepancies that are not evident using other methods of model assessment, such as brain predictions or adversarial vulnerability. Sixth, we asked whether metamers are shared across models.

### Metamer optimization

Because metamer generation relies on an iterative optimization procedure, it was important to measure optimization success. We considered the procedure to have succeeded only if it satisfied two conditions. First, measures of the match between the activations for the natural reference stimulus and its model metamer at the matched stage had to be much higher than would be expected by chance, as quantified with a null distribution (Fig. 1e) measured between randomly chosen pairs of examples from the training dataset. This criterion was adopted in part because it is equally applicable to models that do not perform a task. Metamers had to pass this criterion for each of three different measures of the match (Pearson and Spearman correlations and signal-to-noise ratio (SNR) expressed in decibels (dB); Methods). Second, for models that performed a classification task, the metamer had to result in the same classification decision by the model as the reference stimulus. In practice, we trained linear classifiers on top of all unsupervised models, such that we were also able to apply this second criterion for them (to be conservative).

Example distributions of the match fidelity (using Spearman’s *ρ* in this example) are shown in Fig. 1e. Activations of the matched model stage have a correlation close to 1, as intended, and are well outside the null distribution for random pairs of training examples. As expected, given the feedforward nature of the model, matching at an early stage produces matched activations in a late stage (Fig. 1e). But because the models we consider build up invariances over a series of feedforward stages, stages earlier than the matched stage need not have the same activations and in general these differ from those for the original stimulus to which the metamer was matched (Fig. 1e). The match fidelity of this example was typical, and optimization summaries for each analyzed model are included at https://github.com/jenellefeather/model_metamers_pytorch.

### Metamers of standard visual deep neural networks

**Fig. 2:**
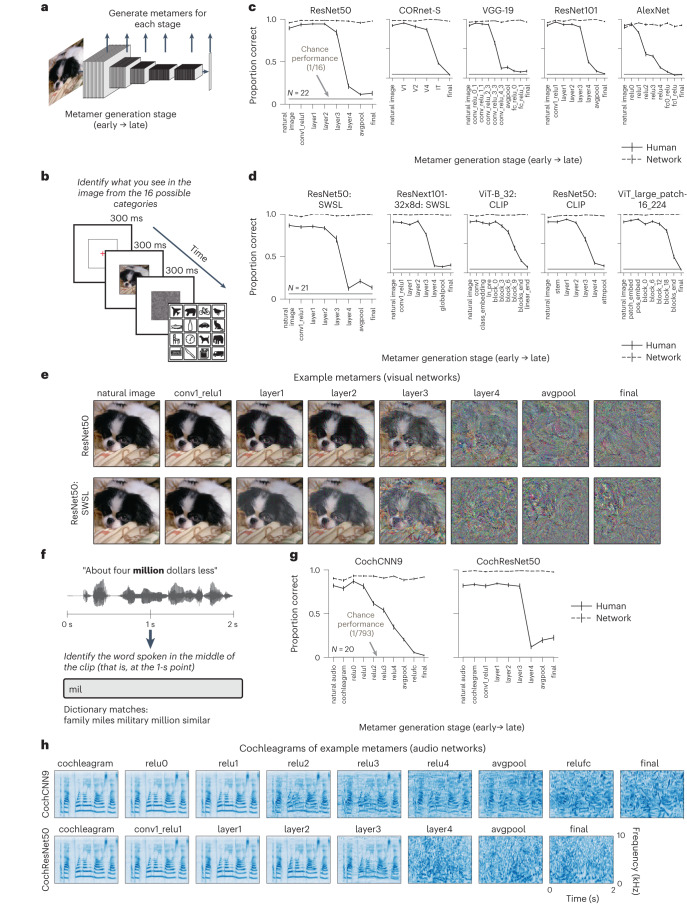
Metamers of standard-trained visual and auditory deep neural networks are often unrecognizable to human observers. **a**, Model metamers are generated from different stages of the model. Here and elsewhere, in models with residual connections, we only generated metamers from stages where all branches converge, which ensured that all subsequent model stages, and the model decision, remained matched. **b**, Experimental task used to assess human recognition of visual model metamers. Humans were presented with an image (a natural image or a model metamer of a natural image) followed by a noise mask. They were then presented with 16 icons representing 16 object categories and classified each image as belonging to one of the categories by clicking on the icon. **c**, Human recognition of visual model metamers (*N* = 22). At the time of the experiments the five models tested here placed 11th, 1st, 2nd, 4th and 59th (left to right) on a neural prediction benchmark^26,31^. For all tested models, human recognition of model metamers declined for late model stages, while model recognition remained high (as expected). Error bars plot s.e.m. across participants (or participant-matched stimulus subsets for model curves). **d**, Human recognition of visual model metamers (*N* = 21) trained on larger datasets. Error bars plot s.e.m. across participants (or participant-matched stimulus subsets for model curves). **e**, Example metamers from standard-trained and semi-weakly-supervised-learning (SWSL)-trained ResNet50 visual models. **f**, Experimental task used to assess human recognition of auditory model metamers. Humans classified the word that was present at the midpoint of a 2-s sound clip. Participants selected from 793 possible words by typing any part of the word into a response box and seeing matching dictionary entries from which to complete their response. A response could only be submitted if it matched an entry in the dictionary. **g**, Human recognition of auditory model metamers (*N* = 20). For both tested models, human recognition of model metamers decreased at late model stages, while model recognition remained high, as expected. When plotted, chance performance (1/793) is indistinguishable from the *x* axis. Error bars plot s.e.m. across participants (or participant-matched stimulus subsets for model curves). **h**, Cochleagram visualizations of example auditory model metamers from CochCNN9 and CochResNet50 architectures. Color intensity denotes instantaneous sound amplitude in a frequency channel (arbitrary units).

We generated metamers for multiple stages of five standard visual neural networks trained to recognize objects^26–29^ (trained on the ImageNet1K dataset^30^; Fig. 2a). The five models spanned a range of architectural building blocks and depths. Such models have been posited to capture similar features as primate visual representations, and, at the time the experiments were run, the five models placed 1st, 2nd, 4th, 11th and 59th on a neural prediction benchmark^26,31^. We subsequently ran a second experiment on an additional five models pretrained on larger datasets that became available at later stages of the project^32–34^. To evaluate human recognition of the model metamers, humans performed a 16-way categorization task on the natural stimuli and model metamers (Fig. 2b)^10^.

Contrary to the idea that the trained neural networks learned human-like invariances, human recognition of the model metamers decreased across model stages, reaching near-chance performance at the latest stages even though the model metamers remained as recognizable to the models as the corresponding natural stimuli, as intended (Fig. 2c,d). This reduction in human recognizability was evident as a main effect of observer and an interaction between the metamer generation stage and the observer, both of which were statistically significant for each of the ten models (*P* < 0.0001 in all cases; Supplementary Table 1).

**Extended Data Fig. 1:**
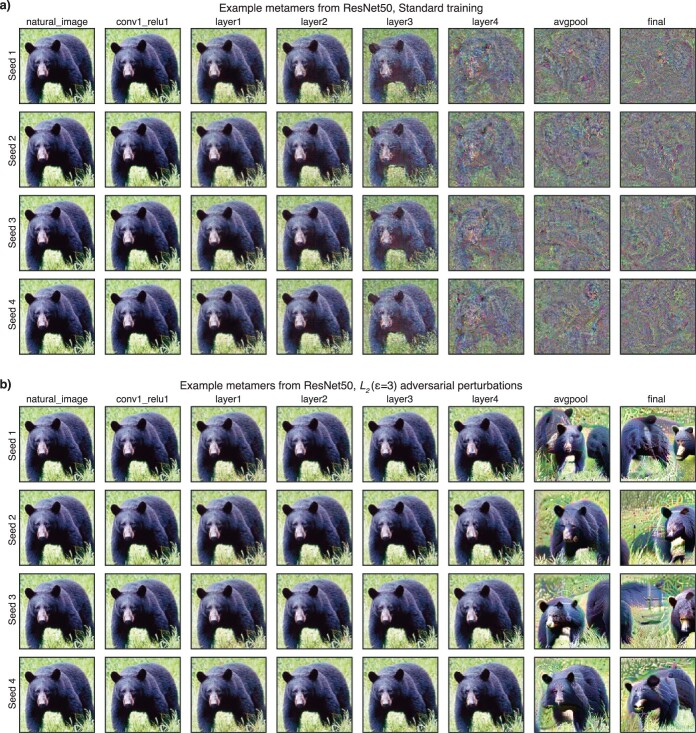
Model metamers generated from different noise initializations. **a**,**b**, Model metamers generated from four different white noise initializations for the Standard ResNet50 (**a**) and an adversarially trained ResNet50 (**b**).

**Extended Data Fig. 2:**
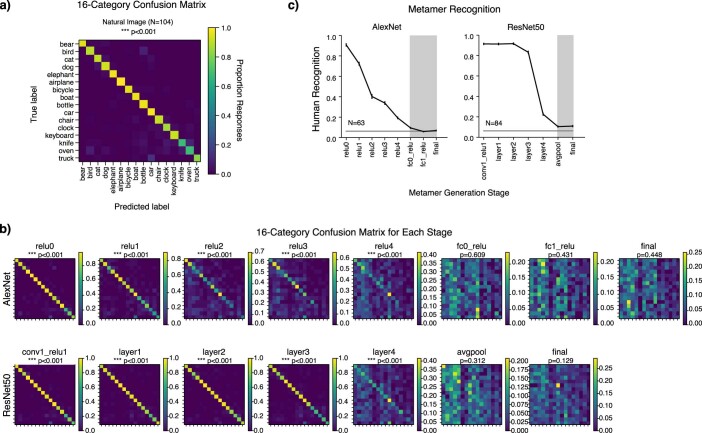
Analysis of consistency of human recognition errors for model metamers. **a**, 16-way confusion matrix for natural images. Here and in **b** and **c**, results incorporate human responses from all experiments that contained the AlexNet Standard architecture or the ResNet50 Standard architecture (N = 104 participants). Statistical test for confusion matrix described in **b**. **b**, Confusion matrices for human recognition judgments of model metamers from each stage of the AlexNet and ResNet50 models (using data from all experiments that contained the AlexNet Standard architecture or the ResNet50 Standard architecture). We performed a split-half reliability analysis of the confusion matrices to determine whether the confusions were reliable across participants. We measured the correlation between the confusion matrices for splits of human participants, and assessed whether this correlation was significantly greater than 0 (one-sided test). P-values from this analysis are given above each confusion matrix. For the later stages of each model, the confusion matrices are no more consistent than would be expected by chance, consistent with the metamers being completely unrecognizable (that is, containing no information about the visual category of the natural image they are matched to). **c**, Human recognizability of model metamers from different stages of AlexNet (N = 63 participants) and ResNet50 models (N = 84 participants). Error bars are s.e.m. across participants. Stages whose confusions were not consistent across splits of human observers are noted by the shaded region. The stages for which recognition is near chance show inconsistent confusion patterns, ruling out the possibility that the chance levels of recognition are driven by systematic errors (for example consistently recognizing metamers for cats as dogs).

**Extended Data Fig. 3:**
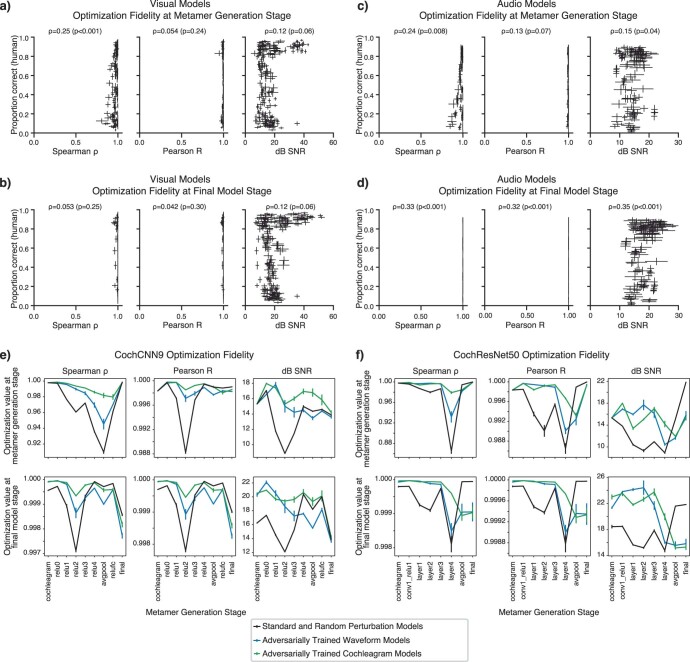
Optimization fidelity vs. human recognition of model metamers. **a**,**b**, Optimization fidelity for visual model metamers at the metamer generation stage (**a**) and at the final model stage corresponding to a categorization decision (**b**; N = 219 model stages). Visual models are those in Figs. 2–4 and Fig. 6g. Note that most data points are very close to 1 for the final stage correlation metrics (for example 209/219 stages exceed an average Spearman ρ of 0.99). Each point corresponds to a single stage of a single model. **c**,**d**, Optimization fidelity for auditory model metamers at the metamer generation stage (**c**) and at the final model stage corresponding to a categorization decision (**d**). Auditory models are those in Fig. 2 and Fig. 5 (N = 127 model stages). Note that most data points are again very close to 1 for the final stage correlation metrics (all 127 stages exceed an average Spearman ρ of 0.99). In all cases, optimization fidelity is high for both the metamer generation stage and the final stage, and human recognition is not predicted by the optimization fidelity, or only weakly correlated with the optimization fidelity for the generated model metamers (accounting for a very small fraction of the variance). Error bars on each data point are standard deviation across generated model metamers that passed the optimization criteria to be included in the psychophysical experiment. **e**,**f**, Final stage optimization fidelity plotted vs. model metamer generation stage for CochCNN9 auditory model (**e**) and CochResNet50 auditory model (**f**). Note the y axis limits, which differ across plots to show the small variations near 1 for the correlation measures. It is apparent that for any given model, some stages are somewhat less well optimized than others, but these variations do not account for the recognizability differences found in our experiments (compare these plots to the recognition plots in Figs. 2 and 5).

From visual inspection, many of the metamers from late stages resemble noise rather than natural images (Fig. 2e and see Extended Data Fig. 1a for metamers generated from different noise initializations). Moreover, analysis of confusion matrices revealed that for the late model stages, there was no detectably reliable structure in participant responses (Extended Data Fig. 2). Although the specific optimization strategies we used had some effect on the subjective appearance of model metamers, human recognition remained poor regardless of the optimization procedure (Supplementary Fig. 1). The poor recognizability of late-stage metamers was also not explained by less successful optimization; the activation matches achieved by the optimization were generally good (for example, with correlations close to 1), and what variation we did observe was not predictive of metamer recognizability (Extended Data Fig. 3).

### Metamers of standard auditory deep neural networks

We performed an analogous experiment with two auditory neural networks trained to recognize speech (the word recognition task in the Word–Speaker–Noise dataset^25^). Each model consisted of a biologically inspired ‘cochleagram’ representation^35,36^, followed by a convolutional neural network (CNN) whose parameters were optimized during training. We tested two model architectures: a ResNet50 architecture (henceforth referred to as CochResNet50) and a convolutional model with nine stages similar to that used in a previous publication^4^ (henceforth referred to as CochCNN9). Model metamers were generated for clean speech examples from the validation set. Humans performed a 793-way classification task^4^ to identify the word in the middle of the stimulus (Fig. 2f).

As with the visual models, human recognition of auditory model metamers decreased markedly at late model stages for both architectures (Fig. 2g), yielding a significant main effect of human versus model observer and an interaction between the model stage and the observer (*P* < 0.0001 for each comparison; Supplementary Table 1). Subjectively, the model metamers from later stages sound like noise (and appear noise-like when visualized as cochleagrams; Fig. 2h). This result suggests that many of the invariances present in these models are not invariances for the human auditory system.

Overall, these results demonstrate that the invariances of many common visual and auditory neural networks are substantially misaligned with those of human perception, even though these models are currently the best predictors of brain responses in each modality.

### Unsupervised models also exhibit discrepant metamers

Biological systems typically do not have access to labels at the scale that is needed for supervised learning^37^ and instead must rely in large part on unsupervised learning. Do the divergent invariances evident in neural network models result in some way from supervised training with explicit category labels? Metamers are well suited to address this question given that their generation is not dependent on a classifier and thus can be generated for any sensory model.

**Fig. 3:**
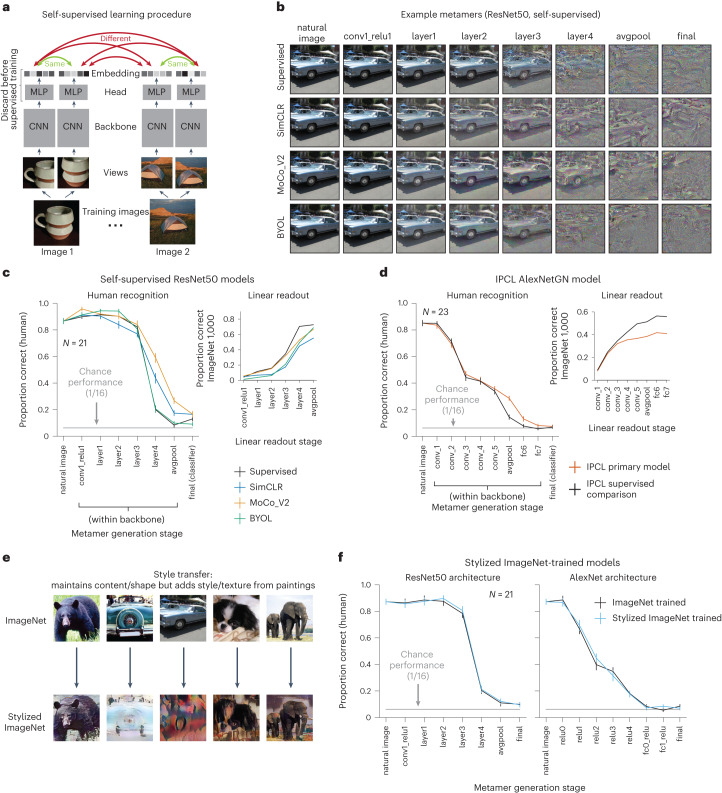
Model metamers are unrecognizable to humans even with alternative training procedures. **a**, Overview of self-supervised learning, inspired by Chen et al.^38^. Each input was passed through a learnable convolutional neural network (CNN) backbone and a multi-layer perceptron (MLP) to generate an embedding vector. Models were trained to map multiple views of the same image to nearby points in the embedding space. Three of the self-supervised models (SimCLR, MoCo_V2 and BYOL) used a ResNet50 backbone. The other self-supervised model (IPCL) had an AlexNet architecture modified to use group normalization. In both cases, we tested comparison supervised models with the same architecture. The SimCLR, MoCo_V2 and IPCL models also had an additional training objective that explicitly pushed apart embeddings from different images. **b**, Example metamers from select stages of ResNet50 supervised and self-supervised models. In all models, late-stage metamers were mostly unrecognizable. **c**, Human recognition of metamers from supervised and self-supervised models (left; *N* = 21) along with classification performance of a linear readout trained on the ImageNet1K task at each stage of the models (right). Readout classifiers were trained without changing any of the model weights. For self-supervised models, model metamers from the ‘final’ stage were generated from a linear classifier at the avgpool stage. Model recognition curves of model metamers were close to ceiling, as in Fig. 2, and are omitted here and in later figures for brevity. Here and in **d**, error bars plot s.e.m. across participants (left) or across three random seeds of model evaluations (right). **d**, Same as **c** but for the IPCL self-supervised model and supervised comparison with the same dataset augmentations (*N* = 23). **e**, Examples of natural and stylized images using the Stylized ImageNet augmentation. Training models on Stylized ImageNet was previously shown to reduce a model’s dependence on texture cues for classification^43^. **f**, Human recognition of model metamers for ResNet50 and AlexNet architectures trained with Stylized ImageNet (*N* = 21). Removing the texture bias of models by training on Stylized ImageNet does not result in more recognizable model metamers than the standard model. Error bars plot s.e.m. across participants.

At present, the leading unsupervised models are ‘self-supervised’, being trained with a loss function favoring representations in which variants of a single training example (different crops of an image, for instance) are similar, whereas those from different training examples are not^38^ (Fig. 3a). We generated model metamers for four such models^38–41^ along with supervised comparison models with the same architectures.

**Extended Data Fig. 4:**
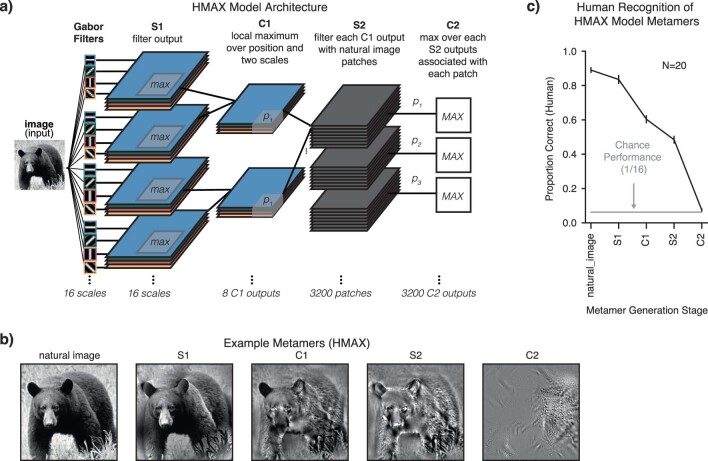
Metamers from a classic vision model. **a**, Schematic of HMAX vision model. The HMAX vision model is a biologically-motivated architecture with cascaded filtering and pooling operations inspired by simple and complex cells in the primate visual system and was intended to capture aspects of biological object recognition^3,8^. We generated model metamers by matching all units at the S1, C1, S2, or C2 stage of the model. **b**, Example HMAX model metamers. **c**, Although HMAX is substantially shallower than the “deep” neural network models investigated in the rest of this paper, it is evident that by the C2 model stage its model metamers are comparably unrecognizable to humans (significant main effect of model stage, F(4,76) = 351.9, p < 0.0001, ${\eta }_{p}^{2}=0.95$). This classical model thus also has invariances that differ from those of the human object recognition system. Error bars plot s.e.m. across participants (N = 20). HMAX metamers were black and white, while all metamers from all other models were in color.

**Extended Data Fig. 5:**
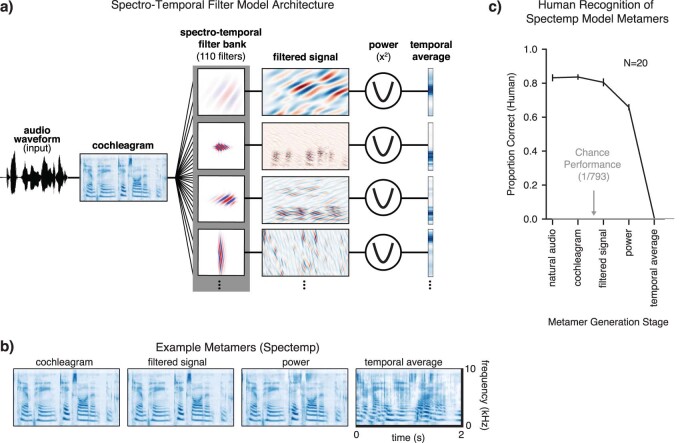
Metamers from a classic auditory model. **a**, Schematic of spectro-temporal auditory filterbank model (Spectemp), a classical model of auditory cortex consisting of a set of spectrotemporal filters applied to a cochleagram representation^42^. We used a version of the model in which the convolutional responses are summarized with the mean power in each filter^4,103,104^. **b**, Cochleagrams of example Spectemp model metamers. **c**, Human recognition of Spectemp model metamers. Metamers from the first two stages were fully recognizable, and subjectively resembled the original audio, indicating that these stages instantiate few invariances, as expected for overcomplete filter-bank decompositions. By contrast, metamers from the temporal average representation were unrecognizable (significant main effect of model stage F(4,76) = 515.3, p < 0.0001, ${\eta }_{p}^{2}=0.96$), indicating that this model stage produces invariances that humans do not share (plausibly because the human speech recognition system retains information that is lost in the averaging stage). Error bars plot s.e.m. across participants (N = 20). Overall, these results and those in Extended Data Fig. 4 show how metamers can reveal the invariances present in classical models as well as state-of-the-art deep neural networks, and demonstrate that both types of models fail to fully capture the invariances of biological sensory systems.

As shown in Fig. 3b–d, the self-supervised models produced similar results as those for supervised models. Human recognition of model metamers declined at late model stages, approaching chance levels for the final stages. Some of the models had more recognizable metamers at intermediate stages (significant interaction between model type and model stage; ResNet50 models: *F*_21,420_ = 16.0, *P* < 0.0001, ${\eta }_{p}^{2}=0.44$; IPCL model: *F*_9,198_ = 3.13, *P* = 0.0018, ${\eta }_{p}^{2}=0.12$). However, for both architectures, recognition was low in absolute terms, with the metamers bearing little resemblance to the original image they were matched to. Overall, the results suggest that the failure of standard neural network models to pass our metamer test is not specific to the supervised training procedure. This result also demonstrates the generality of the metamers method, as it can be applied to models that do not have a behavioral readout. Analogous results with two classical sensory system models (HMAX^3,8^ and a spectrotemporal modulation filterbank^42^), which further illustrate the general applicability of the method, are shown in Extended Data Figs. 4 and 5.

### Discrepant metamers are not explained by texture bias

Another commonly noted discrepancy between current models and humans is the tendency for models to base their judgments on texture rather than shape^43–45^. This ‘texture bias’ can be reduced with training datasets of ‘stylized’ images (Fig. 3e) that increase a model’s reliance on shape cues, making them more human-like in this respect^43^. To assess whether these changes also serve to make model metamers less discrepant, we generated metamers from two models trained on Stylized ImageNet. As shown in Fig. 3f, these models had metamers that were comparably unrecognizable to humans as those from models trained on the standard ImageNet1K training set (no interaction between model type and model stage; ResNet50: *F*_7,140_ = 0.225, *P* = 0.979, ${\eta }_{p}^{2}=0.011$; AlexNet: *F*_8,160_ = 0.949, *P* = 0.487, ${\eta }_{p}^{2}=0.045$). This result suggests that metamer discrepancies are not simply due to texture bias in the models.

### Effects of adversarial training on visual model metamers

**Fig. 4:**
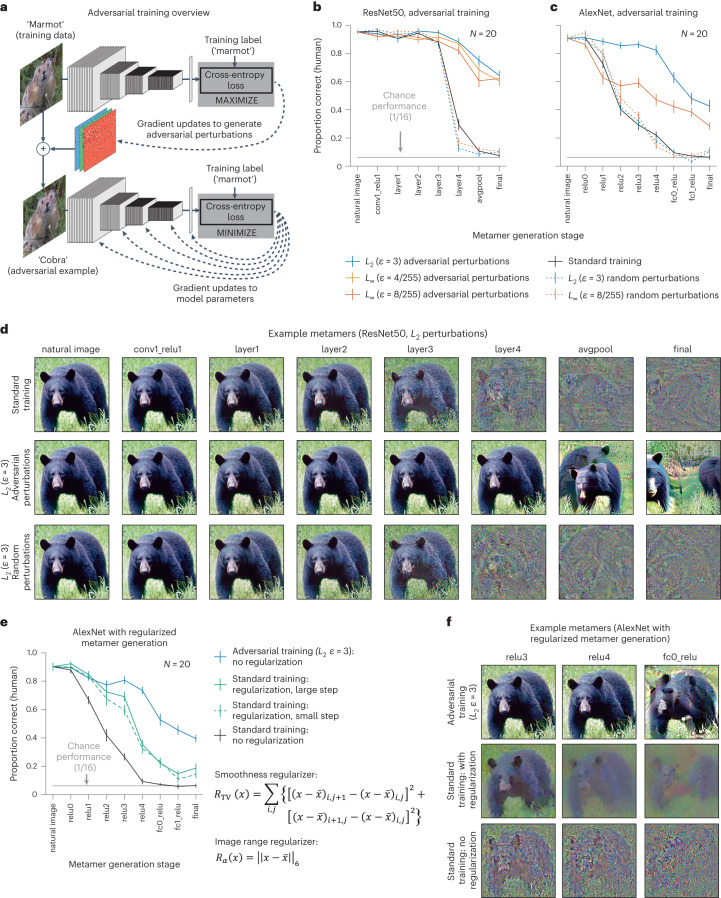
Adversarial training increases human recognizability of visual model metamers. **a**, Adversarial examples are derived at each training step by finding an additive perturbation to the input that moves the classification label away from the training label class (top). These derived adversarial examples are provided to the model as training examples and used to update the model parameters (bottom). The resulting model is subsequently more robust to adversarial perturbations than if standard training was used. As a control experiment, we also trained models with random perturbations to the input. **b**, Human recognition of metamers from ResNet50 models (*N* = 20) trained with and without adversarial or random perturbations. Here and in **c** and **e**, error bars plot s.e.m. across participants. **c**, Same as **b** but for AlexNet models (*N* = 20). In both architectures, adversarial training led to more recognizable metamers at deep model stages (repeated measures analysis of variance (ANOVA) tests comparing human recognition of standard and each adversarial model; significant main effects in each case, *F*_1,19_ > 104.61, *P* < 0.0001, ${\eta }_{p}^{2} > 0.85$), although in both cases, the metamers remain less than fully recognizable. Random perturbations did not produce the same effect (repeated measures ANOVAs comparing random to adversarial; significant main effect of random versus adversarial for each perturbation of the same type and size, *F*_1,19_ > 121.38, *P* < 0.0001, ${\eta }_{p}^{2} > 0.86$). **d**, Example visual metamers for models trained with and without adversarial or random perturbations. **e**, Recognizability of model metamers from standard-trained models with and without regularization compared to that for an adversarially trained model (*N* = 20). Two regularization terms were included in the optimization: a total variation regularizer to promote smoothness and a constraint to stay within the image range^51^. Two optimization step sizes were evaluated. Smoothness priors increased recognizability for the standard model (repeated measures ANOVAs comparing human recognition of metamers with and without regularization; significant main effects for each step size, *F*_1,19_ > 131.8246, *P* < 0.0001, ${\eta }_{p}^{2} > 0.87$). However, regularized metamers remained less recognizable than those from the adversarially trained model (repeated measures ANOVAs comparing standard model metamers with regularization to metamers from adversarially trained models; significant main effects for each step size, *F*_1,19_ > 80.8186, *P* < 0.0001, ${\eta }_{p}^{2} > 0.81$). **f**, Example metamers for adversarially trained and standard models with and without regularization.

A known peculiarity of contemporary artificial neural networks is their vulnerability to small adversarial perturbations designed to change the class label predicted by a model^14,15^. Such perturbations are typically imperceptible to humans due to their small magnitude but can drastically alter model decisions and have been the subject of intense interest in part due to the security risk they pose for machine systems. One way to reduce this vulnerability is via ‘adversarial training’ in which adversarial perturbations are generated during training, and the model is forced to learn to recognize the perturbed images as the ‘correct’ human-interpretable class^46^ (Fig. 4a). This adversarial training procedure yields models that are less susceptible to adversarial examples for reasons that remain debated^47^.

We asked whether adversarial training would improve human recognition of model metamers. A priori, it was not clear what to expect. Making models robust to adversarial perturbations causes them to exhibit more of the invariances of humans (the shaded orange covers more of the blue outline in Fig. 1b), but it is not obvious that this will reduce the model invariances that are not shared by humans (that is, to decrease the orange outlined regions that do not overlap with the blue shaded region in Fig. 1b). Previous work visualizing latent representations of visual neural networks suggested that robust training might make model representations more human-like^48^, but human recognition of model metamers had not been behaviorally evaluated.

We first generated model metamers for five adversarially trained vision models^48^ with different architectures and perturbation sizes. As a control, we also trained models with equal magnitude perturbations in random, rather than adversarial, directions, which are typically ineffective at preventing adversarial attacks^49^. As intended, adversarially trained models were more robust to adversarial perturbations than the standard-trained model or models trained with random perturbations (Supplementary Fig. 2a,b).

**Extended Data Fig. 6:**
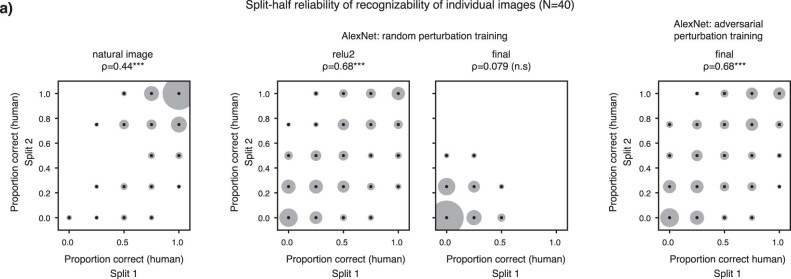
Consistency of human recognition of individual metamers from models trained with random or adversarial perturbations. **a**, Consistency of recognizability of individual stimuli across splits of participants. Graph plots the proportion correct for individual stimuli for one random split. Circle size represents the number of stimuli at that particular value. Correlation values were determined by averaging the Spearman ρ over 1000 random splits of participants (p-values were computed non-parametrically by shuffling participant responses for each condition, and computing the number of times the true Spearman ρ averaged across splits was lower than the shuffled correlation value; one-sided test). We only included images that had at least 4 trials in each split of participants, and we only included 4 trials in the average, to avoid having some images exert more influence on the result than others (resulting in quantized values for proportion correct). Recognizability of individual stimuli was reliable for natural images, relu2 of AlexNet trained with random perturbation training and the final stage of adversarially trained AlexNet (p < 0.001 in each case). By contrast, recognizability of individual metamers from the final stage of AlexNet trained with random perturbations showed no consistency across participants (p = 0.485).

**Extended Data Fig. 7:**
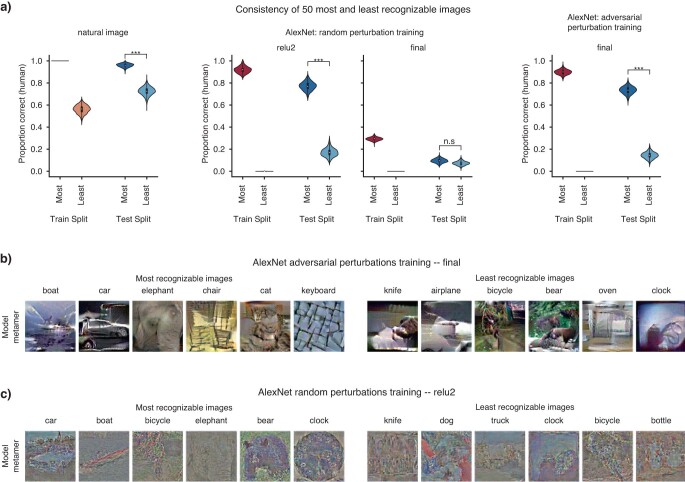
Analysis of most and least recognizable visual model metamers. **a**, Using half of the N = 40 participants, we selected the 50 images with the highest recognizability and the 50 images with the lowest recognizability (“train” split). We then measured the recognizability for these most and least recognizable images in the other half of participants (“test” split). We analyzed 1000 random participant splits; p-values were computed by measuring the number of times the “most” recognizable images had higher recognizability than the “least” recognizable images (one-sided test). Graph shows violin plots of test split results for the 1000 splits. Images were only included in analysis if they had responses from at least 4 participants in each split. The difference between the most and least recognizable metamers replicated across splits for the model stages with above-chance recognizability (p < 0.001), indicating that human observers agree on which metamers are recognizable (but not for the final stage of AlexNet trained with random perturbations, p = 0.165). Box plots within violins are defined with a center dot as the median value, bounds of box as the 25^th^-75^th^ percentile, and the whiskers as the 1.5x interquartile range. **b**,**c**, Example model metamers from the 50 “most” and “least” recognizable metamers for the final stage of adversarially trained AlexNet (**b**) and for the relu2 stage of AlexNet trained with random perturbations (evaluated with data from all participants; **c**). All images shown had at least 8 responses across participants for both the natural image and model metamer condition, had 100% correct responses for the natural image condition, and had 100% correct (for “most” recognizable images) or 0% correct (for “least” recognizable images).

Metamers for the adversarially trained models were in all cases significantly more recognizable than those from the standard model (Fig. 4b–d and Extended Data Fig. 1), evident as a main effect of training type in each case. Training with random perturbations did not yield the same benefit (Supplementary Table 2). Despite some differences across adversarial training variants, all variants that we tried produced a human recognition benefit. It was nonetheless the case that metamers for late stages remained less than fully recognizable to humans for all model variants. We note that performance is inflated by the use of a 16-way alternative force choice task, for which above-chance performance is possible even with severely distorted images. See Extended Data Figs. 6 and 7 for an analysis of the consistency of metamer recognition across human observers and examples of the most and least recognizable metamers.

**Extended Data Fig. 8:**
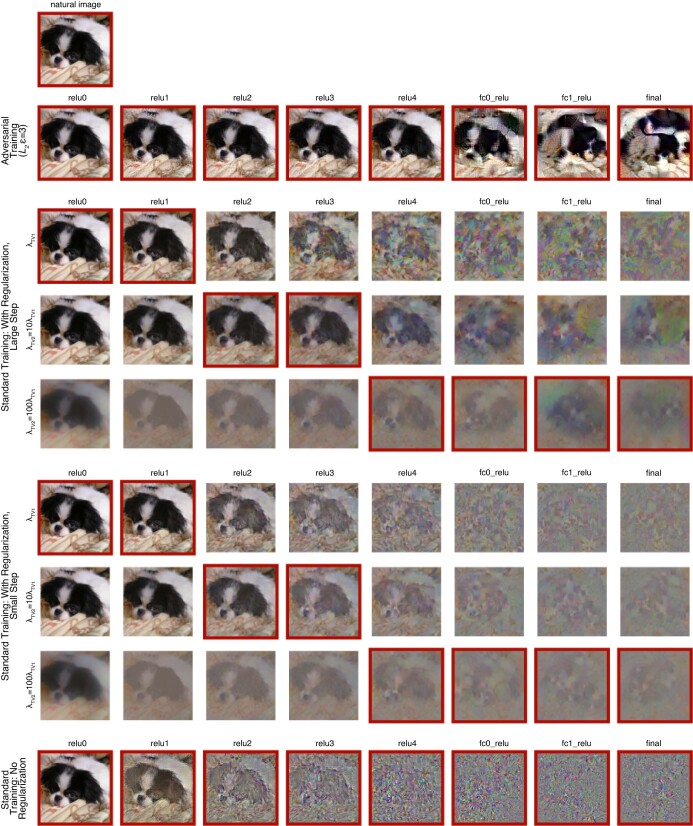
Examples of model metamers generated with regularization. Metamers were generated with terms for smoothness and image range included in the loss function. Three coefficients for the smoothness regularizer were used. Red outlines are present on conditions that were used in the human classification experiment (chosen to maximize the recognizability, and to match the choices in the original paper that introduced this type of regularization^51^).

Given that metamers from adversarially trained models look less noise-like than those from standard models and that standard models may overrepresent high spatial frequencies^50^, we wondered whether the improvement in recognizability could be replicated in a standard-trained model by including a smoothness regularizer in metamer optimization. Such regularizers are common in neural network visualizations^51^, and although they side step the goal of human–model comparison, it was nonetheless of interest to assess their effect. We implemented the regularizer used in a well-known visualization paper^51^. Adding smoothness regularization to the metamer generation procedure for the standard-trained AlexNet model improved the recognizability of its metamers (Fig. 4e) but not as much as did adversarial training (and did not come close to generating metamers as recognizable as natural images; see Extended Data Fig. 8 for examples generated with different regularization coefficients). This result suggests that the benefit of adversarial training is not simply replicated by imposing smoothness constraints and that discrepant metamers more generally cannot be resolved with the addition of a smoothness prior.

### Effects of adversarial training on auditory model metamers

We conducted analogous experiments with auditory models, again using two architectures and several perturbation types. Because the auditory models contain a fixed cochlear stage at their front end, there are two natural places to generate adversarial examples: they can be added to the waveform or the cochleagram. We explored both for completeness and found that adversarial training at either location resulted in adversarial robustness (Supplementary Fig. 2c–f).

**Fig. 5:**
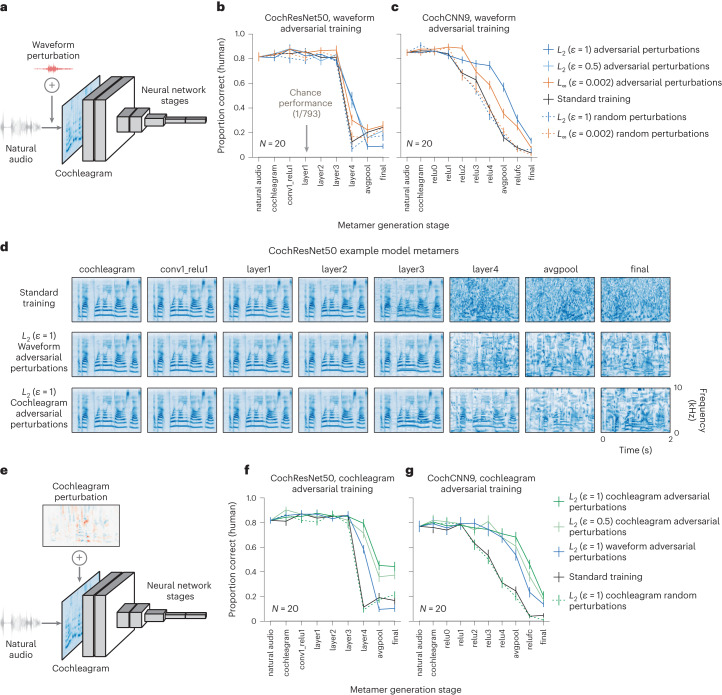
Adversarial training increases human recognition of auditory model metamers. **a**, Schematic of auditory CNNs with adversarial perturbations applied to the waveform input. **b**,**c**, Human recognition of auditory model metamers from CochResNet50 (**b**; *N* = 20) and CochCNN9 (**c**; *N* = 20) models with adversarial perturbations generated in the waveform space (models trained with random perturbations are also included for comparison). When plotted here and in **f** and **g**, chance performance (1/793) is indistinguishable from the *x* axis, and error bars plot s.e.m. across participants. *L*_2_ (*ε* = 0.5) waveform adversaries were only included in the CochResNet50 experiment. ANOVAs comparing standard and each adversarial model showed significant main effects in four of five cases (*F*_1,19_ > 9.26, *P* < 0.0075, ${\eta }_{p}^{2} > 0.33$) and no significant main effect for CochResNet50 with *L*_2_ (*ε* = 1) perturbations (*F*_1,19_ = 0.29, *P* = 0.59, ${\eta }_{p}^{2}=0.015$). Models trained with random perturbations did not show the same benefit (ANOVAs comparing each random and adversarial perturbation model with the same *ε* type and size; significant main effect in each case (*F*_1,19_ > 4.76, *P* < 0.0444, ${\eta }_{p}^{2} > 0.20$)). **d**, Cochleagrams of example model metamers from CochResNet50 models trained with waveform and cochleagram adversarial perturbations. **e**, Schematic of auditory CNNs with adversarial perturbations applied to the cochleagram stage. **f**,**g**, Human recognition of auditory model metamers from models trained with cochleagram adversarial perturbations are more recognizable for CochResNet50 (**f**) and CochCNN9 (**g**) models than those from models trained with waveform perturbations. ANOVAs comparing each model trained with cochleagram perturbations versus the same architecture trained with waveform perturbations showed significant main effects in each case (*F*_1,19_ > 4.6, *P* < 0.04, ${\eta }_{p}^{2} > 0.19$). ANOVAs comparing each model trained with cochleagram perturbations to the standard model showed a significant main effect in each case (*F*_1,19_ > 102.25, *P* < 0.0001, ${\eta }_{p}^{2} > 0.84$). The effect on metamer recognition was again specific to adversarial perturbations (ANOVAs comparing effect of training with adversarial versus random perturbations with the same *ε* type and size; *F*_1,19_ > 145.07, *P* < 0.0001, ${\eta }_{p}^{2} > 0.88$).

We first investigated adversarial training with perturbations to the waveform (Fig. 5a). As with the visual models, human recognition was generally better for metamers from adversarially trained models but not for models trained with random perturbations (Fig. 5b,c and Supplementary Table 2). The model metamers from the robust models were visibly less noise-like when viewed in the cochleagram representation (Fig. 5d).

We also trained models with adversarial perturbations to the cochleagram representation (Fig. 5e). These models had significantly more recognizable metamers than both the standard models and the models adversarially trained on waveform perturbations (Fig. 5f,g and Supplementary Table 2), and the benefit was again specific to models trained with adversarial (rather than random) perturbations. These results suggest that the improvements from intermediate-stage perturbations may in some cases be more substantial than those from perturbations to the input representation.

Overall, these results suggest that adversarial training can cause model invariances to become more human-like in both visual and auditory domains. However, substantial discrepancies remain, as many model metamers from late model stages remain unrecognizable even after adversarial training.

### Metamer recognizability dissociates from adversarial robustness

**Fig. 6:**
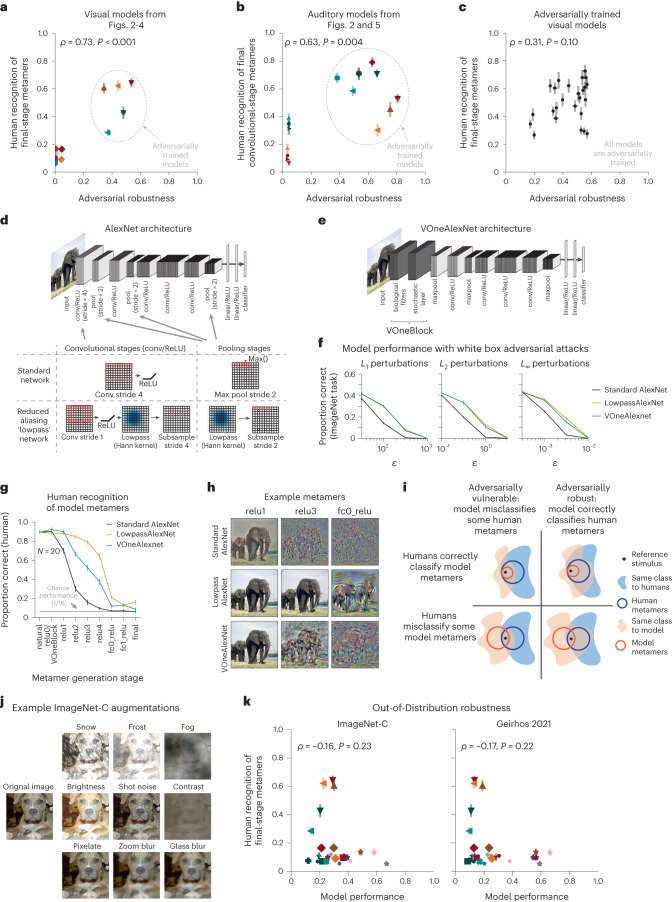
Human recognition of model metamers dissociates from adversarial robustness. **a**, Adversarial robustness of visual models versus human recognizability of final-stage model metamers (*N* = 26 models). Robustness was quantified as average robustness to *L*_2_ (*ε* = 3) and *L*_∞_ (*ε* = 4/255) adversarial examples, normalized by performance on natural images. Symbols follow those in Fig. 7a. Here and in **b** and **c**, error bars for abscissa represent s.e.m. across 5 random samples of 1,024 test examples, and error bars for ordinate represent s.e.m. across participants. **b**, Same as **a** but for final convolutional stage (CochCNN9) or block (CochResNet50) of auditory models (*N* = 17 models). Robustness was quantified as average robustness to *L*_2_ (*ε* = 10^−0.5^) and *L*_∞_ (*ε* = 10^−2.5^) adversarial perturbations of the waveform, normalized by performance on natural audio. Symbols follow those in Fig. 7c. **c**, Adversarial robustness of a set of adversarially trained visual models versus human recognizability of final-stage model metamers (*N* = 25 models). **d**, Operations included in the AlexNet architecture to more closely obey the sampling theorem (the resulting model is referred to as ‘LowpassAlexNet’). **e**, Schematic of VOneAlexNet. **f**, Adversarial vulnerability as assessed via accuracy on a 1,000-way ImageNet classification task with adversarial perturbations of different types and sizes. LowpassAlexNet and VOneAlexNet were equally robust to adversarial perturbations (*F*_1,8_ < 4.5, *P* > 0.1 and ${\eta }_{p}^{2} < 0.36$ for all perturbation types), and both exhibited greater robustness than the standard model (*F*_1,8_ > 137.4, *P* < 0.031 and ${\eta }_{p}^{2} > 0.94$ for all adversarial perturbation types for both architectures). Error bars plot s.e.m. across 5 random samples of 1,024 test images. **g**, Human recognition of model metamers from LowpassAlexNet, VOneAlexNet and standard AlexNet models. LowpassAlexNet had more recognizable metamers than VOneAlexNet (main effect of architecture: *F*_1,19_ = 71.7, *P* < 0.0001, ${\eta }_{p}^{2} > 0.79$; interaction of architecture and model stage: *F*_8,152_ = 21.8, *P* < 0.0001, ${\eta }_{p}^{2} > 0.53$). Error bars plot s.e.m. across participants (*N* = 20). **h**, Example model metamers from the experiment in **d**. **i**, Schematic depiction of how adversarial vulnerability could dissociate from human recognizability of metamers. **j**, Example augmentations applied to images in tests of out-of-distribution robustness. **k**, Scatter plot of out-of-distribution robustness versus human recognizability of final-stage model metamers (*N* = 26 models). Models with large-scale training are denoted with ★ symbols. Other symbols follow those in Fig. 7a; the abscissa value is a single number, and error bars for ordinate represent s.e.m. across participants.

Although adversarial training increased human recognizability of model metamers, the degree of robustness from the training was not itself predictive of metamer recognizability. We first examined all the visual models from Figs. 2–5 and compared their adversarial robustness to the recognizability of their metamers from the final model stage (this stage was chosen because it exhibited considerable variation in recognizability across models). There was a correlation between robustness and metamer recognizability (*ρ* = 0.73, *P* < 0.001), but it was mostly driven by the overall difference between two groups of models, those that were adversarially trained and those that were not (Fig. 6a).

The auditory models showed a similar relationship as the visual models (Fig. 6b). When standard and adversarially trained models were analyzed together, metamer recognizability and robustness were correlated (*ρ* = 0.63, *P* = 0.004), driven by the overall difference between the two groups of models, but there was no obvious relationship when considering just the adversarially trained models.

To further assess whether variations in robustness produce variation in metamer recognizability, we compared the robustness of a large set of adversarially trained models (taken from a well-known robustness evaluation^52^) to the recognizability of their metamers from the final model stage. Despite considerable variation in both robustness and metamer recognizability, the two measures were not significantly correlated (*ρ* = 0.31, *P* = 0.099; Fig. 6c). Overall, it seems that something about the adversarial training procedure leads to more recognizable metamers but that robustness per se does not drive the effect.

Adversarial training is not the only means of making models adversarially robust. But when examining other sources of robustness, we again found examples where a model’s robustness was not predictive of the recognizability of its metamers. Here, we present results for two models with similar robustness, one of which had much more recognizable metamers than the other.

The first model was a CNN that was modified to reduce aliasing (LowpassAlexNet). Because many traditional neural networks contain downsampling operations (for example, pooling) without a preceding lowpass filter, they violate the sampling theorem^25,53^ (Fig. 6d). It is nonetheless possible to modify the architecture to reduce aliasing, and such modifications have been suggested to improve model robustness to small image translations^12,13^. The second model was a CNN that contained an initial processing block inspired by the primary visual cortex in primates^54^. This block contained hard-coded Gabor filters, had noise added to its responses during training (VOneAlexNet; Fig. 6e) and had been previously demonstrated to increase adversarial robustness^55^. A priori, it was unclear whether either model modification would improve human recognizability of the model metamers.

**Extended Data Fig. 9:**
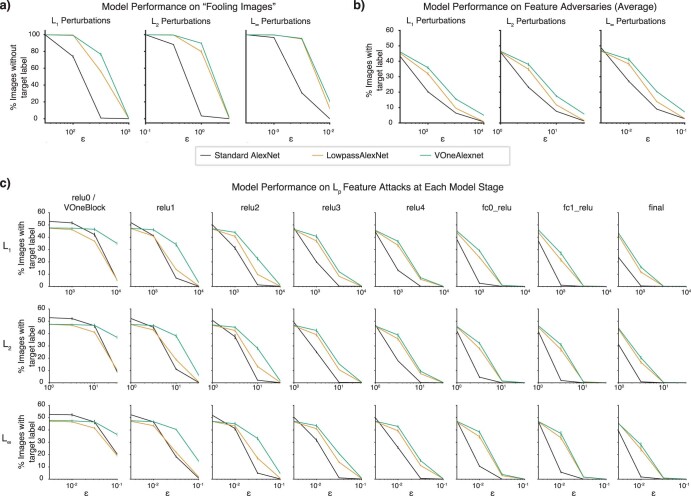
Adversarial robustness of VOneAlexNet and LowPassAlexNet to different types of adversarial examples. **a**, Adversarial robustness to “Fooling images”. Fooling images^14^ are constructed from random noise initializations (the same noise type used for initialization during model metamer generation) by making small *L*_*p*_-constrained perturbations to cause the model to classify the noise as a particular target class. LowpassAlexNet and VOneAlexNet are more robust than the standard AlexNet for all perturbation types (ANOVA comparing VOneAlexNet or LowPassAlexNet to the standard architecture; main effect of architecture; F(1,8) > 6787.0, p < 0.031, ${\eta }_{p}^{2} > 0.999$, for all adversarial perturbation types in both cases), and although there was a significant robustness difference between LowPassAlexNet and VOneAlexNet, it was in the opposite direction as the difference in metamer recognizability: VOneAlexNet was more robust (ANOVA comparing VOneAlexNet to LowPassAlexNet; main effect of architecture; F(1,8) > 98.6, p < 0.031, ${\eta }_{p}^{2} > 0.924$ for all perturbation types). Error bars plot s.e.m. across five sets of target labels. **b**, Adversarial robustness to feature adversaries. Feature adversaries^56^ are constructed by perturbing a natural “source” image so that it yields model activations (at a particular stage) that are close to those evoked by a different natural “target” image, while constraining the perturbed image to remain within a small distance from the original natural image in pixel space. The robustness measure plotted here is averaged across adversaries generated for all stages of a model. LowpassAlexNet and VOneAlexNet were more robust than the standard AlexNet for all perturbation types (ANOVA comparing VOneAlexNet or LowPassAlexNet to the standard architecture; main effect of architecture F(1,8) > 90.8, p < 0.031, ${\eta }_{p}^{2} > 0.919$, for all adversarial perturbation types), and although there was a significant robustness difference between LowPassAlexNet and VOneAlexNet, it was again in the opposite direction as the difference in metamer recognizability: VOneAlexNet was more robust (ANOVA comparing VOneAlexNet to LowPassAlexNet; main effect of architecture; F(1,8) > 69.0, p < 0.031, ${\eta }_{p}^{2} > 0.895$ for all perturbation types). Here and in (c), error bars plot s.e.m. across five samples of target and source images. **c**, Performance on feature adversaries for each model stage used to obtain the average curve in **b**. LowpassAlexNet is not more robust than VOneAlexNet to any type of adversarial example, even though it has more recognizable model metamers (Fig. 6g), illustrating that metamers reveal a different type of model discrepancy than that revealed with typical metrics of adversarial robustness.

Both architectures were comparably robust and more robust than the standard AlexNet to adversarial perturbations (Fig. 6f) as well as ‘fooling images’^14^ (Extended Data Fig. 9a) and ‘feature adversaries’^56^ (Extended Data Fig. 9b,c). However, metamers generated from LowpassAlexNet were substantially more recognizable than metamers generated from VOneAlexNet (Fig. 6g,h). This result provides further evidence that model metamers can differentiate models even when adversarial robustness does not.

These adversarial robustness-related results may be understood in terms of configurations of the four types of stimulus sets originally shown in Fig. 1b (Fig. 6i). Adversarial examples are stimuli that are metameric to a reference stimulus for humans but are classified differently from the reference stimulus by a model. Adversarial robustness thus corresponds to a situation where the human metamers for a reference stimulus fall completely within the set of stimuli that are recognized as the reference class by a model (blue outline contained within the orange shaded region in Fig. 6i, right column). This situation does not imply that all model metamers will be recognizable to humans (orange outline contained within the blue shaded region in the top row). These theoretical observations motivate the use of model metamers as a complementary model test and are confirmed by the empirical observations of this section.

### Metamer recognizability and out-of-distribution robustness

Neural network models have also been found to be less robust than humans to images that fall outside their training distribution (for example, line drawings, silhouettes and highpass-filtered images that qualitatively differ from the photos in the common ImageNet1K training set; Fig. 6j)^10,57,58^. This type of robustness has been found to be improved by training models on substantially larger datasets^59^. We compared model robustness for such ‘out-of-distribution’ images to the recognizability of their metamers from the final model stage (the model set included several models trained on large-scale datasets taken from Fig. 2d, along with all other models from Figs. 2c, 3 and 4). This type of robustness (measured by two common benchmarks) was again not correlated with metamer recognizability (ImageNet-C: *ρ* = –0.16, *P* = 0.227; Geirhos 2021: *ρ* = –0.17, *P* = 0.215; Fig. 6k).

### Metamer recognizability dissociates from model–brain similarity

**Fig. 7:**
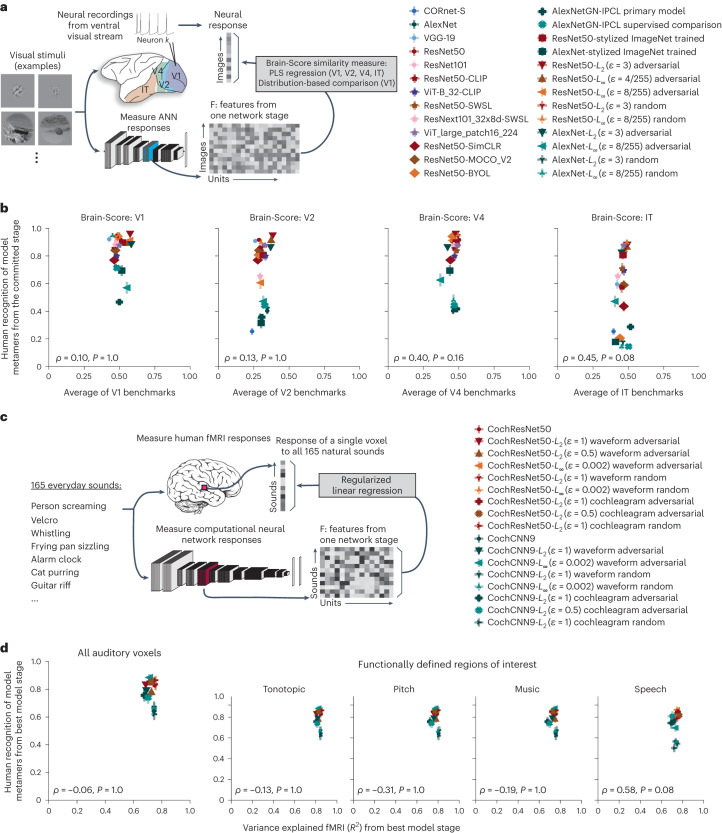
Human recognition of model metamers dissociates from model predictions of brain responses. **a**, Procedure for neural benchmarks; ANN, artificial neural network. **b**, Human recognizability of a model’s metamers versus model–brain similarity for four areas of the ventral stream assessed by a commonly used set of benchmarks^26,31^. The benchmarks mostly consisted of the neurophysiological variance explained by model features via regression. A single model stage was chosen for each model and brain region that produced highest similarity in a separate dataset; graphs plot results for this stage (*N* = 26 models). Error bars on each data point plot s.e.m. across participant metamer recognition; benchmark results are a single number. None of the correlations were significant after Bonferroni correction. Given the split-half reliability of the metamer recognizability scores and the model–brain similarity scores^81^, the noise ceiling of the correlation was *ρ* = 0.92 for IT. **c**, Procedure for auditory brain predictions. Time-averaged unit responses in each model stage were used to predict each voxel’s response to natural sounds using a linear mapping fit to the responses to a subset of the sounds with ridge regression. Model predictions were evaluated on held-out sounds. **d**, Average voxel response variance explained by the best-predicting stage of each auditory model from Figs. 2 and 5 plotted against metamer recognizability for that model stage obtained from the associated experiment. We performed this analysis across all voxels in the auditory cortex (left) and within four auditory functional ROIs (right). Variance explained (*R*^2^) was measured for the best-predicting stage of the models (*N* = 17 models) chosen individually for each participant and ROI (*N* = 8 participants). For each participant, the other participants’ data were used to choose the best-predicting stage. Error bars on each data point plot s.e.m. of metamer recognition and variance explained across participants. No correlations were significant after Bonferroni correction, and they were again below the noise ceiling (the presumptive noise ceiling ranged from *ρ* = 0.78 to *ρ* = 0.87 depending on the ROI).

Are the differences between models shown by metamer recognizability similarly evident when using standard brain comparison benchmarks? To address this question, we used such benchmarks to evaluate the visual and auditory models described above in Figs. 2–5. For the visual models, we used the Brain-Score platform to measure the similarity of model representations to neural benchmarks for visual areas V1, V2 and V4 and the inferior temporal cortex (IT^26,31^; Fig. 7a). The platform’s similarity measure combines a set of model–brain similarity metrics, primarily measures of variance explained by regression-derived predictions. For each model, the score was computed for each visual area using the model stage that gave the highest similarity in held-out data for that visual area. We then compared this neural benchmark score to the recognizability of the model’s metamers from the same stage used to obtain the neural predictions. This analysis showed modest correlations between the two measures for V4 and IT, but these were not significant after Bonferroni correction and were well below the presumptive noise ceiling (Fig. 7b). Moreover, the neural benchmark scores were overall fairly similar across models. Thus, most of the variation in metamer recognizability was not captured by standard model–brain comparison benchmarks.

We performed an analogous analysis for the auditory models using a large dataset of human auditory cortical functional magnetic resonance imaging (fMRI) responses to natural sounds^60^ that had previously been used to evaluate neural network models of the auditory system^4,61^. We analyzed voxel responses within four regions of interest in addition to all of the auditory cortex, in each case again choosing the best-predicting model stage, measuring the variance it explained in held-out data and comparing that to the recognizability of the metamers from that stage (Fig. 7c). The correlation between metamer recognizability and explained variance in the brain response was not significant when all voxels were considered (*ρ* = –0.06 and *P* = 1.0 with Bonferroni correction; Fig. 7d). We did find a modest correlation within one of the regions of interest (ROIs; speech: *ρ* = 0.58 and *P* = 0.08 with Bonferroni correction), but it was well below the presumptive noise ceiling (*ρ* = 0.78).

**Extended Data Fig. 10:**
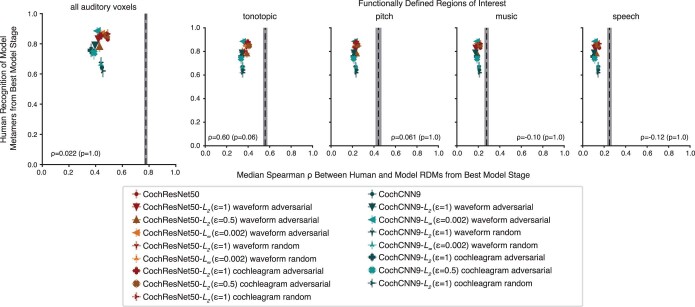
Representational Similarity Analysis of auditory fMRI data. The median Spearman ρ between the RDM from fMRI activations to natural sounds (N = 8 participants) and the RDM from model activations at the best model stage as determined with held-out data, compared with the metamer recognition by humans at this chosen model stage. The dashed black line shows the upper bound on the RDM similarity that could be measured given the data reliability, estimated by comparing a participant’s RDM with the average of the RDMs from each of the other participants. Error bars are s.e.m. across participants. The correlation between metamer recognizability and the human-model RDM similarity was not statistically significant for any of the ROIs following Bonferroni correction (all: ρ = 0.02, p = 1.0; tonotopic: ρ = 0.60, p = .06; pitch: ρ = 0.06, p = 1.0; music: ρ = 0.10, p = 1.0; speech: ρ = 0.12, p = 1.0), and was again well below the presumptive noise ceiling (which ranged from ρ = 0.79 to ρ = 0.89, depending on the ROI). We also note that the variation in metamer recognizability across models is substantially greater than the variation in RDM similarity, indicating that metamers better differentiate this set of models than does the RDM similarity with this fMRI dataset.

We conducted analogous analyses using representational similarity analysis instead of regression-based explained variance to evaluate auditory model–brain similarity; these analyses yielded similar conclusions as the regression-based analyses (Extended Data Fig. 10). Overall, the results indicate that the metamer test is complementary to traditional metrics of model–brain fit (and often distinguishes models better than these traditional metrics).

### Metamer transfer across models

**Fig. 8:**
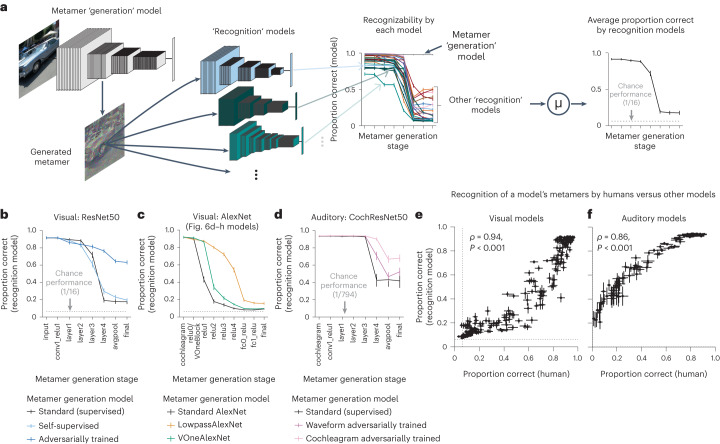
Human recognition of a model’s metamers is correlated with their recognition by other models. **a**, Model metamers were generated for each stage of a ‘generation’ model (one of the models from Figs. 2c,d, 3, 4 and 6g for visual models and from Figs. 2f and 5 for auditory models). These metamers were presented to ‘recognition’ models (all other models from the listed figures). We measured recognition of the generating model’s metamers by each recognition model, averaging accuracy over all recognition models (excluding the generation model), as shown here for a standard-trained ResNet50 image model. Error bars represent s.e.m. over *N* = 28 recognition models. **b**, Average model recognition of metamers from the standard ResNet50, the three self-supervised ResNet50 models and the three adversarially trained ResNet50 models. To obtain self-supervised and adversarially trained results, we averaged each recognition model’s accuracy curve across all generating models and averaged these curves across recognition models. Error bars represent s.e.m. over *N* = 28 recognition models for standard models and *N* = 29 recognition models for adversarially trained and self-supervised models. **c**, Same as **b** but for Standard AlexNet, LowpassAlexNet and VOneAlexNet models from Fig. 6d–h. Error bars are over *N* = 28 recognition models. **d**, Same as **b** but for auditory models, with metamers generated from the standard CochResNet50, the three CochResNet50 models with waveform adversarial perturbations and the two CochResNet50 models with cochleagram adversarial perturbations. Chance performance is 1/794 for models because they had a ‘null’ class in addition to 793 word labels. Error bars represent s.e.m. over *N* = 16 recognition models for the standard model and *N* = 17 recognition models for adversarially trained models. **e**,**f**, Correlation between human and model recognition of another model’s metamers for visual (**e**; *N* = 219 model stages) and auditory (**f**; *N* = 144 model stages) models. Abscissa plots average human recognition accuracy of metamers generated from one stage of a model, and error bars represent s.e.m. across participants. Ordinate plots average recognition by other models of those metamers, and error bars represent s.e.m. across recognition models. Human recognition of a model’s metamers is highly correlated with other models’ recognition of those same model metamers.

Are one model’s metamers recognizable by other models? We addressed this issue by taking all the models trained for one modality, holding one model out as the ‘generation’ model and presenting its metamers to each of the other models (‘recognition’ models), measuring the accuracy of their class predictions (Fig. 8a). We repeated this procedure with each model as the generation model. As a summary measure for each generation model, we averaged the accuracy across the recognition models (Fig. 8a and Supplementary Figs. 3 and 4). To facilitate comparison, we analyzed models that were different variants of the same architecture. We used permutation tests to evaluate differences between generation models (testing for main effects).

Metamers from late stages of the standard-trained ResNet50 were generally not recognized by other models (Fig. 8b). A similar trend held for the models trained with self-supervision. By contrast, metamers from the adversarially trained models were more recognizable to other models (Fig. 8b; *P* < 0.0001 compared to either standard or self-supervised models). We saw an analogous metamer transfer boost from the model with reduced aliasing (LowpassAlexNet), for which metamers for intermediate stages were more recognizable to other models (Fig. 8c; *P* < 0.0001 compared to either standard or VOneAlexNet models). Similar results held for auditory models (Fig. 8d; waveform adversarially trained versus standard, *P* = 0.011; cochleagram adversarially trained versus standard, *P* < 0.001), although metamers from the standard-trained CochResNet50 transferred better to other models than did those for the supervised vision model, perhaps due to the shared cochlear representation present in all auditory models, which could increase the extent of shared invariances.

These results suggest that models tend to contain idiosyncratic invariances, in that their metamers vary in ways that render them unrecognizable to other models. This finding is loosely consistent with findings that the representational dissimilarity matrices for natural images can vary between individual neural network models^62^. The results also clarify the effect of adversarial training. Specifically, they suggest that adversarial training removes some of the idiosyncratic invariances of standard-trained deep neural networks rather than learning new invariances that are not shared with other models (in which case their metamers would not have been better recognized by other models). The architectural change that reduced aliasing had a similar effect, albeit limited to the intermediate model stages.

The average model recognition of metamers generated from a given stage of another model is strikingly similar to human recognition of the metamers from that stage (compare Fig. 8b–d to Figs. 3c, 4b, 5f and 6g). To quantify this similarity, we plotted the average model recognition for metamers from each stage of each generating model against human recognition of the same stimuli, revealing a strong correlation for both visual (Fig. 8e) and auditory (Fig. 8f) models. This result suggests that the human–model discrepancy revealed by model metamers reflects invariances that are often idiosyncratic properties of a specific neural network, leading to impaired recognition by both other models and human observers.

## Discussion

We used model metamers to reveal invariances of deep artificial neural networks and compared these invariances to those of humans by measuring human recognition of visual and auditory model metamers. Metamers of standard deep neural networks were dominated by invariances that are absent from human perceptual systems, in that metamers from late model stages were typically completely unrecognizable to humans. This was true across modalities (visual and auditory) and training methods (supervised versus self-supervised training). The effect was driven by invariances that are idiosyncratic to a model, as human recognizability of a model’s metamers was well predicted by their recognizability to other models. We identified ways to make model metamers more human-recognizable in both the auditory and visual domains, including a new type of adversarial training for auditory models using perturbations at an intermediate model stage. Although there was a substantial metamer recognizability benefit from one common training method to reduce adversarial vulnerability, we found that metamers revealed model differences that were not evident by measuring adversarial vulnerability alone. Moreover, the model improvements revealed by model metamers were not obvious from standard brain prediction metrics. These results show that metamers provide a model comparison tool that complements the standard benchmarks that are in widespread use. Although some models produced more recognizable metamers than others, metamers from late model stages remained less recognizable than natural images or sounds in all cases we tested, suggesting that further improvements are needed to align model representations with those of biological sensory systems.

Might humans analogously have invariances that are specific to an individual? This possibility is difficult to explicitly test given that we cannot currently sample human metamers (metamer generation relies on having access to the model’s parameters and responses, which are currently beyond reach for biological systems). If idiosyncratic invariances were also present in humans, the phenomenon we have described here might not represent a human–model discrepancy and could instead be a common property of recognition systems. The main argument against this interpretation is that several model modifications (different forms of adversarial training and architectural modifications to reduce aliasing) substantially reduced the idiosyncratic invariances present in standard deep neural network models. These results suggest that idiosyncratic invariances are not unavoidable in a recognition system. Moreover, the set of modifications explored here was far from exhaustive, and it seems plausible that idiosyncratic invariances could be further alleviated with alternative training or architecture changes in the future.

### Relation to previous work

Previous work has also used gradient descent on the input to visualize neural network representations^51,63^. However, the significance of these visualizations for evaluating neural network models of biological sensory systems has received little attention. One contributing factor may be that model visualizations have often been constrained by added natural image priors or other forms of regularization^64^ that help make visualizations look more natural but mask the extent to which they otherwise diverge from a perceptually meaningful stimulus. By contrast, we intentionally avoided priors or other regularization when generating model metamers, as they defeat the purpose of the metamer test. When we explicitly measured the benefit of regularization, we found that it did boost recognizability somewhat but that it was not sufficient to render model metamers fully recognizable or reproduce the benefits of model modifications that improve metamer recognizability (Fig. 4e).

Another reason the discrepancies we report here have not been widely discussed within neuroscience is that most studies of neural network visualizations have not systematically measured recognizability to human observers (in part because these visualizations are primarily reported within computer science, where such experiments are not the norm). We found controlled experiments to be essential. Before running full-fledged experiments, we always conducted the informal exercise of generating examples and evaluating them subjectively. Although the largest effects were evident informally, the variability of natural images and sounds made it difficult to predict with certainty how an experiment would turn out. It was thus critical to substantiate informal observation with controlled experiments in humans.

Metamers are also methodologically related to a type of adversarial example generated by adding small perturbations to an image from one class such that the activations of a classifier (or internal stage) match those of a reference image from a different class^56,65^, despite being seen as different classes by humans when tested informally^66,67^. Our method differs in probing model invariances without any explicit bias to cause metamers to appear different to humans. We found models in which vulnerability to these adversarial examples dissociated from metamer recognizability (Extended Data Fig. 9), suggesting that metamers may reflect distinct model properties.

### Effects of unsupervised training

Unsupervised learning potentially provides a more biologically plausible computational theory of learning^41,68^ but produced qualitatively similar model metamers as supervised learning. This finding is consistent with evidence that the classification errors of self-supervised models are no more human-like than those of supervised models^69^. The metamer-related discrepancies are particularly striking for self-supervised models because they are trained with the goal of invariance, being explicitly optimized to become invariant to the augmentations performed on the input. We also found that the divergence with human recognition had a similar dependence on model stage irrespective of whether models were trained with or without supervision. These findings raise the possibility that factors common to supervised and unsupervised neural networks underlie the divergence with humans.

### Differences in metamers across stages

The metamer test differs from some other model metrics (for example, behavioral judgments of natural images or sounds, or measures of adversarial vulnerability) in that metamers can be generated from every stage of a model, with the resulting discrepancies associated with particular model stages. For instance, metamers revealed that intermediate stages were more human-like in some models than others. The effects of reducing aliasing produced large improvements in the human recognizability of metamers from intermediate stages (Fig. 6g), consistent with the idea that biological systems also avoid aliasing. By contrast, metamers from the final stages showed little improvement. This result indicates that this model change produces intermediate representations with more human-like invariances despite not resolving the discrepancy introduced at the final model stages. The consistent discrepancies at the final model stages highlight these late stages as targets for model improvements^45^.

For most models, the early stages produced model metamers that were fully recognizable but that also resemble the original image or sound they were matched to. By contrast, metamers from late stages physically deviated from the original image or sound but for some models nonetheless remained recognizable. This difference highlights two ways that a model’s metamers can pass the recognition test used here, either by being perceptually indistinguishable to humans or by being recognizable to humans as the same class despite being perceptually distinct. This distinction could be quantified in future work by combining a traditional metamer test with our recognition test.

### Limitations

Although a model that fails our metamer test is ruled out as a description of human perception, passing the test on its own reveals little. For instance, a model that instantiates the identity mapping would pass our test despite not being able to account for human perceptual abilities. Traditional metrics thus remain critical but on their own are also insufficient (as shown in Figs. 6 and 7). Failing the test also does not imply that the model representations are not present in the brain, only that they are not sufficient to account for the recognition behavior under consideration. For instance, there is considerable evidence for time-averaged auditory statistics in auditory perception^19,70^ even though they do not produce human-recognizable metamers for speech (Extended Data Fig. 5c). The results point to the importance of a large suite of test metrics for model comparison, including, but not limited to, the model metamer test.

Model metamers are generated via gradient-based optimization of a non-convex loss function and only approximately reproduce the activations of the natural stimulus to which they are matched. We attempted to improve on previous neural network visualization work^51,63^ by setting explicit criteria for optimization success (Fig. 1e and Extended Data Fig. 4). However, the reliance on optimization may be a limitation in some contexts and with some models.

The metamer optimization process is also not guaranteed to sample uniformly from the set of a model’s metamers. Non-uniform sampling cannot explain the human–model discrepancies we observed but could in principle contribute to differences between the magnitude of discrepancies for some models compared to others, for instance if differences in the optimization landscape make it more or less likely that the metamer generation process samples along a model’s idiosyncratic invariances. We are not aware of any reason to think that this might be the case, but it is not obvious how to fully exclude this possibility.

### Future directions

The underlying causes of the human–model discrepancies demonstrated here seem important to understand, both because they may clarify biological sensory systems and because many potential model applications, such as model-based signal enhancement^71,72^, are likely to be hindered by human-discrepant model invariances. The results of Fig. 8 (showing that human recognition of a model’s metamers can be predicted by the recognition judgments of a set of other models) suggest a way to efficiently screen for discrepant metamers, which should facilitate evaluation of future models.

One explanation for the human–model discrepancies we observed could be that biological sensory systems do not instantiate invariances per se in the sense of mapping multiple different inputs onto the same representation^73,74^. Instead, they might learn representations that ‘untangle’ behaviorally relevant variables. For instance, a system could represent word labels and talker identity or object identity and pose via independent directions in a representational space. Such a representational scheme could enable invariant classification without invariant representations and might be facilitated by training on multiple tasks or objectives (rather than the single tasks/objectives used for the models we tested). Alternative model architectures may also help address this hypothesis. In particular, ‘generative’ models that estimate the probability of an input signal given a latent variable (rather than the probability of a latent variable for a given input signal as in the ‘discriminative’ models studied here) seem likely to mitigate the metamer discrepancies we found. There are indications that adding generative training objectives can improve the alignment of model representations with humans in models trained on small-scale tasks^75^. But currently, we lack methods for building such models that can support human-level recognition at scale^76,77^.

The discrepancies shown here for model metamers contrast with a growing number of examples of human–model similarities for behavioral judgments of natural stimuli. Models optimized for object recognition^78^, speech recognition^4^, sound localization^79^ and pitch recognition^80^ all exhibit qualitative and often quantitative similarities to human judgments when run in traditional psychophysical experiments with natural or relatively naturalistic stimuli (that fall near their training distribution). However, these same models can exhibit inhuman behavior for signals that fall outside the distribution of natural sounds and images, particularly those derived from the model.

Current deep neural network models are overparametrized, such that training produces one of many functions consistent with the training data. From this perspective it is unsurprising that different systems can perform similarly on natural signals while exhibiting different responses to signals outside the training distribution of natural images or sounds. Yet, we nonetheless found that sensible engineering modifications succeeded in bringing models into better alignment with human invariances. These results demonstrate that divergence between human and model invariances is not inevitable and show how metamers can be a useful metric to guide and evaluate the next generation of brain models.

## Methods

All experiments with human participants (both online and in the lab) were approved by the Committee On the Use of Humans as Experimental Subjects at the Massachusetts Institute of Technology (MIT) and were conducted with the informed consent of the participants.

### Model implementation

Models were implemented in the PyTorch deep learning library^82^ and obtained through publicly available checkpoints or trained by authors on the MIT OpenMind computing cluster. All models and analysis used Python 3.8.2 and PyTorch 1.5.0, except in cases where models or graphics processing unit hardware required operations not present in PyTorch 1.5.0, in which case we used PyTorch 1.12.1. Details of all Python dependencies and package versions are provided in the form of a conda environment at https://github.com/jenellefeather/model_metamers_pytorch.

Additional details of model training and evaluation are provided in Supplementary Modeling Information Note 1 and Supplementary Tables 3 and 4. Full architecture descriptions are provided in Supplementary Modeling Information Note 2.

### Metamer generation

#### Optimization of metamers

Gradient descent was performed on the input signal to minimize the normalized squared error between all activations at a particular model stage (for instance, each *x*, *y* and *channel* value from the output of a convolutional layer) for the model metamer and the corresponding activations for a natural signal

$$\frac{\Vert A-A^{\prime} \Vert }{\Vert A\Vert },$$

where *A* represents the activations from the natural signal, and *A*′ represents the activations from the model metamer (that is, sampling from the preimage of the model activations at the generation stage). The weights of the model remained fixed during the optimization. Each step of gradient descent was constrained to have a maximum *L*_2_ norm of *η*, where *η* was initialized at 1 and was dropped by a factor of 0.5 after every 3,000 iterations. Optimization was run for a total of 24,000 steps for each generated metamer. The shape of the input stimuli, range of the input and any normalization parameters were matched to those used for testing the model on natural stimuli. Normalization that occurred after data augmentation in the visual models (subtracting channel means and dividing by channel standard deviations) was included as a model component during metamer generation (that is, gradients from these operations contributed to the metamer optimization along with all other operations in the model). For vision models, the input signal was initialized as a sample from a normal distribution with a standard deviation of 0.05 and a mean of 0.5 (or a standard deviation of 10 and a mean of 127.5 in the case of HMAX). For auditory models, the input signal was initialized from a random normal distribution with a standard deviation of 10^−7^ and a mean of 0 (or a standard deviation of 10^−5^ and a mean of 0 in the case of Spectemp).

#### Criteria for optimization success

Because metamers are derived via a gradient descent procedure, the activations that they produce approach those of the natural signal used to generate them but never exactly match. It was thus important to define criteria by which the optimization would be considered sufficiently successful to include the model metamer in the behavioral experiments.

The first criterion was that the activations for the model metamer had to be matched to those for the natural signal better than would be expected by chance. We measured the fidelity of the match between the activations for the natural stimulus and its model metamer at the matched model stage using three different metrics: Spearman *ρ*, Pearson *R*^2^ and the SNR,

$${\rm{SN}}{{\rm{R}}}_{{\rm{dB}}}=10{\rm{lo}}{{\rm{g}}}_{10}\frac{\sum \left({{\rm{x}}}^{2}\right)}{\sum \left[{\left({\rm{x}}-{\rm{y}}\right)}^{2}\right]},$$

where *x* is the activations for the original sound when comparing metamers or for a randomly selected sound for the null distribution, and *y* is activations for the comparison sound (the model metamer or another randomly selected sound). We then ensured that for each of the three measures, the value for the model metamer fell outside of a null distribution measured between 1,000,000 randomly chosen image or audio pairs from the training dataset. Metamers that did not pass the null distribution test for any of the Spearman *ρ*, Pearson *R*^2^ or SNR values measured at the stage used for the optimization were excluded from the set of experimental stimuli. The only exception to this was the HMAX model, for which we only used the SNR for the matching criteria.

The second criterion was that the models had to produce the same class label for the model metamer and natural signal. For visual models, the model metamer had to result in the same 16-way classification label as the natural signal to which it was matched. For the auditory models, the model metamer had to result in the same word label (of 794 possible labels, including ‘null’) as the natural speech signal to which it was matched. For models that did not have a classifier stage (the self-supervised models, HMAX and the spectrotemporal filter model), we trained a classifier as described in Supplementary Modeling Note 1 for this purpose. The classifier was included to be conservative but in practice could be omitted in future work, as very few stimuli pass the first matching fidelity criterion but not the classifier criterion.

#### Handling gradients through the ReLU operation

Many neural networks use the ReLU nonlinearity, which yields a partial derivative of 0 if the input is negative. We found empirically that it was difficult to match ReLU layers due to the initialization producing many activations of 0. To improve the optimization when generating a metamer for activations immediately following a ReLU, we modified the derivative of the metamer generation layer ReLU to be 1 for all input values, including values below 0 (ref. ^25^). ReLU layers that were not the metamer generation layer behaved normally, with a gradient of 0 for input values below 0.

#### Metamer generation with regularization

To investigate the effects of regularization on metamer recognizability, we generated metamers with additional constraints on the optimization procedure. We followed the procedures of an earlier paper by Mahendran and Vedaldi^51^. Two regularization terms were included: (1) a total variation (TV) regularizer and (2) an *α*-norm regularizer.

The resulting objective function minimized to generate metamers was

$$\frac{\Vert A-A^{\prime} \Vert }{\Vert A^{\prime} \Vert }+{\lambda }_{\alpha }{R}_{\alpha }(x)+{\lambda }_{{\mathrm{TV}}}{R}_{{\mathrm{TV}}}(x)$$

using the 6-norm for the *α*-norm regularizer

$${R}_{\alpha }(x)={\Vert x-\bar{x}\Vert }_{6}$$

and using the TV regularizer

$${R}_{\mathrm{TV}}\left(x\right)=\sum _{i,\,j}\left({\left[{\left(x-{\bar{x}}\right)}_{i,\,j+1}-{\left(x-{\bar{x}}\right)}_{i,\,j}\right]}^{2}+{\left[{(x-{\bar{x}})}_{i+1,\,j}-{\left(x-{\bar{x}}\right)}_{i,\,j}\right]}^{2}\right),$$

where *A* represents the activations evoked by the natural signal at the generation layer, *A*′ represents the activations evoked by the model metamer at the generation layer, *λ*_*α*_ and *λ*_TV_ are scaling coefficients for the regularizers, and $\bar{x}$ is the mean of the input signal *x* (*x* is normalized according to the typical normalization for the model, subtracting the dataset mean and dividing by the dataset standard deviation for each channel).

For the TV regularizer, we generated metamers for three different coefficient values with ${{\lambda }_{{{\mathrm{TV}}}}}_{1}=0.000005$, ${{\lambda }_{{{\mathrm{TV}}}}}_{2}=10{{\lambda }_{{{\mathrm{TV}}}}}_{1}$ and ${{\lambda }_{{{\mathrm{TV}}}}}_{3}=100{{\lambda }_{{{\mathrm{TV}}}}}_{1}$. As observed by Mahendran and Vedaldi^51^, we found that larger TV regularizers impaired optimization at early model stages, with resulting stimuli often not passing our metamer optimization success criteria (for instance, only 2/400 metamers generated from relu0 of AlexNet passed this criteria for the largest regularizer coefficient value). Thus, for the behavioral experiments, we chose separate coefficient values for each model stage. Specifically, in AlexNet, we used ${{\lambda }_{{{\mathrm{TV}}}}}_{1}$ for relu0 and relu1, ${{\lambda }_{{{\mathrm{TV}}}}}_{2}$ for relu2 and relu3 and ${{\lambda }_{{{\mathrm{TV}}}}}_{3}$ for relu4, fc0_relu, fc1_relu and final (this is exactly what was done in Mahendran and Vedaldi^51^ except that the *λ* values are different due to differences in how the input is normalized, 0–255 in Mahendran and Vedaldi^51^ compared to 0–1 in our models).

For the *α*-norm regularizer, we followed the methods used by Mahendran and Vedaldi^51^, with *α* = 6, and used a single coefficient of *λ*_*α*_ = 0.005 for all stages. This coefficient was chosen based on the logic proposed in Mahendran and Vedaldi^51^ for the starting value, with a small sweep around the values for a small number of examples (10× up and 10× down), in which we subjectively judged which value produced the largest visual recognizability benefit.

We observed that when these regularizers were used, the default step sizes (initial learning rate of *η* = 1) used in our metamer generation method resulted in stimuli that looked qualitatively more ‘gray’ than expected, that is, stayed close to the mean. Thus, to maximize the chances of seeing a benefit from the regularization, in a separate condition, we increased the initial step size for metamer generation to be 16 times the default value (initial *η* = 16).

We found empirically that there was a trade-off between satisfying the goal of matching the metamer activations and minimizing the regularization term. As described above, it was necessary to hand-tune the regularization weights to obtain something that met our convergence criteria, but even when these criteria were met, metamers generated with regularization tended to have worse activation matches than metamers generated without regularization. This observation is consistent with the idea that there is not an easy fix to the discrepancies revealed by metamers that simply involves adding an additional term to the optimization. And in some domains (such as audio), it is not obvious what to use for a regularizer. Although the use of additional criteria to encourage the optimization to stay close to the manifold of ‘natural’ examples likely has useful applications, we emphasize that it is at odds with the goal of testing whether a model on its own replicates the properties of a biological sensory system.

### Behavioral experiments

All behavioral experiments presented in the main text were run on Amazon Mechanical Turk. To increase data quality, Amazon Turk qualifications were set to relatively stringent levels. The ‘HIT Approval Rate for all Requesters’ HITs’ had to be at least 97%, and the ‘Number of HITs Approved’ had to exceed 1,000. Blinding was not applicable as the analysis was automated. Example code to run the online experiments is available at https://github.com/jenellefeather/model_metamers_pytorch.

#### Stimuli: image experiments

Each stimulus belonged to 1 of the 16 entry-level Microsoft Common Objects in Context categories. We used a mapping from these 16 categories to the corresponding ImageNet1K categories (where multiple ImageNet1K categories can map onto a single Microsoft Common Objects in Context category), used in a previous publication^10^. For each of the 16 categories, we selected 25 examples from the ImageNet1K validation dataset for a total of 400 natural images that were used to generate stimuli. A square center crop was taken for each ImageNet1K image (with the smallest dimension of the image determining the size), and the square image was rescaled to the necessary input dimensions for each ImageNet1K-trained model. Metamers were generated for each of the 400 images to use for the behavioral experiments.

#### Stimuli: auditory experiments

Stimuli were generated from 2-s speech audio excerpts randomly chosen from the test set of the Word–Speaker–Noise dataset^25^ (Supplemental Modeling Note 1) constrained such that only clips from unique sources within the Wall Street Journal corpus were used. Sounds were cropped to the middle 2 s of the clip such that the labeled word was centered at the 1-s mark. To reduce ambiguity about the clip onset and offset, we also screened to ensure that the beginning and end 0.25 s of the clip was no more than 20 dB quieter than the full clip. Four hundred clips were chosen subject to these constraints and such that each clip contained a different labeled word. Metamers were generated for each of the 400 clips.

#### Image behavioral experiment

We created a visual experiment in JavaScript similar to that described in a previous publication^10^. Participants were tasked with classifying an image into 1 of 16 presented categories (airplane, bear, bicycle, bird, boat, bottle, car, cat, chair, clock, dog, elephant, keyboard, knife, oven and truck). Each category had an associated image icon that participants chose from during the experiment. Each trial began with a fixation cross at the center of the screen for 300 ms, followed by a natural image or a model metamer presented at the center of the screen for 300 ms, a pink noise mask presented for 300 ms and a 4 × 4 grid containing all 16 icons. Participants selected an image category by clicking on the corresponding icon. To minimize effects of internet disruptions, we ensured that the image was loaded into the browser cache before the trial began. To assess whether any timing variation in the online experiment setup might have affected overall performance, we compared recognition performance on natural images to that measured during in-lab pilot experiments (with the same task but different image examples) reported in an earlier conference paper^25^. The average online performance across all natural images was on par or higher than that measured in the lab (in-lab proportion correct = 0.888 ± 0.0240) for all experiments.

The experimental session began with 16 practice trials to introduce participants to the task with 1 trial for each category, each presenting a natural image from the ImageNet1K training set. Participants received feedback for these first 16 trials. Participants then began a 12-trial demo experiment that contained some natural images and some model metamers generated from the ImageNet1K training set. The goal of this demo experiment was twofold: (1) to introduce participants to the types of stimuli they would see in the main experiment and (2) to be used as a screening criterion to remove participants who were distracted, misunderstood the task instructions, had browser incompatibilities or were otherwise unable to complete the task. Participants were only allowed to start the main experiment if they correctly answered 7 of 12 correct on the demo experiment, which was the minimum that were correctly answered for these same demo stimuli by 16 in-lab participants in a pilot experiment^25^. In total, 341 of 417 participants passed the demo experiment and chose to move on to the main experiment. Participants received $0.50 for completing the demo experiment.

There were 12 different online image experiments, each including a set of conditions (model stages) to be compared. Participants only saw 1 natural image or metamer for each of the 400 images in the behavioral stimulus set. Participants additionally completed 16 catch trials consisting of the icon image for one of the classes. Participant data were only included in the analysis if the participant got 15 of 16 of these catch trials correct (270 of 341 participants were included across the 12 experiments). Of these participants, 125 self-identified as female, 143 self-identified as male, and 2 did not report. The mean age was 42.1 years, the minimum age was 20 years, and the maximum age was 78 years. For all but the HMAX experiment, participants completed 416 trials, 1 for each of the 400 original images plus the 16 catch trials. The 400 images were randomly assigned to the experiment conditions subject to the constraint that each condition had approximately the same number of trials (Supplementary Table 5). The resulting 416 total trials were then presented in random order across the conditions of the experiment. The HMAX experiment used only 200 of the original 400 images for a total of 216 trials. Participants received an additional $6.50 for completing the experiment (or $3.50 in the case of HMAX).

Model performance on this 16-way classification task was evaluated by measuring the predictions for the full 1,000-way ImageNet classification task and finding the maximum probability for a label that was included in the 16-class dataset (231 classes).

#### Auditory behavioral experiment

The auditory experiment was similar to that used in earlier publications^4,25^. Each participant listened to a 2-s audio clip and chose 1 of 793 word labels corresponding to the word in the middle of the clip (centered at the 1-s mark of the clip). Responses were entered by typing the word label into a response box. As participants typed, word labels matching the letter string they were typing appeared below the response box to help participants identify allowable responses. Once a word was typed that matched 1 of the 793 responses, participants could move on to the next trial.

To increase data quality, participants first completed a short experiment (six trials) that screened for the use of headphones^83^. Participants received $0.25 for completing this task. If participants scored five of six or higher on this screen (224/377 participants), they moved on to a practice experiment consisting of ten natural audio trials with feedback (drawn from the training set) designed to introduce the task. This was followed by a demo experiment of 12 trials without feedback. These 12 trials contained both natural audio and model metamers^25^. The audio demo experiment served to introduce participants to the types of stimuli they would hear in the main experiment and to screen out poorly performing participants. A screening criterion was set at 5 of 12, which was the minimum for 16 in-lab participants in earlier work^25^. In total, 154 of 224 participants passed the demo experiment and chose to move on to the main experiment. Participants received an additional $0.50 for completing the demo experiment. We have repeatedly found that online auditory psychophysical experiments qualitatively and quantitatively reproduce in-lab results, provided that steps such as these are taken to help ensure good audio presentation quality and attentive participants^84–87^. Here, we found that average online performance on natural stimuli was comparable to in-lab performance reported in Feather et al.^25^ using the same task with different audio clips (in-lab proportion correct = 0.863 ± 0.0340).

There were six different main auditory experiments. The design of these experiments paralleled that of the image experiments. Participants only heard 1 natural speech or metamer stimulus for each of the 400 excerpts in the behavioral stimulus set. Participants additionally completed 16 catch trials. These catch trials each consisted of a single word corresponding to one of the classes. Participant data were only included in the analysis if the participant got 15 of 16 of these trials correct (this inclusion criterion removed 8 of 154 participants). Some participants chose to leave the experiment early and were excluded from the analysis (23 of 154), and 3 participants were excluded due to self-reported hearing loss, yielding a total of 120 participants across all auditory experiments. Of these participants, 45 self-identified as female, 68 self-identified as male, and 7 chose not to report (mean age = 39 years, minimum age = 22 years, maximum age = 77 years). For all but the Spectemp experiment, participants completed 416 trials, 1 for each of the 400 original excerpts, plus the 16 catch trials. The 400 excerpts were randomly assigned to the experiment conditions subject to the constraint that each condition had approximately the same number of trials (Supplementary Table 6). The resulting 416 total trials were then presented in random order across the conditions of the experiment. The Spectemp experiment used only 200 of the original 400 excerpts for a total of 216 trials. We collected online data in batches until we reached the target number of participants for each experiment. Participants received $0.02 cents for each trial completed plus an additional $3.50 bonus for completing the full experiment (or $2.00 for the Spectemp experiment).

#### Statistical tests: difference between human and model recognition accuracy

Human recognition experiments were analyzed by comparing human recognition of a generating model’s metamers to the generating model’s recognition of the same stimuli (its own metamers). Each human participant was run on a distinct set of model metamers; we presented each set to the generation model and measured its recognition performance for that set. Thus, if *N* human participants performed an experiment, we obtained *N* model recognition curves. We ran mixed-model, repeated measures ANOVAs with a within-group factor of metamer generation model stage and a between-group factor of observer (human or model observer), testing for both a main effect of observer and an interaction between observer and model stage. Data were non-normal due to a prevalence of values close to 1 or 0 depending on the condition, and so we evaluated statistical significance non-parametrically using permutation tests comparing the observed *F* statistic to that obtained after randomly permuting the data labels. To test for main effects, we permuted observer labels (model versus human). To test for interactions of observer and model stage, we permuted both observer labels and model stage labels independently for each participant. In each case, we used 10,000 random permutations and computed a *P* value by comparing the observed *F* statistic to the null distribution of *F* statistics from permuted data (that is, the *P* value was 1 – rank of the observed *F* statistic/number of permutations). *F* statistics here and elsewhere were calculated with MATLAB 2021a.

Because the classical models (Extended Data Figs. 4 and 5) did not perform recognition judgments, rather than comparing human and model recognition as in the experiments involving neural network models, we instead tested for a main effect of model stage on human observer recognition. We performed a single-factor repeated measures ANOVA using a within-group factor of model stage, again evaluating statistical significance non-parametrically (we randomly permuted the model stage labels of the recognition accuracy data, independently for each participant, with 10,000 random permutations).

#### Statistical tests: difference between human recognition of metamers generated from different models

To compare human recognition of metamers generated from different models, we ran a repeated measures ANOVA with within-group factors of model stage and generating model. This type of comparison was only performed in cases where the generating models had the same architecture (so that the model stages were shared between models). We again evaluated statistical significance non-parametrically by comparing the observed *F* statistic to a null distribution of *F* statistics from permuted data (10,000 random permutations). To test for a main effect of generating model, we randomly permuted the generating model label independently for each participant. To test for an interaction between generating model and model stage, we permuted both generating model and model stage labels independently for each participant.

#### Power analysis to determine sample sizes

To estimate the number of participants necessary to be well powered for the planned statistical tests, we ran a pilot experiment comparing the standard versus adversarially trained ResNet50 and CochResNet50 models, as this experiment included the largest number of conditions, and we expected that differences between different adversarially trained models would be subtle, putting an upper bound on the sample sizes needed across experiments.

For the vision experiment, we ran ten participants in a pilot experiment on Amazon Mechanical Turk. The format was identical to that of the main experiments described here, with the exception that we used a screening criterion of 8 of 12 correct for the pilot rather than the 7 of 12 correct used for the main experiment. In this pilot experiment, the smallest effect size out of those we anticipated analyzing in the main experiments was the comparison between the *L*_∞_-norm (*ε* *=* 8/256) adversarially trained ResNet50 and the *L*_2_-norm (*ε* = 3) adversarially trained Resnet50 with a partial *η*^2^ value of 0.10 for the interaction. A power analysis with G*Power^88^ showed that 18 participants were needed to have a 95% chance of seeing an effect of this size at a *P* < 0.01 significance level. We thus set a target of 20 participants for each online vision experiment.

For the auditory experiments, we ran 14 participants in a pilot experiment on Amazon Mechanical Turk. The format was identical to that of the main experiments in this paper with the exception that 8 of the 14 participants only received six original audio trials with feedback, whereas in the main experiment, ten trials with feedback were used. The smallest effect size of interest was that for the comparison between the *L*_∞_-norm (*ε* = 0.002) adversarially trained CochResNet50 and the *L*_2_-norm (*ε* = 1) waveform adversarially trained CochResNet50, yielding a partial *η*^2^ value of 0.37 for the interaction. A power analysis with G*Power indicated that 12 participants were needed to have a 95% change of seeing an effect of this size at a *P* < 0.01 significance level. To match the image experiments, we set a target of 20 participants for each main auditory experiment.

### Split-half reliability analysis of metamer confusion matrices

To assess whether human participants had consistent error patterns, we compared confusion matrices from split halves of participants. Each row of the confusion matrix (corresponding to a category label) was normalized by the number of trials for that label. We then computed the Spearman correlation between the confusion matrices from each split and compared this correlation to that obtained from confusion matrices from permuted participant responses for the condition. We computed the correlation for 1,000 random splits of participants (splitting the participants in half) and used a different permutation of the response for each split. We counted the number of times that the difference between the true split-half correlation and the shuffled correlation was less than or equal to 0 (*n*_overlap_), and the *P* value was computed as

$$\frac{1+{n}_{{{\mathrm{overlap}}}}}{1,000}.$$

### Human consistency of errors for individual stimuli

In the experiment to evaluate the consistency of errors for individual stimuli (Extended Data Fig. 7), we only included four conditions to collect enough data to analyze performance on individual images: natural images, metamers from the relu2 and final stages for the random perturbation-trained AlexNet *L*_2_-norm (*ε* = 1) model and metamers from the final stage of the adversarial perturbation-trained AlexNet *L*_2_-norm (*ε* = 1) model. The rationale for the inclusion of these stages was that the relu2 stage of the random perturbation AlexNet and the final stage of the adversarial perturbation AlexNet had similarly recognizable metamers (Fig. 4c), whereas metamers from the final stage of the random perturbation AlexNet were recognized no better than by chance by humans.

To first assess the reliability of the recognizability of individual stimuli (Extended Data Fig. 7a), we measured the Spearman correlation of the recognizability (proportion correct) of each stimulus across splits of participants separately for each of the four conditions. We averaged this correlation over 1,000 random splits of participants. *P* values were computed non-parametrically by shuffling the participant responses for each condition and each random split and computing the number of times the true average Spearman *ρ* was lower than the shuffled correlation value. We only included images in the analysis that had at least four trials in each split of participants, and when there were more than four trials in a split, we only included four of the trials, randomly selected, in the average to avoid having some images exert more influence on the result than others.

#### Most and least recognizable images

To analyze the consistency of the most and least recognizable metamers in each condition (Extended Data Fig. 7b), we used one split of participants to select 50 images that had the highest recognition score and 50 images with the lowest recognition score. We then measured the recognizability of these images in the second split of participants and assessed whether the ‘most’ recognizable images had a higher recognition score than the ‘least’ recognizable images. *P* values for this comparison were computed by using 1,000 splits of participants and measuring the proportion of splits in which the difference between the two scores was greater than 0.

To select examples of the most and least recognizable images (Extended Data Fig. 7c,d), we only included example stimuli with at least eight responses for both the natural image condition and the model metamer stage under consideration and that had 100% correct responses on the natural image condition. From this set, we selected the ‘most’ recognizable images (as those with scores of 100% correct for the considered condition) and the ‘least’ recognizable images (as those with scores of 0% correct).

### Model–brain comparison metrics for visual models

We used the Brain-Score^31^ platform to obtain metrics of neural similarity in four visual cortical areas of the macaque monkey brain: V1, V2, V4 and IT. For each model considered, we analyzed only the stages that were included in our human metamer recognition experiments. We note that some models may have had higher brain similarity scores had we analyzed all stages. Each of these model stages was fit to a public data split for each visual region, with the best-fitting stage for that region selected for further evaluation. The match of this model stage to brain data was then evaluated on a separate set of evaluation data for that region. Evaluation data for V1 consisted of the average of 23 benchmarks: 22 distribution-based comparison benchmarks from Marques et al.^89^ and the V1 partial least squares (PLS) regression benchmark from Freeman et al.^90^. Evaluation data for V2 consisted of the V2 PLS benchmark from Freeman et al.^90^. Evaluation data for V4 consisted of the average of four benchmarks: the PLS V4 benchmark from Majaj et al.^91^, the PLS V4 benchmark from Sanghavi and DiCarlo^92^, the PLS V4 benchmark from Sanghavi et al.^93^ and the PLS V4 benchmark from Sanghavi et al.^94^. Evaluation data for IT consisted of the average of four benchmarks: the PLS IT benchmark from Majaj et al.^91^, the PLS IT benchmark from Sanghavi and DiCarlo^92^, the PLS IT benchmark from Sanghavi et al.^93^ and the PLS IT benchmark from Sanghavi et al.^94^. When comparing metamer recognizability to the Brain-Score results, we used the human recognition of metamers from the model stage selected as the best match for each visual region.

We used Spearman correlations to compare metamer recognizability to the Brain-Score results. The analogous Pearson correlations were lower, and none reached statistical significance. We report Spearman correlations on the grounds that the recognizability was bounded by 0 and 1 and to be conservative with respect to our conclusion that metamer recognizability is not explained by standard model–brain comparison metrics.

We estimated the noise ceiling of the correlation between Brain-Score results and human recognizability of model metamers as the geometric mean of the reliabilities of each quantity. To estimate the reliability of the metamer recognizability, we split the participants for an experiment in half and measured the recognizability of metamers for each model stage used to obtain the Brain-Score results (that is, the best-predicting stage for each model for the brain region under consideration). We then calculated the Spearman correlation between the recognizability for the two splits and Spearman–Brown corrected to account for the 50% reduction in sample size from the split. This procedure was repeated for 1,000 random splits of participants. We then took the mean across the 1,000 splits as an estimate of the reliability. This estimated reliability was 0.917 for V1, 0.956 for V2, 0.924 for V4 and 0.97 for IT. As we did not have access to splits of the neural data used for Brain-Score, we estimated the reliability of the Brain-Score results as the Pearson correlation of the score reported in Kubilius et al.^81^ for two sets of neural responses to the same images (Spearman-Brown corrected). This estimated reliability was only available for IT (*r* = 0.87), but we assume that the reliability would be comparable for other visual areas.

### Model–brain comparison metrics for auditory models

The auditory fMRI analysis closely followed that of a previous publication^4^ using the fMRI dataset collected in another previous publication^60^. The essential components of the dataset and analysis methods are replicated here, but for additional details, see refs. ^4,60^. The text from sections fMRI data acquisition and preprocessing is an edited version of a similar section from a previous publication^4^.

#### Natural sound stimuli

The stimulus set was composed of 165 2-s natural sounds spanning 11 categories (instrumental music, music with vocals, English speech, foreign speech, non-speed vocal sounds, animal vocalization, human non-vocal sound, animal non-vocal sound, nature sound, mechanical sound or environment sound). The sounds were presented in a block design with five presentations of each 2-s sound. A single fMRI volume was collected following each sound presentation (‘sparse scanning’), resulting in a 17-s block. Silence blocks of the same duration as the stimulus blocks were used to estimate the baseline response. Participants performed a sound intensity discrimination task to increase attention. One sound in the block of five was presented 7 dB lower than the other four (the quieter sound was never the first sound), and participants were instructed to press a button when they heard the quieter sound. Sounds were presented with magnetic resonance-compatible earphones (Sensimetrics S14) at 75 dB sound pressure level (SPL) for the louder sounds and 68 dB SPL for the quieter sounds. Blocks were grouped into 11 runs, each containing 15 stimulus blocks and 4 silence blocks.

#### fMRI data acquisition and preprocessing

Data were acquired in a previous study^60^. These magnetic resonance data were collected on a 3T Siemens Trio scanner with a 32-channel head coil at the Athinoula A. Martinos Imaging Center of the McGovern Institute for Brain Research at MIT. Repetition time was 3.4 s (acquisition time was only 1 s due to sparse scanning), echo time was 30 ms, and flip angle was 90°. For each run, the five initial volumes were discarded to allow homogenization of the magnetic field. In-plane resolution was 2.1 × 2.1 mm (96 × 96 matrix), and slice thickness was 4 mm with a 10% gap, yielding a voxel size of 2.1 × 2.1 × 4.4 mm. iPAT was used to minimize acquisition time. T1-weighted anatomical images were collected in each participant (1 mm isotropic voxels) for alignment and surface reconstruction. Each functional volume consisted of 15 slices oriented parallel to the superior temporal plane, covering the portion of the temporal lobe superior to and including the superior temporal sulcus.

Functional volumes were preprocessed using FMRIB Software Library and in-house MATLAB scripts. Volumes were corrected for motion and slice time and were skull stripped. Voxel time courses were linearly detrended. Each run was aligned to the anatomical volume using FLIRT and BBRegister. These preprocessed functional volumes were then resampled to vertices on the reconstructed cortical surface computed via FreeSurfer and were smoothed on the surface with a 3-mm full-width at half-maximum two-dimensional Gaussian kernel to improve SNR. All analyses were done in this surface space, but for ease of discussion, we refer to vertices as ‘voxels’ in this paper. For each of the three scan sessions, we estimated the mean response of each voxel (in the surface space) to each stimulus block by averaging the response of the second through the fifth acquisitions after the onset of each block (the first acquisition was excluded to account for the hemodynamic lag). Pilot analyses showed similar response estimates from a more traditional general linear model^60^. These signal-averaged responses were converted to percent signal change by subtracting and dividing by each voxel’s response to the blocks of silence. These percent signal change values were then downsampled from the surface space to a 2-mm isotropic grid on the FreeSurfer-flattened cortical sheet. Analysis was performed within localized voxels in each participant.

#### fMRI data

We used the voxel responses from the original Norman-Haignere et al. study^60^, which measured fMRI responses to each natural sound relative to a silent baseline (as described in the previous section) and selected voxels with a consistent response to sounds from a large anatomical constraint region encompassing the superior temporal and posterior parietal cortex. As in Kell et al.^4^, within this set of voxels, we localized four ROIs in each participant, consisting of voxels selective for (1) frequency (that is, tonotopy), (2) pitch, (3) speech and (4) music, according to a ‘localizer’ statistical test. We excluded voxels that were selected by more than one localizer. The frequency-selective, pitch and speech localizers used additional fMRI data collected in separate scans. In total, there were 379 voxels in the frequency-selective ROI, 379 voxels in the pitch ROI, 393 voxels in the music ROI and 379 voxels in the speech ROI. The voxel responses and ROI assignments are available at https://github.com/jenellefeather/model_metamers_pytorch.

Frequency-selective voxels were identified from responses to pure tones in six different frequency ranges (center frequencies: 200, 400, 800, 1,600, 3,200 and 6,400 Hz)^95,96^ as the top 5% of all selected voxels in each participant ranked by *P* values of an ANOVA across frequency. In practice, most selected voxels centered around Heschl’s gyrus. Pitch-selective voxels were identified from responses to harmonic tones and spectrally matched noise^96^ as the top 5% of voxels in each participant with the lowest *P* values from a one-tailed *t*-test comparing those conditions. Speech-selective voxels were identified from responses to German speech and to temporally scrambled (‘quilted’) speech stimuli generated from the same German source recordings^97^. The ROI consisted of the top 5% of voxels in each participant with the lowest *P* values from a one-tailed *t*-test comparing intact and quilted speech. Music-selective voxels were identified with the music component derived by Norman-Haignere et al.^60^ as the top 5% of voxels with the most significant component weights.

#### Voxel-wise encoding analysis

We used the model responses to predict the fMRI responses. Each of the 165 sounds from the fMRI experiment was resampled to 20,000 Hz and passed through each model. To compare the model responses to the fMRI response, we averaged over the time dimension for all units that had a temporal dimension (all model stages except fully connected layers). Each voxel’s time-averaged responses were modeled as a linear combination of these responses. Ten random train–test splits (83/82) were taken from the stimulus set. For each split, we estimated a linear mapping using *L*_2_-regularized (‘ridge’) linear regression using RidgeCV from the scikit learn library version 0.23.1 (ref. ^98^). The mean response of each feature across sounds was subtracted from the regressor matrix before fitting.

The best ridge regression parameter for each voxel was independently selected using leave-one-out cross-validation across the 83 training sounds in each split sweeping over 81 logarithmically spaced values (each power of 10 between 10^−40^ and 10^40^). Holding out one sound in the training set at a time, the mean squared error of the prediction for that sound was computed using regression weights from the other 82 training set sounds for each of the regularization parameter values. The parameter value that minimized the error averaged across the held-out training sounds was used to fit a linear mapping between model responses to all 83 training set sounds and the voxel responses. This mapping was used to predict the voxel response to the 82 test sounds. Fitting fidelity was evaluated with the squared Pearson correlation (*r*^2^). Explained variance was computed for voxel responses averaged across the three scans in the original study.

This explained variance was corrected for the effects of measurement noise using the reliability of the voxel responses and the predicted voxel response^99^. Voxel response reliability (${r}_{v}^{{\prime} }$) was computed as the median Spearman–Brown-corrected Pearson correlation between all three pairs of scans, where the Spearman–Brown correction accounts for increased reliability expected from tripling the amount of data^100^. Voxel response prediction reliability (${r}_{\hat{v}}^{{\prime} }$) was similarly computed by using the training data for each of the three scans to predict the test data from the same scan and calculating the median Spearman–Brown-corrected correlation between the three pairs of predicted voxel responses. The corrected explained variance is

$${r}_{v,\hat{v}}^{2* }=\frac{{r}^{2}}{{r}_{v}^{{\prime} }{r}_{\hat{v}}^{{\prime} }},$$

where *r* is the Pearson correlation between the predicted and measured voxel responses to the test data when using the averaged voxel responses across the three scans for fitting and evaluation. If voxels and/or predictions are very unreliable, this can lead to large corrected variance explained measures^101^. We set a minimum value of 0.182 for ${r}_{v}^{{\prime} }$ (the value at which the correlation of two 83-dimensional random variables reaches significance at a threshold of *P* < 0.05; 83 being the number of training data values) and a minimum value of 0.183 for ${r}_{\hat{v}}^{{\prime} }$ (the analogous value for 82-dimensional random variables matching the number of test data values).

The corrected variance explained was computed for each voxel using each model stage for each of ten train–test splits of data. We took the median variance explained across the ten splits of data. We computed a summary metric of variance explained across each of the ROIs (Fig. 7d; all auditory voxels, tonotopic voxels, pitch voxels, music voxels and speech voxels) as follows. First, a summary measure for each participant and model stage was computed by taking the median across all voxels of the voxel-wise corrected variance explained values within the ROI. Holding out one participant, we then averaged across the remaining participant values to find the stage with the highest variance explained within the given ROI. We measured the corrected variance explained for this stage in the held-out participant. This cross-validation avoids issues of non-independence when selecting the best stage. This procedure was repeated for each participant, and we report the mean corrected variance explained across the participants. Metamer recognition was measured from the model stage most frequently chosen as the best-predicting model stage across participants (in practice, nearly all participants had the same ‘best’ model stage).

#### Noise ceiling estimates for correlation between metamer recognizability and fMRI metrics

We estimated the noise ceiling of the correlation between auditory fMRI predictivity and human recognizability of model metamers as the geometric mean of the reliabilities of each quantity. To estimate the reliability of the metamer recognizability, we split the participants for an experiment in half and measured the recognizability of metamers for the model stage that was most frequently chosen (across all participants) as the best-predicting stage for the ROI under consideration (that is, the stages used for Fig. 7d). We then calculated the Spearman correlation between the recognizability for the two splits and Spearman–Brown corrected to account for the 50% reduction in sample size from the split. This procedure was repeated for 1,000 random splits of participants. We then took the mean across the 1,000 splits as an estimate of the reliability. This estimated reliability of the metamer recognizability was 0.811, 0.829, 0.819, 0.818 and 0.801 for the best-predicting stage of all auditory voxels, the tonotopic ROI, the pitch ROI, the music ROI and the speech ROI, respectively. To estimate the reliability of the fMRI prediction metric, we took two splits of the fMRI participants and calculated the mean variance explained for each model using the stage for which recognizability was measured. We then computed the Spearman correlation between the explained variance for the two splits and Spearman–Brown corrected the result. We then repeated this procedure for 1,000 random splits of the participants in the fMRI study and took the mean across the 1,000 splits as the estimated reliability. This reliability of fMRI predictions was 0.923 for all auditory voxels, 0.768 for the tonotopic ROI, 0.922 for the pitch ROI, 0.796 for the music ROI and 0.756 for the speech ROI.

#### Representational similarity analysis

To construct the model representational dissimilarity matrix (RDM) for a model stage, we computed the dissimilarity (1 – Pearson correlation coefficient) between the model activations evoked by each pair of the 165 sounds for which we had fMRI responses. Similarly, to construct the fMRI RDM, we computed the dissimilarity in voxel responses (1 – Pearson correlation coefficient) between all ROI voxel responses from a participant to each pair of sounds. Before computing the RDMs from the fMRI or model responses, we *z* scored the voxel or unit responses.

To compute RDM similarity for the model stage that best matched an ROI (Extended Data Fig. 10), we first generated 10 random train–test splits of the 165 sound stimuli into 83 training sounds and 82 test sounds. For each split, we computed the RDMs for each model stage and for each participant’s fMRI data for the 83 training sounds. We then chose the model stage that yielded the highest Spearman *ρ* between the model stage RDM and the participant’s fMRI RDM. Using this model stage, we measured model and fMRI RDMs from the test sounds and computed the Spearman *ρ*. We repeated this procedure for each of the ten train–test splits and took the median Spearman *ρ*. We then computed the mean of this median Spearman *ρ* across participants for each model. When comparing RDM similarity to metamer recognizability, we measured recognizability from the model stage that was most frequently chosen as the best-matching model stage across participants.

As an estimate of the upper bound for the RDM correlation that could be reasonably expected to be achieved between a model RDM and a single participant’s fMRI RDM given fMRI measurement noise, we calculated the correlation between one participant’s RDM and the average of all the other participants’ RDMs. The RDMs were measured from the same ten train–test splits described in the previous paragraph using the 82 test sounds for each split. We took the median Spearman *ρ* (between RDMs) across the ten splits of data to yield a single value for each participant. The upper bound shown in Extended Data Fig. 10 is the mean across the measured value for each held-out participant. We used this upper bound rather than noise correcting the human–model RDM correlation to be consistent with prior modeling papers^102^.

### Model recognition of metamers generated from other models

To measure the recognition of a model’s metamers by other models, we took the generated image or audio that was used for the human behavioral experiments, provided it as input to a ‘recognition’ model and measured the 16-way image classification (for the visual models) or the 763-way word classification (for the auditory models).

The plots in Fig. 8b show the average recognition by other models of metamers generated from a particular type of ResNet50 model. This curve plots recognition performance averaged across all other vision recognition models (as shown in Fig. 8a). The curve for self-supervised models is also averaged across the three self-supervised generation models (SimCLR, MoCo_V2 and BYOL), and the curve for adversarially trained models is also averaged across the three adversarially trained ResNet50 models (trained with *L*_2_-norm (*ε* = 3), *L*_∞_-norm (*ε* = 4/255) and *L*_∞_-norm (*ε* = 8/255) perturbations, respectively). For these latter two curves, we first computed the average curve for each recognition model across all three generation models, omitting the recognition model from the average if it was the same as the generation model (in practice, this meant that there was one less value included in the average for the recognition models that are part of the generation model group). We then averaged across the curves for each recognition model. The error bars on the resulting curves are the s.e.m. computed across the recognition models.

The graphs in Fig. 8c,d were generated in an analogous fashion. We used one ‘standard’ generation model (the standard supervised AlexNet and CochResNet50, respectively). The curves in Fig. 8c plot results for LowPassAlexNet and VOneAlexNet. In Fig. 8d, the curve for the waveform adversarially trained models was averaged across the three such CochResNet50 models (trained with *L*_2_-norm (*ε* = 0.5), *L*_2_-norm (*ε* = 1) and *L*_∞_-norm (*ε* = 0.002) perturbations, respectively). The curve for the cochleagram adversarially trained models was averaged across the two such CochResNet50 models (trained with *L*_2_-norm (*ε* = 0.5) and *L*_2_-norm (*ε* = 1) perturbations, respectively). The group averages and error bars were computed as in Fig. 8b.

We used permutation tests to evaluate differences between the recognizability of metamers from different types of generation models and measured the statistical significance of a main effect of generation model group. We compared the observed difference between the recognizability of metamers from two generation model groups (averaged across recognition models and model stages) to a null distribution obtained from 10,000 random permutations of the generation model labels (independently permuted for each recognition model). When there was a single generation model in the group (that is, for the standard-trained model), responses were not defined for the recognition model when it was the same as the generation model. In this case, we permuted the recognition model responses as if the value existed but treated the value as missing during the average across recognition models.

### Reporting summary

Further information on research design is available in the Nature Portfolio Reporting Summary linked to this article.

## Online content

Any methods, additional references, Nature Portfolio reporting summaries, source data, extended data, supplementary information, acknowledgements, peer review information; details of author contributions and competing interests; and statements of data and code availability are available at 10.1038/s41593-023-01442-0.

### Supplementary information

Supplementary Information Supplementary Tables 1–6, Figs. 1–4, Model Notes 1 and 2 and References.

Reporting Summary

## Data Availability

Human data, trained model checkpoints and an interface to view/listen to the generated metamers used in the human recognition experiments are available at https://github.com/jenellefeather/model_metamers_pytorch. The Word–Speaker–Noise training dataset is available from the authors upon request.

## Code availability

Code for generating metamers, training models and running online experiments is available at https://github.com/jenellefeather/model_metamers_pytorch (10.5281/zenodo.8373260). Auditory front-end (cochleagram generation) code is available at https://github.com/jenellefeather/chcochleagram.
