# Supplementary material for: Model metamers reveal divergent invariances between biological and artificial neural networks
Source: Nat Neurosci. 2023 Oct 16;26(11):2017–34. doi: 10.1038/s41593-023-01442-0 (PMC10620097; doi:10.1038/s41593-023-01442-0)
Supplement: Supplementary file 1 — Supplementary Tables 1–6, Figs. 1–4, Model Notes 1 and 2 and References. [file 41593_2023_1442_MOESM1_ESM.pdf]

nature neuroscience

Article

https://doi.org/10.1038/s41593-023-01442-0

Model metamers reveal divergent invariances between biological and artificial neural networks

In the format provided by the authors and unedited

Supplementary Information

Table of Contents:

SUPPLEMENTARY TABLE 1	2
SUPPLEMENTARY FIGURE 1	3
SUPPLEMENTARY FIGURE 2	4
SUPPLEMENTARY TABLE 2	5
SUPPLEMENTARY FIGURE 3	6
SUPPLEMENTARY FIGURE 4	7
SUPPLEMENTARY MODELING NOTE 1: MODEL TRAINING, EVALUATION, AND OPTIMIZATION DETAILS	8
SUPPLEMENTARY TABLE 3	16
SUPPLEMENTARY TABLE 4	16
SUPPLEMENTARY TABLE 5	17
SUPPLEMENTARY TABLE 6	18
SUPPLEMENTARY MODELING NOTE 2: MODEL ARCHITECTURE DESCRIPTIONS	19
SUPPLEMENTARY INFORMATION REFERENCES	33

	Model	Main Effect: F-stat	Main Effect: p-value	Main Effect: effect size	Interaction: F-stat	Interaction: p-value	Interaction: effect size
Visual Models	CORnet-S	F(1,42)=424.4	$p<0.0001$	η^2_p =0.91	F(5,210)=293.6	$p<0.0001$	η^2_p =0.87
	VGG-19	F(1,42)=1612.1	$p<0.0001$	η^2_p =0.97	F(9,378)=268.4	$p<0.0001$	η^2_p =0.86
	ResNet50	F(1,42)=554.9	$p<0.0001$	η^2_p =0.93	F(7,294)=290.2	$p<0.0001$	η^2_p =0.87
	ResNet101	F(1,42)=1001.7	$p<0.0001$	η^2_p =0.96	F(7,294)=345.8	$p<0.0001$	η^2_p =0.89
	AlexNet	F(1,42)=935.4	$p<0.0001$	η^2_p =0.96	F(8,336)=195.0	$p<0.0001$	η^2_p =0.82
	ResNet50-CLIP	F(1,40)= 517.3	$p<0.0001$	η^2_p=0.93	F(6,240)=181.2	$p<0.0001$	η^2_p=0.82
	ViT-B_32-CLIP	F(1,40)= 604.5	$p<0.0001$	η^2_p=0.94	F(9,360)=110.5	$p<0.0001$	η^2_p=0.73
	ResNet50-SWSL	F(1,40)= 1159.3	$p<0.0001$	η^2_p=0.97	F(7,280)=164.7	$p<0.0001$	η^2_p=0.80
	ResNeXt101-32x3d-SWSL	F(1,40)=2363.1	$p<0.0001$	η^2_p=0.98	F(7,280)= 225.9	$p<0.0001$	η^2_p=0.85
	ViT_large_patch-16_224	F(1,40)=852.01	$p<0.0001$	η^2_p=0.96	F(8,320)=158.9	$p<0.0001$	η^2_p=0.80
Audio Models	CochCNN9	F(1,38)=551.9	$p<0.0001$	η^2_p =0.94	F(9,342)=189.2	$p<0.0001$	η^2_p =0.83
	CochResNet50	F(1,38)=467.8	$p<0.0001$	η^2_p =0.92	F(8,304)=227.4	$p<0.0001$	η^2_p =0.86

Supplementary Table 1. Human recognition of model metamers is significantly different from the generation model's recognition for all tested models, as evaluated by a main effect of observer (model or human) and an interaction between the effect of metamer generation stage and the observer.

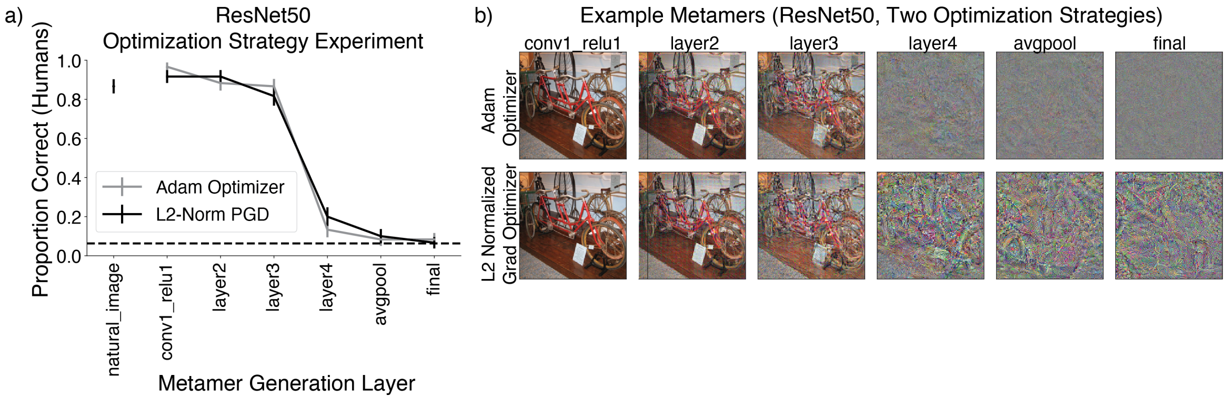

Supplementary Figure 1. Human recognition of visual model metamers generated from ResNet50 model with two different types of optimization strategies for metamer generation. An in-lab experiment was conducted to test whether differences in the optimization strategy would influence human-recognizability of model metamers. This experiment used one example visual model (a ResNet50 architecture). a) Human recognition was similar for the two optimization strategies. Error bars plot SEM across participants (N=10). b) Metamers are subjectively distinct for the two optimization methods, but not in ways that affect the recognition task. *Models and metamer generation*. The optimization code and techniques used for this experiment differed from the experiments described in the rest of this paper (they followed methods used in our previous work [1]) but showed a similar effect of model stage for standard neural network models (metamers generated from late stages of ImageNet1K task-optimized ResNet50 models were unrecognizable to humans). Models and metamer generation for this experiment were implemented in TensorFlow v1.12 [2]. We converted the ResNet50 model available via the PyTorch Model Zoo to TensorFlow using ONNX (version 1.6.0). *Optimization variants.* We tested two different optimization schemes. The first used stochastic gradient descent, where each step of gradient descent was constrained to have an L_2 norm of 1. The second method used the Adam optimizer[3], which uses an adaptive estimation of first and second order moments of the gradient, with an exponentially decaying learning rate (initial learning rate of 0.001, 1000 decay steps, and a decay rate of 0.95). In both cases optimization ran for 15000 steps. *Stimuli.* For each of the 16 categories, we randomly selected 16 examples from the ImageNet1K training dataset using the list of images provided by [4] for a total of 256 natural images that were used to generate stimuli. *Procedure.* The experiment was run together with an unrelated pilot experiment comparing four other models, the data for which are not analyzed here. To reduce the number of conditions, the stage corresponding to ResNet50 “layer1” was not included in in the experiment. Participants classified each image into one of the 16 presented categories. Each trial began with a fixation cross at the center of the screen for 300ms, followed by a natural image or a model metamer presented at the center of the screen for 200ms, followed by a pink noise mask presented for 200ms, followed by a 4x4 grid containing all 16 icons. Stimuli were presented on a 20” ACER LCD (backlit LED) monitor with a spatial resolution of 1600x900 and a refresh rate of 60Hz. Stimuli spanned 256x256 pixels and were viewed at a distance of approximately 62 cm. Before the experiment, each participant was shown a printout of the 16 category images with labels and the experimenter pointed to and read each category. This was followed by a demo experiment with 12 trials without feedback (same stimuli as in the main experiments, but performance was not used to exclude participants). Each participant saw 6 examples from each condition, chosen such that each natural image or metamer was from a unique image from the 256-image behavioral set.

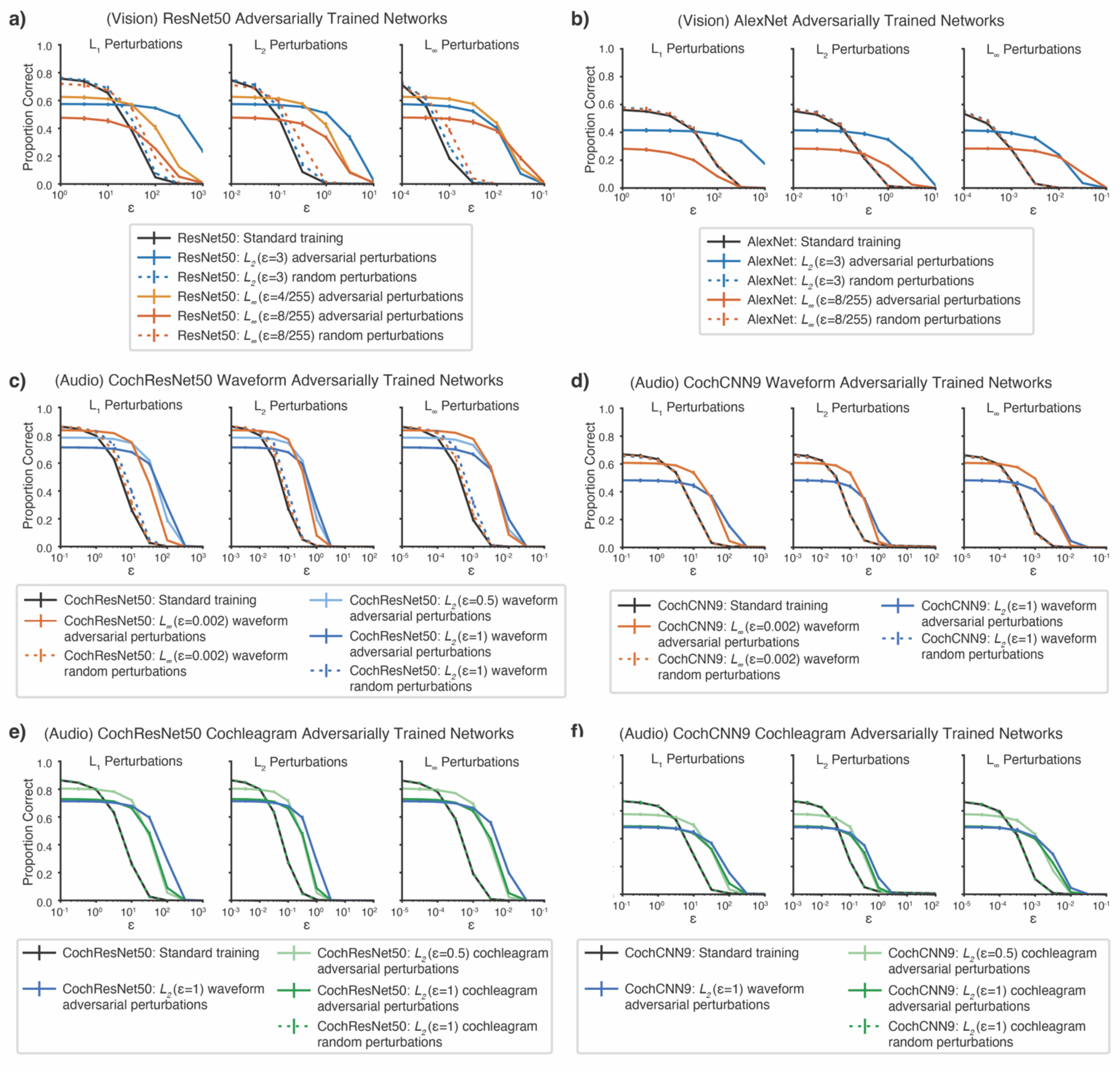

Supplementary Figure 2. Visual and auditory models become robust to adversarial attacks after adversarial training. Visual and auditory models were evaluated with L_p-norm white box adversarial attacks of varying strengths. a) ResNet50 models adversarially trained on ImageNet1K (same models and color scheme as Figure 5c,d). b) AlexNet models adversarially trained on ImageNet1K (same models and color scheme as Figure 5e,f). c) CochResNet50 models adversarially trained with waveform perturbations on word recognition (same models and color scheme as Figure 6b). d) CochCNN9 models adversarially trained with waveform perturbations on word recognition (same models and color scheme as Figure 6c). e) CochResNet50 models adversarially trained with cochleagram perturbations on word recognition (same models and color scheme as Figure 6f). f) CochCNN9 models adversarially trained with cochleagram perturbations on word recognition (same models and color scheme as Figure 6g). For each model and perturbation type, performance was evaluated for five random subsets of 1024 examples from the validation set. Error bars are SEM across the 5 subsets. For auditory models, adversarial perturbations for evaluation were always added to the waveform (because cochleagram perturbations are not necessarily realizable as audio signals due to overcompleteness). Adversarial training produced robustness to adversarial perturbations (better performance at large perturbation sizes), along with some reduction in clean accuracy, as is typical for adversarially trained models[5]. Training with random perturbations typically produced similar results as standard training, as expected. In many plots, the results for random-perturbation training (dotted lines) overlap with those for standard training (black line).

	Model 1	Model 2	F-stat	p-value	effect size
Visual Models	ResNet50 L_2-norm ($\epsilon = 3$)	ResNet50 Standard Supervised	$F(1,19)=792.3$	$p<0.0001$	$\eta^2_p=0.98$
	ResNet50 L_2-norm ($\epsilon = 3$)	ResNet50 random L_2-norm ($\epsilon = 3$)	$F(1,19)=242.2$	$p<0.0001$	$\eta^2_p=0.93$
	ResNet50 L_∞-norm ($\epsilon = 8/255$)	ResNet50 Standard Supervised	$F(1,19)=315.3$	$p<0.0001$	$\eta^2_p=0.94$
	ResNet50 L_∞-norm ($\epsilon = 8/255$)	ResNet50 random L_∞-norm ($\epsilon = 8/255$)	$F(1,19)=176.3$	$p<0.0001$	$\eta^2_p=0.90$
	ResNet50 L_∞-norm ($\epsilon = 4/255$)	ResNet50 Standard Supervised	$F(1,19)=403.0$	$p<0.0001$	$\eta^2_p=0.95$
	AlexNet L_2-norm ($\epsilon = 3$)	AlexNet Standard Supervised	$F(1,19)=518.6$	$p<0.0001$	$\eta^2_p=0.96$
	AlexNet L_2-norm ($\epsilon = 3$)	AlexNet random L_2-norm ($\epsilon = 3$)	$F(1,19)=623.0$	$p<0.0001$	$\eta^2_p=0.97$
	AlexNet L_∞-norm ($\epsilon = 8/255$)	AlexNet Standard Supervised	$F(1,19)=104.6$	$p<0.0001$	$\eta^2_p=0.85$
	AlexNet L_∞-norm ($\epsilon = 8/255$)	AlexNet random L_∞-norm ($\epsilon = 8/255$)	$F(1,19)=121.4$	$p<0.0001$	$\eta^2_p=0.86$
Audio Models, Waveform Perturbations	CochCNN9 waveform L_2-norm ($\epsilon = 1$)	CochCNN9 Standard	$F(1,19)=210.3$	$p<0.0001$	$\eta^2_p=0.92$
	CochCNN9 waveform L_2-norm ($\epsilon = 1$)	CochCNN9 random waveform L_2-norm ($\epsilon = 1$)	$F(1,19)=107.3$	$p<0.0001$	$\eta^2_p=0.85$
	CochCNN9 waveform L_∞-norm ($\epsilon = 0.002$)	CochCNN9 Standard	$F(1,19)=133.4$	$p<0.0001$	$\eta^2_p=0.88$
	CochCNN9 waveform L_∞-norm ($\epsilon = 0.002$)	CochCNN9 random waveform L_∞-norm ($\epsilon = 0.002$)	$F(1,19)=114.6$	$p<0.0001$	$\eta^2_p=0.86$
	CochResNet50 waveform L_2-norm ($\epsilon = 0.5$)	CochResNet50 Standard	$F(1,19)=9.77$	$p=0.0067$	$\eta^2_p=0.34$
	CochResNet50 waveform L_2-norm ($\epsilon = 1$)	CochResNet50 Standard	$F(1,19)=0.29$	$p=0.59$	$\eta^2_p=0.015$
	CochResNet50 waveform L_2-norm ($\epsilon = 1$)	CochResNet50 random waveform L_2-norm ($\epsilon = 1$)	$F(1,19)=8.6$	$p=0.0097$	$\eta^2_p=0.31$
	CochResNet50 waveform L_∞-norm ($\epsilon = 0.002$)	CochResNet50 random waveform L_∞-norm ($\epsilon = 0.002$)	$F(1,19)=4.8$	$p=0.044$	$\eta^2_p=0.20$
	CochResNet50 waveform L_∞-norm ($\epsilon = 0.002$)	CochResNet50 Standard	$F(1,19)=9.26$	$p=0.007$	$\eta^2_p=0.33$
Audio Models, Cochleagram Perturbations	CochCNN9 cochleagram L_2-norm ($\epsilon = 0.5$)	CochResNet50 Standard	$F(1,19)=166.3$	$p<0.0001$	$\eta^2_p=0.90$
	CochCNN9 cochleagram L_2-norm ($\epsilon = 1$)	CochResNet50 Standard	$F(1,19)=145.2$	$p<0.0001$	$\eta^2_p=0.88$
	CochCNN9 cochleagram L_2-norm ($\epsilon = 1$)	CochCNN9 random cochleagram L_2-norm ($\epsilon = 1$)	$F(1,19)=157.2$	$p<0.0001$	$\eta^2_p=0.89$
	CochCNN9 cochleagram L_2-norm ($\epsilon = 1$)	CochCNN9 waveform L_2-norm ($\epsilon = 1$)	$F(1,19)=12.6$	$p=0.0023$	$\eta^2_p=0.40$
	CochResNet50 cochleagram L_2-norm ($\epsilon = 0.5$)	CochResNet50 Standard	$F(1,19)=102.2$	$p<0.0001$	$\eta^2_p=0.84$
	CochResNet50 cochleagram L_2-norm ($\epsilon = 1$)	CochResNet50 Standard	$F(1,19)=145.1$	$p<0.0001$	$\eta^2_p=0.88$
	CochResNet50 cochleagram L_2-norm ($\epsilon = 1$)	CochResNet50 random cochleagram L_2-norm ($\epsilon = 1$)	$F(1,19)=127.4$	$p<0.0001$	$\eta^2_p=0.87$
	CochResNet50 cochleagram L_2-norm ($\epsilon = 1$)	CochResNet50 waveform L_2-norm ($\epsilon = 1$)	$F(1,19)=66.6$	$p<0.0001$	$\eta^2_p=0.78$

Supplementary Table 2. Metamers for adversarial trained networks are more human-recognizable than metamers from standard networks or networks with random perturbations. Each comparison is evaluated with a repeated measure ANOVA comparing human recognition of Model 1 to Model 2. In audio models, statistical tests were also performed between models with different locations of adversarial perturbations (waveform or cochleagram perturbations).

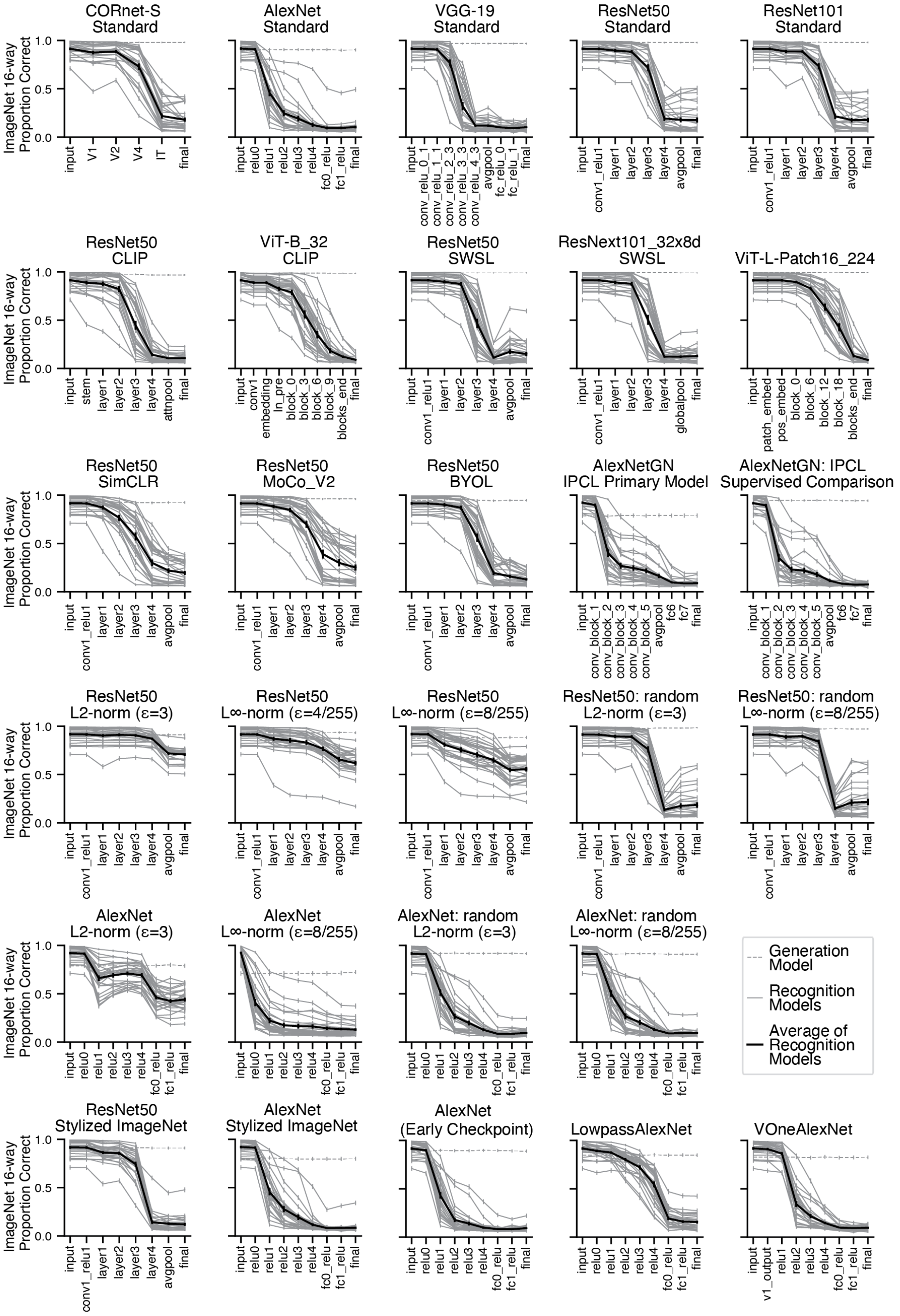

Supplementary Figure 3. Image model recognition of metamers generated from other models. Metamers were generated from the model indicated in the plot title and recognition was measured by presenting the metamers to other models (each grey line on the plot corresponds to one recognition model). Error bars on individual model curves are bootstrapped (1000 bootstraps) SEM from the model predictions. Error bars on the average recognition curve is the SEM across recognition models (N=28 recognition models). Model metamers from deep stages tend to be unrecognizable to other models, but models trained with adversarial perturbations or with architecturally fixed lowpass filtering operations have metamers that are more recognizable by other models.

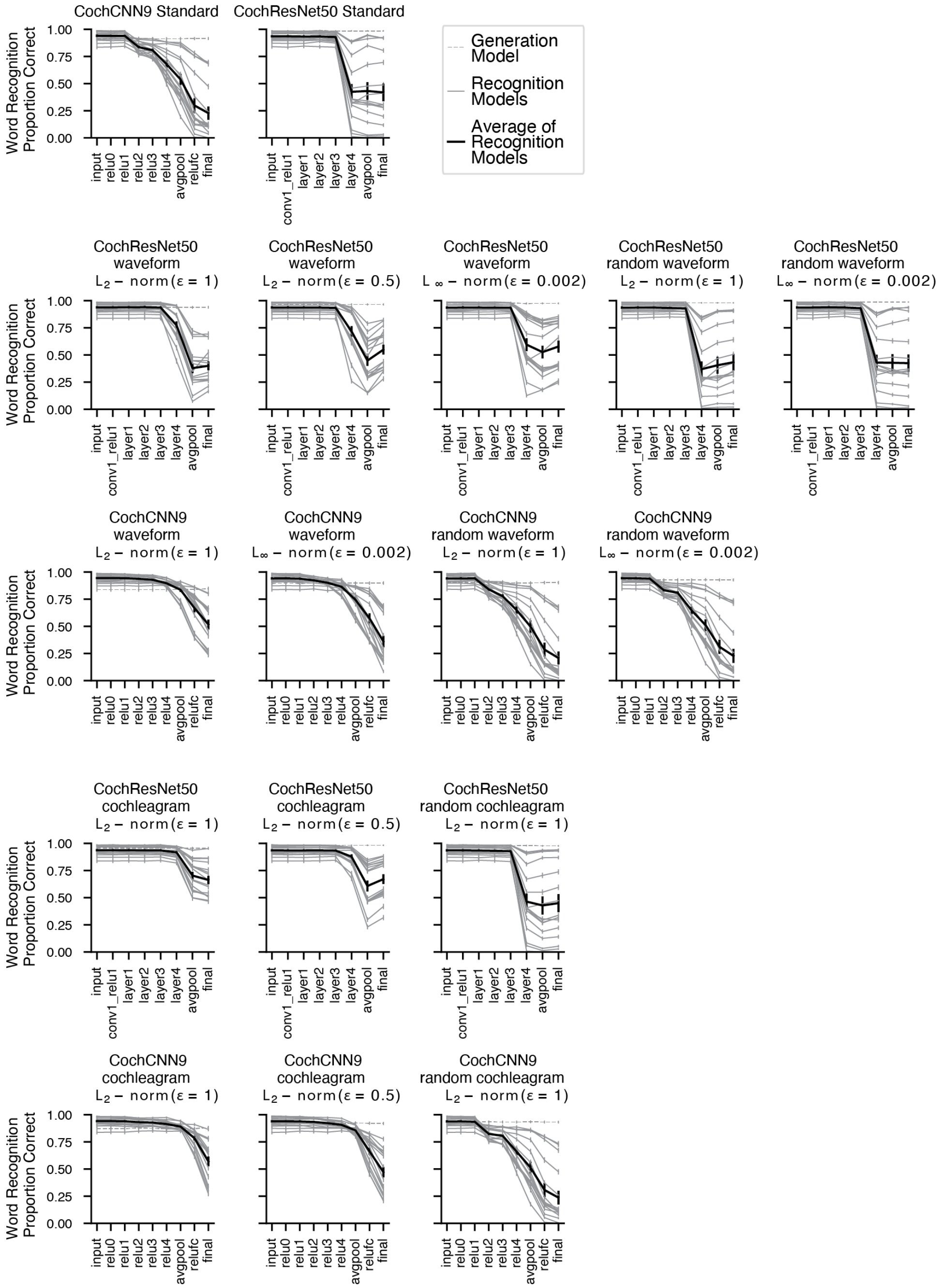

Supplementary Figure 4. Auditory model recognition of metamers generated from other models. Metamers were generated from the model indicated in the plot title and recognition was measured by presenting the metamers to other models (each grey line on the plot corresponds to one recognition model). Error bars on individual model curves are bootstrapped (1000 bootstraps) SEM from the model predictions. Error bars on the average recognition curve is the SEM across recognition models (N=16 recognition models). As with the image models, model metamers from deep stages tend to be unrecognizable to other models, but models trained with adversarial perturbations have metamers that are more recognizable by other models.

Supplementary Modeling Note 1: Model training, evaluation, and optimization details

Models were trained and evaluated with the PyTorch deep learning library [6], and the Robustness library [7], modified to accommodate metamer generation and auditory model training. Model code and instructions for downloading checkpoints are available online at https://github.com/jenellefeather/model_metamers_pytorch. Model architecture descriptions are provided in Supplementary Modeling Note 2. All models were trained on the OpenMind computing cluster at MIT using NVIDIA GPUs with a minimum of 11GB memory. In the methods sections that follow we often refer to model stages as "layers", to be consistent with how they are named in PyTorch. "Stage" and "layer" should be taken as synonymous.

Image training dataset

Unless otherwise noted, all visual neural network models were trained on the ImageNet1K Large Scale Visual Recognition Challenge dataset [8]. This classification task consists of 1000 classes of images with 1,281,167 images in the training set and 50,000 images in the validation set. All classes were used for training the neural network models. Accuracy on ImageNet1K task and additional training parameters are reported in Supplementary Table 3.

ImageNet1K model training and evaluation

Unless otherwise described below, visual models trained on ImageNet1K consisted of publicly available checkpoints. Standard supervised models used the pretrained PyTorch checkpoints from torchvision.models (documentation https://pytorch.org/vision/stable/models.html, referred to as "pytorch" in Supplementary Table 3). The input pixel values ranged from 0-1 (or from 0-255 in the case of HMAX). Visual model performance was evaluated as the model accuracy on the ImageNet1K validation set, implemented by resizing the images so the smallest dimension was 256 pixels (or 250 in the case of HMAX) and taking a center crop of 224x224 (or 250 in the case of HMAX) pixels of the image. Train, test, and metamer images were all normalized by subtracting channel means and dividing by channel standard deviations before being passed into the first stage of the neural network backbone (except in the case of HMAX, where this normalization was not applied). Channel means were set to [0.485, 0.456, 0.406] and channel standard deviations were set to [0.229, 0.224, 0.225] unless otherwise noted for the architecture. ImageNet1K model training used data-parallelization to split batches across multiple GPUs.

Visual models trained on large-scale datasets

We also tested five visual models that were pretrained on datasets larger than the ImageNet1K dataset described above. Two of these were the visual encoders from Contrastive Language-Image Pre-Training (CLIP) models (one with a ResNet50 visual encoder and one with a ViT-B_32 visual encoder), obtained from the publicly available checkpoints at https://github.com/openai/CLIP (referred to as "clip" in Supplementary Table 3) [9]. CLIP models were trained on a dataset of 400 million (image, text) pairs collected from the internet. ImageNet1K performance from the CLIP models is evaluated with a zero-shot prediction using the list of prepared 80 image template prompts and modified ImageNet labels from [9]. We found empirically that we could not synthesize metamers from this classifier, and so only included model stages from the visual encoder in our experiments. CLIP models used a custom channel mean of [0.48145466, 0.4578275, 0.40821073] and a channel standard deviation of [0.26862954, 0.26130258, 0.27577711]. The third and fourth of these models were Semi-Weakly Supervised (SWSL) ImageNet models (ResNet50 and ResNeXt101-32x8d architectures), obtained from the publicly available checkpoints at https://github.com/facebookresearch/semi-supervised-ImageNet1K-models (referred to as "swsl" in Supplementary Table 3) [10]. SWSL Models are pre-trained on 940 million public images with 1.5K hashtags matching with the 1000 synsets in the ImageNet dataset, followed by fine-tuning on the ImageNet1K training images described above. The fifth model was a Vision Transformer (ViT_large_patch-16_224, [11]), obtained from the publicly available checkpoint at https://github.com/rwightman/pytorch-image-models (referred to as "timm" in Supplementary Table 1). ViT_large_patch-16_224 was pre-trained on the ImageNet-21K dataset, consisting of approximately 14 million images with about 21000 distinct object categories, and then fine-tuned on the ImageNet1K training images described above.

Self-supervised ResNet50 vision models

Self-supervised ResNet50 models were downloaded from the OpenSelfSup Model Zoo, and the training details that follow are taken from the documentation (https://github.com/open-mmlab/OpenSelfSup, referred to as "openselfsup" in Supplementary Table 3). Three models, each with a ResNet50 architecture, were used: MoCo_V2, SimCLR and BYOL. MoCo_V2 self-supervised training had a batch size of 256, with data augmentations consisting of random crop (224x224 pixels), random horizontal flip (probability=0.5), random color jitter (brightness=0.4, contrast=0.4, saturation=0.4, hue=0.1, probability=0.8, all values uniformly chosen), random greyscale (probability=0.2), and random gaussian blur (sigma_min=0.1, sigma_max=0.2, probability=0.5). SimCLR self-supervised training had a batch size of 256, with augmentations consisting of random crop (224x224 pixels), random

horizontal flip (probability=0.5), random color jitter (brightness=0.8, contrast=0.8, saturation=0.8, hue=0.2, probability=0.8, all values uniformly chosen), random greyscale (probability=0.2), and random gaussian blur (sigma_min=0.1, sigma_max=0.2, probability=0.5). BYOL self-supervised training had a batch size of 4096, with augmentations consisting of random crop (224x224 pixels), random horizontal flip (probability=0.5), random color jitter (brightness=0.4, contrast=0.4, saturation=0.2, hue=0.1, probability=0.8, all values uniformly chosen), random greyscale (probability=0.2), and random gaussian blur (sigma_min=0.1, sigma_max=0.2, probability=0.5). For all self-supervised ResNet50 models, a linear readout consisting of a fully connected layer with 1000 units applied to the average pooling layer of the model was trained using the same augmentations used for supervised training of the other ImageNet1K-trained models described above. The linear readout was trained for 100 epochs of ImageNet1K (while the model backbone up to avgpool remained unchanged). For MoCo_V2 and SimCLR models, the accuracy was within 1% of that reported on the OpenSelfSup (BYOL average pooling evaluation was not posted at the time of training). The linear readout served as a check that the downloaded models were instantiated correctly, and was used to help verify the success of the metamer generation optimization procedure, as described below. Linear evaluations from each model stage were obtained by training a fully connected layer with 1000 units and a softmax classifier applied to a random subsample of 2048 activations. If the number of activations was less than or equal to 2048 all activations were maintained.

Self-supervised IPCL AlexNetGN models

A model trained with Instance-Prototype Contrastive Learning (IPCL) and a supervised model with the same augmentations were downloaded from the IPCL github (https://github.com/harvard-visionlab/open_ipcl, referred to as “ipcl" in Supplementary Table 3) [12]. Both models used an AlexNet architecture with group-normalization layers. As described in the original publication [12], the models were trained with a batch size of 128x5 (128 images with 5 augmentations each), for 100 epochs, with data augmentations consisting of a random resize crop (random crop of the image resized with a scale range of [0.2,1] and aspect ratio [3/4,4/3], and resized to 224x224 pixels), random horizontal flip (probability=0.5), random grayscale conversion (probability=0.2), random color jitter (brightness=0.6, contrast=1, saturation=0.4, and hue +/- 144 degrees).

Using the procedure described in [12], a linear readout consisting of a fully connected layer with 1000 units was used to evaluate each model stage. The readout was trained using a batch size of 256, with input augmentations of a random resize crop (random crop of the image resized with a scale range of [0.08,1] and aspect ratio [3/4,4/3], and resized to 224x224 pixels) and a random horizontal flip (probability=0.5). The linear readout was trained for 10 epochs with the one-cycle learning rate policy [13], with cosine annealing to vary the learning rate from 0.00003, increasing to a maximum of .3 after 3 epochs, then decreasing with a cosine annealing function toward zero (3e-09) by 10 epochs.

Models trained on Stylized ImageNet

Models trained on a “Stylized” ImageNet were downloaded from publicly available checkpoints (https://github.com/rgeirhos/texture-vs-shape, referred to as “texture-vs-shape” in Supplementary Table 3) and training details that follow are taken from the documentation [14]. Stylized ImageNet is constructed by taking the content of an ImageNet1K image and replacing the style of the image with that of a randomly selected painting using AdaIN style transfer [15]. A single stylized version of each image in the ImageNet1K training dataset was used for training. The models were trained with a batch size of 256 for 60 epochs, with input augmentations of a random resize crop (random crop of the image resized with a scale range of [0.08,1] and aspect ratio [3/4,4/3], and resized to 224x224 pixels) and a random horizontal flip (probability=0.5).

HMAX vision model

The hand-engineered HMAX vision model was based off of a publicly availably implementation in PyTorch (https://github.com/wmvanvliet/pytorch_hmax) which follows the model documented in a previous publication [16]. A gaussian activation function was used, and boundary handling was added to match the MATLAB implementation provided by the original HMAX authors (https://maxlab.neuro.georgetown.edu/hmax.html). For full comparison to the other models, we trained a linear classifier consisting of 1000 units to perform the ImageNet1K recognition task on the final C2 output of the HMAX model. This fully connected layer was trained for 30 epochs of the ImageNet1K training dataset, and the learning rate was dropped after every 10 epochs. Inputs to HMAX during the classifier training consisted of random crops (250x250 pixels), random horizontal flip (p=0.5), random color jitter (brightness=0.1, contrast=0.1, saturation=0.1, probability=1, all values uniformly chosen), and lighting noise (alpha standard deviation of 0.05, an eigenvalue of [0.2175, 0.0188, 0.0045], and channel eigenvectors of [[-0.5675, 0.7192, 0.4009], [-0.5808, -0.0045, -0.8140], [-0.5836, -0.6948, 0.4203]]). HMAX performance was evaluated by measuring the model accuracy on the ImageNet1K validation set after resizing the images so that the smallest dimension was 250 pixels, taking a center crop of 250x250 pixels of the image, converting to greyscale, and

multiplying by 255 to scale the image to the 0-255 range. As expected, the performance on this classifier was low, but it was significantly above chance and could thus be used for the metamer optimization criteria described below.

Empirically, we found that both of the hand-engineered models contained stages that were difficult to optimize with the same strategy used for the neural networks. In both cases we found that optimization was aided by selectively optimizing for subsets of the units (channels) in the early iterations of the optimization process. For the HMAX model, the subsets that were chosen depended on the model stage. For the S1 stage, we randomly choose activations from a single Gabor filter channel to include in the optimization. For the C1 stage, we randomly selected a single scale. And for the S2 and C2 stages we randomly chose a single patch size. The random choice of subset was changed after every 50 gradient steps. This subset-based optimization strategy was used for the first 2000 iterations at each learning rate value. All units were then included for the remaining 1000 iterations for that learning rate value. Unlike the other models in this paper, we used only the Signal-To-Noise Ratio for the matching criterion, because we found empirically that after the S2 stage of the HMAX model, activations from pairs of random images became strongly correlated due to the different offsets and scales in the natural image patch set, such that the correlation measures were not diagnostic of the match fidelity.

Adversarial training – vision models

Adversarially trained ResNet50 models were obtained from the robustness library (https://github.com/MadryLab/robustness, referred to as "robustness" in Supplementary Table 3). Adversarially trained AlexNet architectures and the random perturbation ResNet50 and AlexNet architectures were trained for 120 epochs of the ImageNet1K dataset, with image pixel values scaled between 0-1, using data parallelism to split batches across multiple GPUs. Learning rate was decreased by a factor of 10 after every 50 epochs of training. During training, data augmentation consisted of random crop (224x224 pixels), random horizontal flip (probability=0.5), color jitter (brightness=0.1, contrast=0.1, saturation=0.1, probability=1, all values uniformly chosen), and lighting noise (alpha standard deviation of 0.05, an eigenvalue of [0.2175, 0.0188, 0.0045], and channel eigenvectors of [[-0.5675, 0.7192, 0.4009], [-0.5808, -0.0045, -0.8140], [-0.5836, -0.6948, 0.4203]]). An adversarial or random perturbation was then added. All adversarial examples were untargeted, such that the loss used to generate the adversarial example pushed the input away from the original class by maximizing the cross-entropy loss, but did not push the prediction towards a specific target class. For the L_2-norm ($\epsilon = 3$) model, adversarial examples were generated with a step size of 1.5 and 7 attack steps. For the L_∞-norm ($\epsilon = 8/255$) model, adversarial examples were generated with a step size of 4/255 and 7 attack steps. For both ResNet50 and AlexNet random-perturbation L_2-norm models, a random sample on the L_2 ball with width $\epsilon = 3$ was drawn and added to the input, independently for each training example and dataset epoch. Similarly, for both Resnet50 and AlexNet random perturbation L_∞-norm models, a random sample on the corners of the L_∞ ball was selected by randomly choosing a value of ±8/255 to add to each image pixel, independently chosen for each training example and dataset epoch. After the adversarial or random perturbation was added to the input image, the new image was clipped between 0-1 before being passed into the model.

VOneAlexNet vision model

The VOneAlexNet architecture was downloaded from the VOneNet GitHub repository (https://github.com/dicarlolab/vonenet) [17]. Modifications were then made to use Gaussian noise rather than Poisson noise as the stochastic component, as in [18], and to use the same input normalization as in our other models (rather than a mean of 0.5 and standard deviation of 0.5 as used in [17]). The VOneAlexNet architecture was trained for 120 epochs using the same data augmentations and training procedure described for the adversarially trained AlexNet model (but without adversarial or random pertubrations). The model was trained with stochastic responses (Gaussian noise with standard deviation of 4) in the "VOne" model stage, but for the purposes of metamer generation we fixed the noise by randomly drawing one noise sample when loading the model and using this noise sample for all metamer generation and adversarial evaluation. Although "fixing" the noise reduces the measured adversarial robustness compared to when a different sample of noise is used for each iteration of the adversarial example generation, the model with a single noise draw was still significantly more robust than a standard model, and allowed us to perform the metamer experiments without having to account for the stochastic representation during metamer optimization.

LowpassAlexNet vision model

The LowpassAlexNet architecture was trained for 120 epochs using the same augmentations and training procedure described for the adversarially trained AlexNet models (but without adversarial or random perturbations). To approximately equate performance on natural stimuli with the VOneNetAlexNet, we chose an early checkpoint that was closest, but did not exceed, the Top 1% performance of the VOneAlexNet model (to ensure that the greater

recognizability of the metamers from LowpassAlexNet could not be explained by higher overall performance of that model). This resulted in a comparison model trained for 39 epochs of the ImageNet1K dataset.

AlexNet vision model, early checkpoint
We trained an AlexNet architecture for 120 epochs using the same augmentations and training procedure described for the adversarially trained AlexNet models (but without adversarial or random perturbations). After training, to approximately equate performance on natural stimuli with the VOneNetAlexNet and LowpassAlexNet, we chose an early checkpoint that was closest, but not lower than, the performance of the VOneAlexNet model. This resulted in a comparison model trained for 51 epochs of the ImageNet1K dataset.

Pre-trained adversarially robust models for final stage evaluation
To compare the relationship between metamer recognizability and adversarial robustness of adversarially trained models (Figure 6c), we evaluated a large set of adversarially trained models. We evaluated all of the models included in a well-known robustness evaluation as of November 2022 – these comprised the ImageNet- L_∞ evaluation of robustbench [19] (8 models), as well as additional models from each of the repositories from which these models were chosen (17 additional models), for a total of 25 models. Five of these models were from https://github.com/dedeswim/vits-robustness-torch [20], and were trained with L_∞-norm, $\epsilon = 4/255$, (ViT-XCiT-L12, ViT-XCiT-M12, ViT-XCit-S12, ConvNeXt-T, and GELUResNet-50). Another 16 of these models were from https://github.com/microsoft/robust-models-transfer [21], with 3 trained with L_∞-norm, $\epsilon = 4/255$, (ResNet18, ResNet50, and Wide-ResNet-50-2), 3 trained with L_∞-norm, $\epsilon = 1/255$, (ResNet18, ResNet50, and Wide-ResNet-50-2), and 10 trained with L_2-norm, $\epsilon = 3.0$, (ResNet18, ResNet50, Wide-ResNet-50-2, Wide-ResNet-50-4, DenseNet, MNASNET, MobileNet-v2, ResNeXt50_32x4d, ShuffleNet, VGG16_bn). Another 3 models were ResNet50 architectures from https://github.com/MadryLab/robustness [7]; one was trained on L_∞-norm, $\epsilon = 4/255$, one was trained on L_∞-norm, $\epsilon = 8/255$, and was one trained on L_2-norm, $\epsilon = 3.0$). Lastly, 1 model was the ResNet50 checkpoint available from https://github.com/locuslab/fast_adversarial [22]. Only the final layer was used for metamer generation for these models. These models are omitted from Supplementary Table 3 as they were used for only a single analysis (that of Figure 6b).

Adversarial evaluation -- visual models
The adversarial robustness of visual models was evaluated with white-box untargeted adversarial attacks (i.e., in which the attacker has access to the model's parameters when determining an attack that will cause the model to classify the image as any category other than the correct one). All 1000 classes of ImageNet1K were used for the adversarial evaluation. Attacks were computed with L_1, L_2, *and* L_∞ maximum perturbation sizes (ε) added to the image, with 64 gradient steps each with size ε/4 (pilot experiments suggested that this step size and number of steps were sufficient to produce adversarial examples for most models). We randomly chose images from the ImageNet1K evaluation dataset to use for adversarial evaluation, applying the evaluation augmentation described above (resizing so that the smallest dimension was 256 pixels, followed by a center crop of 224x224 pixels). Five different subsets of 1024 stimuli were drawn to compute error bars.

For the detailed investigation of adversarial vulnerability shown in Supplemental Figure 9, we measured robustness to two additional types of white-box adversarial attacks. "Fooling Images" [23] were constructed by first initializing the input image as a sample from a normal distribution with standard deviation of 0.05 and a mean of 0.5. We then randomly chose a target label from the 1000 classes of ImageNet1K and derived a perturbation to the image that would cause the noise to be classified as the target class. Performance was evaluated as the percent of perturbed images that had the target label. Attacks were computed with L_1, L_2, *and* L_∞ maximum perturbation sizes (ε) added to the image, with 64 gradient steps each with size ε/4. Error bars were computed using five different random samples of 1024 target labels. "Feature Adversaries" [24] were constructed by deriving small perturbations to a natural "source" image to yield model activations (at a particular model stage) that are close to those evoked by a different natural "target" image, by minimizing the L_2 distance between the perturbed source image activations and the target activations. The source and target images were randomly selected from the ImageNet1K validation dataset. Evaluation was performed by measuring the percent of perturbed images that had the same label as the target image. Attacks were computed with L_1, L_2, *and* L_∞ maximum perturbation sizes (ε) added to the image, with 128 gradient steps each with size ε/16. Error bars were computed using five different subsets of 1024 "source" and "target" stimuli.

For the statistical comparisons between the adversarial robustness of architectures for Figure 6f we performed a repeated measure ANOVA with within-group factors of architecture and perturbation size ϵ. A separate ANOVA was performed for each adversarial attack type. The values of ϵ included in the ANOVA were constrained to a range

where the VOneAlexNet and LowPassAlexNet showed robustness over the standard AlexNet (four values for each attack type, $\epsilon_{L_1} \in \{10^{1.5}, 10^2, 10^{2.5}, 10^3\}$, $\epsilon_{L_2} \in \{10^{-1}, 10^{-0.5}, 10^0, 10^{0.5}\}$, $\epsilon_{L_\infty} \in \{10^{-3.5}, 10^{-3}, 10^{-2.5}, 10^{-2}\}$), so that any difference in clean performance did not affect the comparisons. We computed statistical significance for the main effect of architecture by a permutation test, randomly permuting the architecture assignment, independent for each subset of the data. We computed a p-value by comparing the observed F-statistic to the null distribution of F-statistics from permuted data (i.e., the p-value was one minus the rank of the observed F-statistic divided by the number of permutations). In cases where the maximum possible number of unique permutations was less than 10,000, we instead divided the rank by the maximum number of unique permutations.

We performed the same type of ANOVA analysis for the statistical comparisons in Supplementary Figure 9, using adversarial attack strengths for which VOneAlexNet and LowPassAlexNet showed robustness over the standard AlexNet for the specific attack being evaluated. For the "Fooling Images" in Supplementary Figure 9a we used attack strengths of $\epsilon_{L_1} \in \{10^{1.5}, 10^2, 10^{2.5}, 10^3\}$, $\epsilon_{L_2} \in \{10^{-1}, 10^{-0.5}, 10^0, 10^{0.5}\}$, $\epsilon_{L_\infty} \in \{10^{-3.5}, 10^{-3}, 10^{-2.5}, 10^{-2}\}$, and for the feature adversaries in Supplementary Figure 9b we used attack strengths of $\epsilon_{L_1} \in \{10^{2.5}, 10^3, 10^{3.5}, 10^4\}$, $\epsilon_{L_2} \in \{10^0, 10^{0.5}, 10^1, 10^{1.5}\}$, $\epsilon_{L_\infty} \in \{10^{-2.5}, 10^{-2}, 10^{-1.5}, 10^{-1}\}$).

Out of distribution evaluation – visual models

We evaluated model performance on out of distribution images using two publically available benchmarks. For both benchmarks, we utilized the BrainScore [25] implementations of the behavioral benchmarks for the models.

The ImageNet-C benchmark [26] measures model top 1 accuracy on distorted images derived from the ImageNet test set, using the labels for the original image. The accuracy is averaged over stimulus sets consisting of the following distortions: Gaussian noise, shot noise, impulse noise, defocus blur, frosted glass blur, motion blur, zoom blur, snow, frost, fog, brightness, contrast, elastic, pixelate, and JPEG compression.

The Geirhos 2021 benchmark [27] measures whether the model gets the same stimuli correct as human observers (error consistency), using 16-way recognition decisions from human observers for comparison. The error consistency is averaged over stimulus sets consisting of the following stimulus manipulations: colour/grayscale, constrast, high-pass, low-pass (blurr), phase scrambling, power equalization, false colour, rotation, Eidolon I, Eidolon II, Eidonlon III, uniform noise, sketch, stylized, edge, silhouette, and texture-shape cue conflict.

Audio training dataset

All auditory neural network models were trained on the Word-Speaker-Noise (WSN) dataset. This dataset was first presented in [1] and was constructed from existing speech recognition and environmental sound classification datasets. The dataset is approximately balanced to enable performance of three tasks on the same training exemplar: (1) recognition of the word at the center of a two second speech clip (2) recognition of the speaker and (3) recognition of environmental sounds, that are superimposed with the speech clips (serving as "background noise" for the speech tasks while enabling an environmental sound recognition task). Although the dataset is constructed to enable all three tasks, the models described in this paper were only trained to perform the word recognition task. The speech clips used in the dataset were excerpted from the Wall Street Journal [28] (WSJ) and Spoken Wikipedia [29] (SWC).

To choose speech clips, we screened WSJ, TIMIT [30] and a subset of articles from SWC for appropriate audio clips (specifically, clips that contained a word at least four characters long and that had one second of audio before the beginning of the word and after the end of the word, to enable the temporal jittering augmentation described below). Some SWC articles were left out of the screen due to a) potentially offensive content for human listening experiments; (29/1340 clips), b) missing data; (35/1340 clips), or c) bad audio quality (for example, due to computer generated voices of speakers reading the article or the talker changing mid-way through the clip; 33/1340 clips). Each segment was assigned the word class label of the word overlapping the segment midpoint and a speaker class label determined by the speaker. With the goal of constructing a dataset with speaker and word class labels that were approximately independent, we selected words and speaker classes such that the exemplars from each class spanned at least 50 unique cross-class labels (e.g., 50 unique speakers for each of the word classes). This exclusion fully removed TIMIT from the training dataset. We then selected words and speaker classes that each contained at least 200 unique utterances, and such that each class could contain a maximum of 25% of a single cross-class label (e.g., for a given word class, a maximum of 25% of utterances could come from the same speaker). These exemplars were subsampled so that the maximum number in any word or speaker class was less than 2000. The resulting training dataset contained 230,356 unique clips in 793 word classes and 432 speaker classes, with

40,650 unique clips in the test set. Each word class had between 200 and 2000 unique exemplars. A "null" class was used as a label when a background clip was presented without the added speech.

The environmental soundtrack clips that were superimposed on the speech clips were a subset of examples from the AudioSet dataset (a set of annotated YouTube video soundtracks) [31]. To minimize ambiguity for the two speech tasks, we removed any sounds under the "Speech" or "Whispering" branch of the AudioSet ontology. Since a high proportion of AudioSet clips contain music, we achieved a more balanced set by excluding any clips that were only labeled with the root label of "Music", with no specific branch labels. We also removed silent clips by first discarding everything tagged with a "Silence" label and then culling clips containing more than 10% zeros. This screening resulted in a training set of 718,625 unique natural sound clips spanning 516 categories. Each AudioSet clip was a maximum of 10 seconds long, from which a 2-second excerpt was randomly cropped during training (see below).

Auditory model training

During training, the speech clips from the Word-Speaker-Noise dataset were randomly cropped in time and superimposed on random crops of the AudioSet clips. Data augmentations during training consisted of 1) randomly selecting a clip from AudioSet to pair with each labeled speech clip, 2) randomly cropping 2 seconds of the AudioSet clip and 2 seconds of the speech clip, cropped such that the labeled word remained in the center of the clip (due to training pipeline technicalities, we used a pre-selected set of 5,810,600 paired speech and natural sound crops which spanned 25 epochs of the full set of speech clips and 8 passes through the full set of AudioSet clips), 3) superimposing the speech and the noise (i.e., the AudioSet crop) with a Signal-to-Noise-Ratio (SNR) sampled from a uniform distribution between -10dB SNR and 10dB SNR, augmented with additional samples of speech without an AudioSet background (i.e. with infinite SNR, 2464 examples in each epoch) and samples of AudioSet without speech (i.e. with negative infinite SNR, 2068 examples in each epoch) and 4) setting the root-mean-square (RMS) amplitude of the resulting signal to 0.1. Evaluation performance is reported on one pass through the speech test set (i.e., one crop from each of the 40,650 unique test set speech clips) constructed with the same augmentations used during training (specifically, variable SNR and temporal crops, paired with a separate set of AudioSet test clips, same random seed used to test each model such that test sets were identical across models). Audio model training used data-parallelization to split batches across multiple GPUs.

Each auditory model was trained for 150 epochs (where an epoch is defined as a full pass through the set of 230,356 speech training clips). The learning rate was decreased by a factor of 10 after every 50 epochs (see Supplementary Table 4).

Auditory model cochlear stage

The first stage of the auditory models produced a "cochleagram" – a time-frequency representation of audio with frequency tuning that mimics the human ear, followed by a compressive nonlinearity [32]. This stage consisted of the following sequence of operations. First, the 20kHz audio waveform passed through a bank of 211 bandpass filters with center frequencies ranging from 50Hz to 10kHz. Filters were zero-phase with frequency response equal to the positive portion of a single period of a cosine function, implemented via multiplication in the frequency domain. Filter spacing was set by the Equivalent Rectangular Bandwidth (ERB_N) scale [33]. Filters perfectly tiled the spectrum such that the summed squared response across all frequencies was flat (four low-pass and four high-pass filters were included in addition to the bandpass filters in order to achieve this perfect tiling). Second, the envelope was extracted from each filter subband using the magnitude of the analytic signal (via the Hilbert transform). Third, the envelopes were raised to the power of 0.3 to simulate basilar membrane compression. Fourth, the compressed envelopes were lowpass-filtered and downsampled to 200Hz (1d convolution with a Kaiser-windowed Sinc filter of size 1001 in the time domain, applied with a stride of 100 and no zero padding, i.e. "valid" convolution), resulting in a final "cochleagram" representation of 211 frequency channels by 390 time points. The first stage of the neural network "backbone" of the auditory models operated on this cochleagram representation. Cochleagram generation was implemented in PyTorch such that the components were differentiable for metamer generation and adversarial training. Cochleagram generation code will be released upon acceptance of the paper.

Spectemp model

The hand-engineered Spectro-Temporal filter model (Spectemp) was based on a previously published model [34]. Our implementation differed from the original model in specifying spectral filters in cycles/ERB rather than cycles/octave (because our implementation operated on a cochleagram generated with ERB-spaced filters). The model consisted of a linear filter bank tuned to spectro-temporal modulations at different frequencies, spectral scales, and temporal rates. The filtering was implemented via 2D convolution with zero padding in frequency (211 samples) and time (800 samples). Spectro-temporal filters were constructed with spectral modulation center frequencies of [0.0625, 0.125, 0.25, 0.5, 1, 2] cycles/ERB and temporal modulation center frequencies of [0.5, 1, 2,

4, 8, 16, 32, 64] Hz, including both upward and downward frequency modulations (resulting in 96 filters). An additional 6 purely spectral and 8 purely temporal modulation filters were included for a total of 110 modulation filters. This filterbank operated on the cochleagram representation (yielding the 'filtered_signal' stage in Figure 4d-f). We squared the output of each filter response at each time step ('power') and took the average across time for each frequency channel ('average'), similar to previous studies [35–37]. To be able to use model classification judgments as part of the metamer generation optimization criteria (see below), we trained a linear classifier after the average pooling layer (trained for 150 epochs of the speech training set with a learning rate that started at 0.01 and decreased by a factor of 10 after every 50 speech epochs, using the same data augmentations as for the neural networks). Although performance on the word recognition task for the Spectemp model was low, it was significantly above chance, and thus could be used to help verify the success of the metamer generation optimization procedure.

For the Spectemp model, we observed that the higher frequency modulation channels were hardest to optimize. We set up a coarse-to-fine optimization strategy by initially only including the lowest frequency spectral and temporal modulation filters in the loss function, and adding in the filters with the next lowest modulation frequencies after every 400 optimization steps (with 7 total sets of filters defined by center frequencies in both temporal and spectral modulation, and the remaining 200/3000 steps continuing to include all of the filters from the optimization). The temporal modulation cutoffs for each of the 7 sets were [0, 0.5, 1, 2, 4, 8, 16] Hz and the spectral modulation cutoffs were [0, 0.0625, 0.125, 0.25, 0.5, 1, 2] cycles/ERB; a filter was included in the nth set if it had either a temporal or spectral scale that was equal to or less than the nth temporal or spectral cutoff, respectively. This strategy was repeated for each learning rate.

Adversarial training – auditory models – waveform perturbations

CochResNet50 and CochCNN9 were adversarially trained with perturbations in the waveform domain. We also included a control training condition in which random perturbations were added to the waveform. For both adversarial and random waveform perturbations, after the perturbation was added, the audio signal was clipped to fall between -1 and 1. As with the adversarially trained vision models, all adversarial examples were untargeted. The L_2-norm ($\epsilon = 0.5$ and $\epsilon = 1.0$) model adversarial examples were generated with a step size of 0.25 and 0.5, respectively, and 5 attack steps. L_∞-norm ($\epsilon = 0.002$) model adversarial examples in the waveform space were generated with a step size of 0.001 and 5 attack steps. For random perturbation L_2-norm models (both CochResNet50 and CochCNN9), a random sample on the L_2 ball with width $\epsilon = 1.0$ was selected and added to the waveform, independently for each training example and dataset epoch. Similarly, for random perturbation L_∞-norm models, a random sample on the corners of the L_∞ ball was selected by randomly choosing a value of ±0.002 to add to each image pixel, chosen independently for each training example and dataset epoch.

We estimated the $\mathrm{SNR_{dB}}$ for the perturbations of the waveform using:

$$\mathrm{SNR_{dB}} = 20\log_{10}\frac{||x||}{||\xi||}$$

where x is the input waveform and ξ is the adversarial perturbation. As described above, the input waveforms, x, to the model were RMS normalized to 0.1, and thus $||x|| = 0.1 * \sqrt{n}$, where n is the number of samples in the waveform (40,000). For L_2-norm perturbations to the waveform, the norm of the perturbation is just the ϵ value, and so $\epsilon = 0.5$ and $\epsilon = 1.0$ correspond to $||\xi|| = 0.5$ and $||\xi|| = 1$, resulting in $\mathrm{SNR_{dB}}$ values of 32.04 and 26.02, respectively. For L_∞-norm perturbations, the worst case (lowest) $\mathrm{SNR_{dB}}$ is achieved by a perturbation that maximally changes every input value. Thus, an L_∞ perturbation with $\epsilon = 0.002$ has $||\xi|| = \sqrt{\sum_{n=1}^{40,000}(0.002^2)}$, corresponding to a $\mathrm{SNR_{dB}}$ value of 33.98. These $\mathrm{SNR_{dB}}$ values do not guarantee that the perturbations were always fully inaudible to humans, but they confirm that the perturbations are relatively minor and unlikely to be salient to a human listener.

Adversarial training – auditory models – cochleagram perturbations

CochResNet50 and CochCNN9 were adversarially trained with perturbations in the cochleagram domain. The fixed components of the cochleagram generation enabled norm-based constraints on the perturbation size to the cochleagram analogous to those used for input-based adversarial examples. Although the perturbation size on the cochleagram is not directly comparable to the perturbation size on the waveform, in each case we chose the perturbation size during adversarial training to be large enough that the model showed robustness to adversarial perturbations while not being so large that the model could not perform the task (as is standard for adversarial training). Further, training models with perturbations generated at the cochleagram stage resulted in substantial robustness to adversarial examples generated at the waveform (Supplementary Figure 2). We also included a control training condition in which random perturbations were added to the cochleagram. Adversarial or random perturbations were added to the output of the cochleagram stage, after which the signal was passed through a

ReLU so that no negative values were fed into the neural network backbone. All adversarial examples were untargeted. The L_2-norm ($\epsilon = 0.5$ and $\epsilon = 1.0$) model adversarial examples were generated with a step size of 0.25 and 0.5 respectively, and 5 attack steps. For random perturbation L_2-norm models (both CochResNet50 and CochCNN9), a random sample on the L_2 ball with width $\epsilon = 1.0$ was selected, independently for each training example and dataset epoch.

We estimated the $\mathrm{SNR_{dB}}$ of the cochleagram perturbations using the average cochleagram from the test dataset, whose L_2-norm was 40.65. Using this value with the $\mathrm{SNR_{dB}}$ equation yielded estimates of 38.20 and 32.18 dB for cochleagram perturbation models trained with $\epsilon = 0.5$ and $\epsilon = 1.0$, respectively. We again cannot guarantee that the perturbations are inaudible to a human, but they are fairly low in amplitude and thus unlikely to be salient.

Adversarial evaluation – auditory models

As in visual adversarial evaluation, the adversarial vulnerability of auditory models was evaluated with untargeted white-box adversarial attacks. Attacks were computed with L_1, L_2, and L_∞ maximum perturbation sizes (ε) added to the waveform, with 64 gradient steps each with size ε/4 (pilot experiments and previous results [18] suggested that this step size and number of steps were sufficient to attack most auditory models). We randomly chose audio samples from the WSN evaluation dataset to use for adversarial evaluation, including the evaluation augmentations described above (additive background noise augmentation with SNR randomly chosen between -10 to 10 dB SNR, and RMS normalization to 0.1). Five different subsets of 1024 stimuli were drawn to compute error bars.

Comparison of auditory adversarial robustness to metamer recognizability

When comparing adversarial robustness to model metamer recognition (Figure 6b), the model metamer recognizability was evaluated using intermediate stage metamers (layer4 for CochResNet50, and ReLU4 for CochCNN9). This was because the final stage metamer recognizability was sufficiently low overall (because the auditory recognition task was much harder than the visual task: 793 vs. 16 possible classes) that a floor effect plausibly explained the absence of a significant correlation for the final stage metamers (ρ=0.21, p=0.215).

Model	Learning Rate (at Start)	Batch Size	Accuracy (Top 1)	Accuracy (Top 5)
AlexNet Standard	(pretrained, pytorch)	(pretrained)	56.518	79.070
AlexNet L_2-norm ($\epsilon = 3$)	0.01	256	41.882	65.018
AlexNet random L_2-norm ($\epsilon = 3$)	0.01	256	57.978	79.986
AlexNet L_∞-norm ($\epsilon = 8/255$)	0.01	256	29.702	51.034
AlexNet random L_∞-norm ($\epsilon = 8/255$)	0.01	256	57.738	79.996
ResNet50 Standard	(pretrained, pytorch)	(pretrained)	76.130	92.862
ResNet50 L_2-norm ($\epsilon = 3$)	(pretrained, robustness)	(pretrained)	57.900	80.706
ResNet50 random L_2-norm ($\epsilon = 3$)	0.1	256	76.600	93.136
ResNet50 L_∞-norm ($\epsilon = 4/255$)	(pretrained, robustness)	(pretrained)	62.424	84.060
ResNet50 L_∞-norm ($\epsilon = 8/255$)	(pretrained, robustness)	(pretrained)	47.906	72.484
ResNet50 random L_∞-norm ($\epsilon = 8/255$)	0.1	256	73.062	91.366
ResNet101 Standard	(pretrained, pytorch)	(pretrained)	77.374	93.546
VGG-19 Standard	(pretrained, pytorch)	(pretrained)	72.376	90.876
CORnet-S Standard	(pretrained, cornet)	(pretrained)	73.020	91.116
ResNet50-BYOL	(pretrained, openselfsup) / 30 linear eval	(pretrained) / 256 linear eval	68.706	88.090
ResNet50-MOCO_V2	(pretrained, openselfsup) / 30 linear eval	(pretrained) / 256 linear eval	67.832	88.322
ResNet50-SIMCLR	(pretrained, openselfsup) / 30 linear eval	(pretrained / 256 linear eval	59.204	81.304
HMAX	0.1 linear eval	256	6.101	15.070
VOneAlexNet	0.01	256	47.84	71.57
LowpassAlexNet	0.01	256	47.620	72.416
AlexNet (EarlyCheckpoint)	0.01	256	52.536	76.446
CLIP-ResNet50	(pretrained, clip) / zero-shot eval	(pretrained)	59.822	86.568
CLIP-ViT-B-32	(pretrained, clip) / zero-shot eval	(pretrained)	63.360	88.820
SWSL-ResNet50	(pretrained, swsl)	(pretrained)	81.180	95.986
SWSL-ResNeXt50	(pretrained, swsl)	(pretrained)	84.294	97.174
ViT_large_patch-16_224	(pretrained, timm)	(pretrained)	84.374	97.232
AlexNetGN IPCL Primary Model	(pretrained, ipcl) / cosine annealing (0.00003 to 0.03)	(pretrained) / 256 linear eval	40.288	63.622
AlexNetGN IPCL Supervised Comparison	(pretrained, ipcl)	(pretrained)	60.988	82.786
AlexNet Stylized ImageNet Trained	(pretrained, texture-vs-shape)	(pretrained)	40.012	64.178
ResNet50 Stylized ImageNet Trained	(pretrained, texture-vs-shape)	(pretrained)	60.184	82.616

Supplementary Table 3: Vision architecture training parameters and ImageNet1K accuracy.

Model	Learning Rate (at Start)	Batch Size	Accuracy (Top 1)	Accuracy (Top 5)
CochCNN9 Standard	0.01	128	66.672	83.129
CochCNN9 waveform L_2-norm ($\epsilon = 1$)	0.01	128	48.091	67.240
CochCNN9 random waveform L_2-norm ($\epsilon = 1$)	0.01	128	65.710	82.376
CochCNN9 waveform L_∞-norm ($\epsilon = 0.002$)	0.01	128	60.440	78.278
CochCNN9 random waveform L_∞-norm ($\epsilon = 0.002$)	0.01	128	66.283	82.952
CochCNN9 cochleagram L_2-norm ($\epsilon = 1$)	0.01	128	48.002	66.089
CochCNN9 cochleagram L_2-norm ($\epsilon = 0.5$)	0.01	128	57.198	75.001
CochCNN9 random cochleagram L_2-norm ($\epsilon = 1$)	0.01	128	66.706	83.087
CochResNet50 Standard	0.1	256	86.797	95.360
CochResNet50 waveform L_2-norm ($\epsilon = 0.5$)	0.1	256	78.130	90.536
CochResNet50 waveform L_2-norm ($\epsilon = 1$)	0.1	256	70.546	85.474
CochResNet50 random waveform L_2-norm ($\epsilon = 1$)	0.1	256	85.916	94.871
CochResNet50 waveform L_∞-norm ($\epsilon = 0.002$)	0.1	256	83.491	93.658
CochResNet50 random waveform L_∞-norm ($\epsilon = 0.002$)	0.1	256	86.367	95.090
CochResNet50 cochleagram L_2-norm ($\epsilon = 1$)	0.1	256	71.392	85.149
CochResNet50 cochleagram L_2-norm ($\epsilon = 0.5$)	0.1	256	80.435	91.444
CochResNet50 random cochleagram L_2-norm ($\epsilon = 1$)	0.1	256	86.556	95.144
Spectemp (linear eval)	0.01 (linear eval)	128 (linear eval)	5.743	13.780

Supplementary Table 4: Auditory model architecture training parameters and word classification accuracy.

Experiment	Models	Total Number of conditions (Includes Natural Image)	Number of trials per condition per participant Average (min, max)	Number of Participants
Visual Experiment 1 (Standard Models)	CORnet-S<br>VGG-19<br>ResNet50<br>ResNet101<br>AlexNet	37	10 (8, 14)	22
Visual Experiment 2 (Large-Scale Dataset Models)	ResNet50: CLIP<br>ViT-B_32: CLIP<br>ResNet50: SWSL<br>ResNeXt101-32_8d: SWSL<br>ViT_large_patch-16_224	39	10 (7,13)	21
Visual Experiment 3 (Self-Supervised ResNet50 Models)	ResNet50 Standard Supervised<br>ResNet50 SimCLR<br>ResNet50 MoCo_V2<br>ResNe50 BYOL	29	13 (11, 20)	21
Visual Experiment 4 (IPCL AlexNetGN)	IPCL Primary Model<br>IPCL Supervised Comparison	19	21 (18, 24)	23
Visual Experiment 5 (Stylized-ImageNet Trained Models)	ResNet50 Standard ImageNet Trained<br>ResNet50 Stylized ImageNet Trained<br>AlexNet Standard ImageNet Trained<br>AlexNet Stylized ImageNet Trained	31	12 (11, 16)	21
Visual Experiment 6 (HMAX)	HMAX	6	33 (31, 34)	20
Visual Experiment 7 (ResNet50 Adversarially Robust)	ResNet50 Standard Supervised<br>ResNet50 L_2-norm ($\epsilon = 3$)<br>ResNet50 random L_2-norm ($\epsilon = 3$)<br>ResNet50 L_∞-norm ($\epsilon = 4/255$)<br>ResNet50 L_∞-norm ($\epsilon = 8/255$)<br>ResNet50 random L_∞-norm ($\epsilon = 8/255$)	43	9 (6, 13)	20
Visual Experiment 8 (AlexNet Adversarially Robust)	AlexNet Standard Supervised<br>AlexNet L_2-norm ($\epsilon = 3$)<br>AlexNet random L_2-norm ($\epsilon = 3$)<br>AlexNet L_∞-norm ($\epsilon = 8/255$)<br>AlexNet random L_∞-norm ($\epsilon = 8/255$)	41	9 (7, 14)	20
Visual Experiment 9 (AlexNet with Regularized Metamers)	AlexNet Standard with No Regularization<br>AlexNet Standard with Regularization (Small Step)<br>AlexNet Standard with Regularization (Large Step)<br>AlexNet Adversarially Trained L_2-norm ($\epsilon = 3$)	33	12 (8, 18)	20
Visual Experiment 10 (Single Image Consistency Experiment)	Natural Image<br>AlexNet random perturbation L_2-norm ($\epsilon = 3$) – ReLU2<br>AlexNet random perturbation L_2-norm ($\epsilon = 3$) – Final Stage<br>AlexNet adversarial perturbation L_2-norm ($\epsilon = 3$) – Final Stage	4	100 (96, 103)	40
Visual Experiment 11 (Adversarially Trained Models Final Stage)	Adversarially Trained Models (see Methods)	27	14 (12, 17)	21
Visual Experiment 12 (Lowpass AlexNet and VOneAlexNet)	AlexNet Standard Supervised<br>Lowpass AlexNet<br>VOneAlexNet	25	16 (14, 20)	20

Supplementary Table 5. Conditions and number of trials included in each visual experiment. Each condition was initially allocated ceiling(400/N) trials, and then trials were removed at random until the total number of trials was equal to 400. In addition, if the stimulus for a condition did not pass the metamer optimization criteria (and thus, had to be omitted from the experiment), the natural image was substituted for it as a placeholder, and analyzed as an additional trial for the natural condition. These two constraints resulted in the number of trials per condition varying somewhat across. The HMAX experiment was run with 200 rather than 400 because it contained only 6 conditions (metamer optimization was run for all 400 stimuli and a subset of 200 images was randomly chosen from the subset of the 400 images for which metamer optimization was successful in every stage of the model). HMAX metamers were black and white, while all metamers from all other models were in color.

Experiment	Models	Total Number of conditions (Includes Natural Audio)	Number of trials per condition per participant Average (min, max)	Number of Participants
Auditory Experiment 1 (Standard Models)	CochResNet50 Standard<br>CochCNN9 Standard	18	22 (18, 23)	20
Auditory Experiment 2 (Spectemp Model)	Spectemp Model	6	33 (30, 34)	20
Auditory Experiment 3 (CochResNet50 Waveform Adversarial Training)	CochResNet50 Standard Supervised<br>CochResNet50 waveform L_2-norm ($\epsilon = 0.5$)<br>CochResNet50 waveform L_2-norm ($\epsilon = 1$)<br>CochResNet50 random waveform L_2-norm ($\epsilon = 1$)<br>CochResNet50 waveform L_∞-norm ($\epsilon = 0.002$)<br>CochResNet50 random waveform L_∞-norm ($\epsilon = 0.002$)	49	8 (5, 9)	20
Auditory Experiment 4 (CochCNN9 Waveform Adversarial Training)	CochCNN9 Standard Supervised<br>CochCNN9 waveform L_2-norm ($\epsilon = 1$)<br>CochCNN9 random waveform L_2-norm ($\epsilon = 1$)<br>CochCNN9 waveform L_∞-norm ($\epsilon = 0.002$)<br>CochCNN9 random waveform L_∞-norm ($\epsilon = 0.002$)	46	8 (6, 9)	20
Auditory Experiment 5 (CochResNet50 Cochleagram Adversarial Training)	CochResNet50 Standard Supervised<br>CochResNet50 cochleagram L_2-norm ($\epsilon = 0.5$)<br>CochResNet50 cochleagram L_2-norm ($\epsilon = 1$)<br>CochResNet50 random cochleagram L_2-norm ($\epsilon = 1$)<br>CochResNet50 waveform L_2-norm ($\epsilon = 1$)	41	9 (7, 10)	20
Auditory Experiment 6 (CochCNN9 Cochleagram Adversarial Training)	CochCNN9 Standard Supervised<br>CochCNN9 cochleagram L_2-norm ($\epsilon = 0.5$)<br>CochCNN9 cochleagram L_2-norm ($\epsilon = 1$)<br>CochCNN9 random cochleagram L_2-norm ($\epsilon = 1$)<br>CochCNN9 waveform L_2-norm ($\epsilon = 1$)	46	8 (6, 9)	20

Supplementary Table 6. Conditions and number of trials included in each auditory experiment As in the visual experiments, each condition was initially allocated ceiling(400/N) trials, and then trials were removed at random until the total number of trials was equal to 400. If the model stage selected produced a metamer that did not pass the metamer optimization criteria (and thus, was omitted from experiment stimuli), the natural audio was used instead, but was not included in the analysis. As in the visual experiments, these two constraints resulted in the number of trials per condition varying somewhat across participants. The Spectemp experiment used only 200 of the original 400 excerpts, for a total of 216 trials. This experiment was run with a smaller number of stimuli because it contained only 6 conditions (metamer optimization was run for all 400 stimuli and a subset of 200 was randomly chosen from the subset of the 400 original excerpts for which metamer optimization was successful in every stage of the model).

Supplementary Modeling Note 2: Model Architecture Descriptions

The general structure of the neural network architectures used in the paper are documented in this file, including names for the model stages that were used for metamer generation and included on figures. Model stage names and number of features are only given for the model stages used in metamer experiments (bolded in the tables below). All output shapes are specified without a batch dimension.

CORnet-S

The CORnet-S architecture was proposed in [38] and contains recurrent and skip connections motivated by brain and behavioral data.

Model Stage Name	PyTorch Operation	Output Shape	Number of Features
**natural_image**	**input**	**(3,224,224)**	**150528**
	Conv2d(3, 64, kernel_size=7, stride=2, padding=3, bias=False)	(64, 112, 112)	
	BatchNorm2d(64)	(64, 112, 112)	
	ReLU	(64, 112, 112)	
	MaxPool2d(kernel_size=3, stride=2, padding=1)	(64, 56, 56)	
	Conv2d(64, 64, kernel_size=3, stride=1, padding=1, bias=False)	(64, 56, 56)	
	BatchNorm2d(64)	(64, 56, 56)	
**V1**	**ReLU**	**(64, 56, 56)**	**200704**
	Conv2d(64, 128, kernel_size=1, stride=1, bias=False)	(128, 56, 56)	
**V2**	**CORblock_S(128, scale=4, time=2)**	**(128, 28, 28)**	**100352**
	Conv2d(128, 256, kernel_size=1, stride=1, bias=False)	(256, 28, 28)	
**V4**	**CORblock_S(256, scale=4, time=4)**	**(256, 14, 14)**	**50176**
	Conv2d(256, 512, kernel_size=1, stride=1, bias=False)	(512, 14, 14)	
**IT**	**CORblock_S(512, scale=4, time=2)**	**(512, 7, 7)**	**25088**
	AdaptiveAvgPool2d(1)	(512, 1, 1)	
**final**	**Linear(512, 1000)**	**(1000)**	**1000**

The CORblock_S(channels, scale, t) components of the architecture have the following structure:

1.	Input (x)
2.	Conv2d(channels, channels * scale, kernel_size=1, bias=False)
3.	BatchNorm2d(channels * scale)
4.	ReLU
5.	t=0: Conv2d(channels * scale, channels * scale, kernel_size=3, stride=2, padding=1, bias=False)<br>t != 0: Conv2d(channels * scale, channels * scale, kernel_size=3, stride=1, padding=1, bias=False)
6.	BatchNorm2d(channels * scale)
7.	ReLU
8.	Conv2d(channels * scale, channels, kernel_size=1, bias=False)
9.	BatchNorm2d(channels)

10.	Process skip connection (on input, x):<br>t=0: x processed with<br>a. 1x1 Conv2d(channels, channels, kernel_size=1, stride=2, bias=False)<br>b. BatchNorm2d(channels)<br><br>t != 0: x processed with Identity()
11.	Add output from (9) to output from (10)
12.	(Output) ReLU

To implement recurrent connections, the input passes through this CORblock_S block `t` times, where the convolutional layers share weights for each timestep `t` but the batch normalization layers have unique learnable weights for each `t`. The first pass `t=0` through the block contains additional downsampling of the residual connection and in the second convolution.

VGG19

The VGG19 architecture was proposed in [39] and has 16 convolutional layers, 5 max pooling layers, and 2 fully connected layers (plus one classification layer).

Model Stage Name	PyTorch Operation	Output Shape	Number of Features
**natural_image**	**input**	**(3,224,224)**	**150528**
	Conv2d(3, 64, kernel_size=3, padding=1)	(64, 224, 224)	
	ReLU	(64, 224, 224)	
	Conv2d(64, 64, kernel_size=3, padding=1)	(64, 224, 224)	
**conv_relu_0_1**	**ReLU**	**(64, 224, 224)**	**3211264**
	MaxPool2d(kernel_size=2, stride=2)	(64, 112, 112)	
	Conv2d(64, 128, kernel_size=3, padding=1)	(128, 112, 112)	
	ReLU	(128, 112, 112)	
	Conv2d(128, 128, kernel_size=3, padding=1)	(128, 112, 112)	
**conv_relu_1_1**	**ReLU**	**(128, 112, 112)**	**1605632**
	MaxPool2d(kernel_size=2, stride=2)	(128, 56, 56)	
	Conv2d(128, 256, kernel_size=3, padding=1)	(256, 56, 56)	
	ReLU	(256, 56, 56)	
	Conv2d(256, 256, kernel_size=3, padding=1)	(256, 56, 56)	
	ReLU	(256, 56, 56)	
	Conv2d(256, 256, kernel_size=3, padding=1)	(256, 56, 56)	
	ReLU	(256, 56, 56)	
	Conv2d(256, 256, kernel_size=3, padding=1)	(256, 56, 56)	
**conv_relu_2_3**	**ReLU**	**(256, 56, 56)**	**802816**
	MaxPool2d(kernel_size=2, stride=2)	(256, 28, 28)	
	Conv2d(256, 512, kernel_size=3, padding=1)	(512, 28, 28)	
	ReLU	(512, 28, 28)	
	Conv2d(512, 512, kernel_size=3, padding=1)	(512, 28, 28)	
	ReLU	(512, 28, 28)	

	Conv2d(512, 512, kernel_size=3, padding=1)	(512, 28, 28)	
	ReLU	(512, 28, 28)	
	Conv2d(512, 512, kernel_size=3, padding=1)	(512, 28, 28)	
**conv_relu_3_3**	**ReLU**	**(512, 28, 28)**	**401408**
	MaxPool2d(kernel_size=2, stride=2)	(512, 14, 14)	
	Conv2d(512, 512, kernel_size=3, padding=1)	(512, 14, 14)	
	ReLU	(512, 14, 14)	
	Conv2d(512, 512, kernel_size=3, padding=1)	(512, 14, 14)	
	ReLU	(512, 14, 14)	
	Conv2d(512, 512, kernel_size=3, padding=1)	(512, 14, 14)	
	ReLU	(512, 14, 14)	
	Conv2d(512, 512, kernel_size=3, padding=1)	(512, 14, 14)	
**conv_relu_4_3**	**ReLU**	**(512, 14, 14)**	**100352**
	MaxPool2d(kernel_size=2, stride=2)	(512, 7, 7)	
**avgpool**	**AdaptiveAvgPool2d((7, 7)) (note: this is equal to the output of the above MaxPool2d)**	**(512, 7, 7)**	**25088**
	Linear(512 * 7 * 7, 4096)	(4096)	
**fc_relu_0**	**ReLU**	**(4096)**	**4096**
	Dropout(p=0.5)	(4096)	
	Linear(4096, 4096)	(4096)	
**fc_relu_1**	**ReLU**	**(4096)**	**4096**
	Dropout(p=0.5)	(4096)	
**final**	**Linear(4096, 1000)**	**(1000)**	**1000**

ResNet50

The ResNet50 architecture was proposed in [40] and has 48 convolutional layers with 1 max pooling layer and 1 average pooling layer (plus one classification layer). It has 4 residual blocks (ResNetBlocks below) which have skip (or shortcut) connections. This architecture is used for all models labled as "ResNet50" with the exception of ResNet50: CLIP, which has modifications described below.

Layer names and number of features are only given for the layers used in metamer experiments.

Model Stage Name	PyTorch Operation	Output Shape	Number of Features
**natural_image**	**input**	**(3,224,224)**	**150528**
	Conv2d(3, 64, kernel_size=7, stride=2, padding=3, bias=False)	(64, 112, 112)	
	BatchNorm2d(64)	(64, 112, 112)	
**conv1_relu1**	**ReLU**	**(64, 112, 112)**	**802816**
	MaxPool2d(kernel_size=3, stride=2, padding=1)	(64, 56, 56)	
**layer1**	**ResNetBlock(inplanes=64, planes=64, num_blocks=3, stride=1)**	**(256, 56, 56)**	**802816**

**layer2**	**ResNetBlock(inplanes=256, planes=128, num_blocks=4, stride=2)**	**(512, 28, 28)**	**401408**
**layer3**	**ResNetBlock(inplanes=512, planes=256, num_blocks=6, stride=2)**	**(1024, 14, 14)**	**200704**
**layer4**	**ResNetBlock(inplanes=1024, planes=512, num_blocks=3, stride=2)**	**(2048, 7, 7)**	**100352**
**avgpool**	**AdaptiveAvgPool2d(1,1)**	**(2048, 1, 1)**	**2048**
**final**	**Linear(2048, 1000)**	**(1000)**	**1000**

The ResNetBlock components of the architecture have the following structure:

1. input (x)
2. 1x1 Conv2d(inplanes, planes, stride=1)
3. BatchNorm2d(planes)
4. ReLU
5. 3x3 Conv2d (planes, planes, stride=1)
6. BatchNorm2d(planes)
7. ReLU
8. 1x1 Conv2d (planes, planes * expansion, stride=1)
9. BatchNorm2d(planes)
10. Residual connection on x (if inplanes !=planes * expansion): 1x1 Conv2D (inplanes, planes * expansion, stride)
11. Residual connection on x (if inplanes !=planes * expansion): BatchNorm2d(planes * expansion)
12. Add output from (9) to output from (11)
13. (Output) ReLU

Multiple of these residual blocks (num_blocks) are stacked together to form a single ResNetBlock. The expansion factor was set to four for all layers (expansion=4).

ResNet50: CLIP modifications

The CLIP model with a ResNet50 visual encoder obtained from https://github.com/openai/CLIP had three "stem" convolutions as opposed to one, with an average pool instead of a max pool. It also prepends an avgpool to convolutions with stride > 1 within the residual blocks, and uses a final attention pooling layer rather than average pooling. Full architecture details below.

Layer names and number of features are only given for the layers used in metamer experiments.

Model Stage Name	PyTorch Operation	Output Shape	Number of Features
**natural_image**	**input**	**(3,224,224)**	**150528**
	Conv2d(3, 32, kernel_size=3, stride=2, padding=1, bias=False)	(32, 112, 112)	
	BatchNorm2d(32)	(32, 112, 112)	
	ReLU	(32, 112, 112)	
	Conv2d(32, 32, kernel_size=3, stride=1, padding=1, bias=False)	(32, 112, 112)	
	BatchNorm2d(32)	(32, 112, 112)	
	ReLU	(32, 112, 112)	

	Conv2d(32, 64, kernel_size=3, stride=1, padding=1, bias=False)	(64, 112, 112)	
	BatchNorm2d(64)	(64, 112, 112)	
	ReLU	(64, 112, 112)	
**stem**	**AvgPool2d(kernel_size=2, stride=2, padding=0)**	**(64, 56, 56)**	**200704**
**layer1**	**ResNetBlock(inplanes=64, planes=64, num_blocks=3, stride=1)**	**(256, 56, 56)**	**802816**
**layer2**	**ResNetBlock(inplanes=256, planes=128, num_blocks=4, stride=2)**	**(512, 28, 28)**	**401408**
**layer3**	**ResNetBlock(inplanes=512, planes=256, num_blocks=6, stride=2)**	**(1024, 14, 14)**	**200704**
**layer4**	**ResNetBlock(inplanes=1024, planes=512, num_blocks=3, stride=2)**	**(2048, 7, 7)**	**100352**
**attnpool**	**AttentionPool2D(spatial_dim=7, embed_dim=2048, num_heads=32, output_dim=1024)**	**(1024)**	**1024**

The ResNetBlock components of the architecture have the following structure:

1. input (x)
2. 1x1 Conv2d(inplanes, planes, stride=1, bias=False)
3. BatchNorm2d(planes)
4. ReLU
5. 3x3 Conv2d (planes, planes, stride=1, bias=False)
6. BatchNorm2d(planes)
7. ReLU
8. Downsampling after second convolution (if stride > 1): AvgPool2d(kernel_size=stride, stride=stride, padding=0)
9. 1x1 Conv2d (planes, planes * expansion, stride=1, bias=False)
10. BatchNorm2d(planes)
11. Residual connection on x (if inplanes !=planes * expansion):<br>a. (if stride>1): AvgPool2d(kernel_size=stride, stride=stride, padding=0)<br>b. 1x1 Conv2D (inplanes, planes * expansion, stride=1, bias=False)<br>c. BatchNorm2d(planes * expansion)
12. Add output from (10) to output from (11)
13. (Output) ReLU

Multiple of these residual blocks (num_blocks) are stacked together to form a single ResNetBlock. The expansion factor was set to four for all layers (expansion=4).

ResNet101

The ResNet101 architecture was proposed in [40] and has 99 convolutional layers with 1 max pooling layer and 1 average pooling layer (plus one classification layer).It has 4 residual blocks (ResNetBlocks below) which have skip (or shortcut) connections. The main difference compared to the ResNet50 model is the increased depth of the third ResNetBlock (layer3) in the model.

Model Stage Name	PyTorch Operation	Output Shape	Number of Features
**natural_image**	**input**	**(3,224,224)**	**150528**
	Conv2d(3, 64, kernel_size=7, stride=2, padding=3, bias=False)	(64, 112, 112)	

	BatchNorm2d(64)	(64, 112, 112)	
**conv1_relu1**	**ReLU**	**(64, 112, 112)**	**802816**
	MaxPool2d(kernel_size=3, stride=2, padding=1)	(64, 56, 56)	
**layer1**	**ResNetBlock(inplanes=64, planes=64, num_blocks=3, stride=1)**	**(256, 56, 56)**	**802816**
**layer2**	**ResNetBlock(inplanes=256, planes=128, num_blocks=4, stride=2)**	**(512, 28, 28)**	**401408**
**layer3**	**ResNetBlock(inplanes=512, planes=256, num_blocks=23, stride=2)**	**(1024, 14, 14)**	**200704**
**layer4**	**ResNetBlock(inplanes=1024, planes=512, num_blocks=3, stride=2)**	**(2048, 7, 7)**	**100352**
**avgpool**	**AdaptiveAvgPool2d(1,1)**	**(2048, 1, 1)**	**2048**
**final**	**Linear(2048, 1000)**	**(1000)**	**1000**

The ResNetBlock components of the architecture have the following structure (same as in ResNet50 model):

1. input (x)
2. 1x1 Conv2d(inplanes, planes, stride=1)
3. BatchNorm2d(planes)
4. ReLU
5. 3x3 Conv2d (planes, planes, stride=1)
6. BatchNorm2d(planes)
7. ReLU
8. 1x1 Conv2d (planes, planes * expansion, stride=1)
9. BatchNorm2d(planes)
10. Residual connection on x (if inplanes !=planes * expansion): 1x1 Conv2D (inplanes, planes * expansion, stride)
11. Residual connection on x (if inplanes !=planes * expansion): BatchNorm2d(planes * expansion)
12. Add output from (9) to output from (11)
13. (Output) ReLU

Multiple of these residual blocks (num_blocks) are stacked together to form a single ResNetBlock. The expansion factor was set to four for all layers (expansion=4).

AlexNet

AlexNet was proposed in [41] and consists of 5 convolutional layers, 3 max-pooling layers, and 2 fully connected layers (plus one classification layer).

Model stage names and number of features are only given for the stages used in metamer experiments.

Model Stage Name	PyTorch Operation	Output Shape	Number of Features
**natural_image**	**input**	**(3,224,224)**	**150528**
	Conv2d(3, 64, kernel_size=11, stride=4, padding=2)	(64, 55, 55)	
**relu0**	**ReLU**	**(64, 55, 55)**	**193600**
	MaxPool2d(kernel_size=3, stride=2)	(64, 27, 27)	

	Conv2d(64, 192, kernel_size=5, padding=2)	(192, 27, 27)	
**relu1**	**ReLU**	**(192, 27, 27)**	**139968**
	MaxPool2d(kernel_size=3, stride=2)	(192, 13, 13)	
	Conv2d(192, 384, kernel_size=3, padding=1)	(384, 13, 13)	
**relu2**	**ReLU**	**(384, 13, 13)**	**64896**
	Conv2d(384, 256, kernel_size=3, padding=1)	(256, 13, 13)	
**relu3**	**ReLU**	**(256, 13, 13)**	**43264**
	Conv2d(256, 256, kernel_size=3, padding=1)	(256, 13, 13)	
**relu4**	**ReLU**	**(256, 13, 13)**	**43264**
	MaxPool2d(kernel_size=3, stride=2)	(256, 6, 6)	
	Dropout(p=0.5)	(9216)	
	Linear(256 * 6 * 6, 4096)	(4096)	
**fc0_relu**	**ReLU**	**(4096)**	**4096**
	Dropout(p=0.5)	(4096)	
	Linear(4096, 4096)	(4096)	
**fc1_relu**	**ReLU**	**(4096)**	**4096**
**final**	**Linear(4096, 1000)**	**(1000)**	**1000**

ViT-B_32: CLIP

ViT-B_32 is a vision transformer architecture based on that proposed in [42]. The visual encoder from CLIP with this architecture was used for metamer generation.

Model stage names and number of features are only given for the stages used in metamer experiments.

Model Stage Name	PyTorch Operation	Output Shape	Number of Features
**natural_image**	**Input**	**(3,224,224)**	**150528**
**conv1**	**Conv2d(3, 768, kernel_size=(32,32), stride=(32,32), padding=0, bias=False)**	**(768, 7, 7)**	**37632**
	Reshape(768,49)	(768,49)	
	Permute(1,0)	(49,768)	
**class_embedding**	**Concatenate(class_embedding, x)**	**(50, 768)**	**38400**
	Add(x, positional_embedding)	(50,768)	
**ln_pre**	**LayerNorm(768, eps=1e-05, elementwise_affine=True)**	**(50,768)**	**38400**
**block_0**	**ResidualAttentionBlock(d_model=768, n_head=12, attn_mask=None)**	**(50,768)**	**38400**
	ResidualAttentionBlock(d_model=768, n_head=12, attn_mask=None)	(50,768)	
	ResidualAttentionBlock(d_model=768, n_head=12, attn_mask=None)	(50,768)	
**block_3**	**ResidualAttentionBlock(d_model=768, n_head=12, attn_mask=None)**	**(50,768)**	**38400**

	ResidualAttentionBlock(d_model=768, n_head=12, attn_mask=None)	(50,768)	
	ResidualAttentionBlock(d_model=768, n_head=12, attn_mask=None)	(50,768)	
**block_6**	**ResidualAttentionBlock(d_model=768, n_head=12, attn_mask=None)**	**(50,768)**	**38400**
	ResidualAttentionBlock(d_model=768, n_head=12, attn_mask=None)	(50,768)	
	ResidualAttentionBlock(d_model=768, n_head=12, attn_mask=None)	(50,768)	
**block_9**	**ResidualAttentionBlock(d_model=768, n_head=12, attn_mask=None)**	**(50,768)**	**38400**
	ResidualAttentionBlock(d_model=768, n_head=12, attn_mask=None)	(50,768)	
**blocks_end**	**ResidualAttentionBlock(d_model=768, n_head=12, attn_mask=None)**	**(50,768)**	**38400**
	slice(0,:)	(768)	
	LayerNorm(768, eps=1e-05, elementwise_affine=True)	(768)	
**linear_end**	**Linear(768, 512, bias=False)**	**(512)**	**512**

The transformer ResidualAttentionBlock components of the architecture have the following structure:

1. input
2. LayerNorm(d_model)
3. MultiheadAttention(d_model, n_head, attn_mask)
4. Residual: Add (1) to output of (3)
5. LayerNorm(d_model)
6. MLP Layer:<br>a. Linear (d_model, d_model * 4)<br>b. QuickGELU<br>c. Linear (d_model * 4, d_model)
7. Residual: Add output of (4) to output of (6)

Note that an attention mask of “None” as used for the visual encoder and corresponds to full attention between the tokens.

SWSL-ResNext101-32x8d

The ResxNet101-32x8d architecture was proposed in [43] and has 99 convolutional layers with 1 max pooling layer and 1 average pooling layer (plus one classification layer). It has 4 residual blocks (ResNextBlocks below) which have skip (or shortcut) connections. The main difference compared to the ResNet101 model is the presence of grouped 2D convolutions for the 3x3 convolution of the residual block.

Model Stage Name	PyTorch Operation	Output Shape	Number of Features
**natural_image**	**input**	**(3,224,224)**	**150528**
	Conv2d(3, 64, kernel_size=7, stride=2, padding=3, bias=False)	(64, 112, 112)	
	BatchNorm2d(64)	(64, 112, 112)	
**conv1_relu1**	**ReLU**	**(64, 112, 112)**	**802816**
	MaxPool2d(kernel_size=3, stride=2, padding=1)	(64, 56, 56)	
**layer1**	**ResNextBlock(inplanes=64, planes=64, num_blocks=3, stride=1, cardinality=32, base_width=8)**	**(256, 56, 56)**	**802816**
**layer2**	**ResNextBlock(inplanes=256, planes=128, num_blocks=4, stride=2, cardinality=32, base_width=8)**	**(512, 28, 28)**	**401408**

**layer3**	**ResNextBlock(inplanes=512, planes=256, num_blocks=23, stride=2, cardinality=32, base_width=8)**	**(1024, 14, 14)**	**200704**
**layer4**	**ResNextBlock(inplanes=1024, planes=512, num_blocks=3, stride=2, cardinality=32, base_width=8)**	**(2048, 7, 7)**	**100352**
**avgpool**	**AdaptiveAvgPool2d(1,1)**	**(2048, 1, 1)**	**2048**
**final**	**Linear(2048, 1000)**	**(1000)**	**1000**

The ResNextBlock components of the architecture have the following structure:

1. input (x)
2. 1x1 Conv2d(inplanes, width, stride=1, bias=False)
3. BatchNorm2d(width)
4. ReLU
5. 3x3 Conv2d (width, width, stride=1, groups=cardinality, bias=False)
6. BatchNorm2d(width)
7. ReLU
8. 1x1 Conv2d (width, planes * expansion, stride=1, bias=False)
9. BatchNorm2d(planes)
10. Residual connection on x (if inplanes !=planes * expansion): 1x1 Conv2D (inplanes, planes * expansion, stride)
11. Residual connection on x (if inplanes !=planes * expansion): BatchNorm2d(planes * expansion)
12. Add output from (9) to output from (11)
13. (Output) ReLU

Where width=(floor(planes * base_width / 64) * cardinality) and multiple of these blocks (num_blocks) are stacked together to form a single ResNextBlock. The expansion factor was set to four for all layers (expansion=4).

ViT_large_patch-16_224

ViT-large_path-16_224 is a vision transformer architecture based on that proposed in [42].

Model stage names and number of features are only given for the stages used in metamer experiments.

Model Stage Name	PyTorch Operation	Output Shape	Number of Features
**natural_image**	**Input**	**(3,224,224)**	**150528**
	Conv2d(3, 1024, kernel_size=(16,16), stride=(16,16), padding=0, bias=False)	(1024, 14, 14)	
	Reshape(768,49)	(1024,196)	
**patch_embed**	**Permute(1,0)**	**(196,1024)**	
	Concatenate(class_embedding, x)	(197,1024)	
**pos_embedding**	**Add(x, positional_embedding)**	**(197, 1024)**	
**block_0**	**TransformerBlock(dim=1024, n_head=16, qkv_bias=True)**	**(197, 1024)**	
block_1	TransformerBlock(dim=1024, n_head=16, qkv_bias=True)	(197,1024)	
block_2	TransformerBlock(dim=1024, n_head=16, qkv_bias=True)	(197, 1024)	
block_3	TransformerBlock(dim=1024, n_head=16, qkv_bias=True)	(197, 1024)	
block_4	TransformerBlock(dim=1024, n_head=16, qkv_bias=True)	(197, 1024)	

block_5	TransformerBlock(dim=1024, n_head=16, qkv_bias=True)	(197, 1024)	
**block_6**	**TransformerBlock(dim=1024, n_head=16, qkv_bias=True)**	**(197,1024)**	
block_7	TransformerBlock(dim=1024, n_head=16, qkv_bias=True)	(197, 1024)	
block_8	TransformerBlock(dim=1024, n_head=16, qkv_bias=True)	(197, 1024)	
block_9	TransformerBlock(dim=1024, n_head=16, qkv_bias=True)	(197, 1024)	
block_10	TransformerBlock(dim=1024, n_head=16, qkv_bias=True)	(197, 1024)	
block_11	TransformerBlock(dim=1024, n_head=16, qkv_bias=True)	(197, 1024)	
**block_12**	**TransformerBlock(dim=1024, n_head=16, qkv_bias=True)**	**(197,1024)**	
block_13	TransformerBlock(dim=1024, n_head=16, qkv_bias=True)	(197, 1024)	
block_14	TransformerBlock(dim=1024, n_head=16, qkv_bias=True)	(197, 1024)	
block_15	TransformerBlock(dim=1024, n_head=16, qkv_bias=True)	(197, 1024)	
block_16	TransformerBlock(dim=1024, n_head=16, qkv_bias=True)	(197, 1024)	
block_17	TransformerBlock(dim=1024, n_head=16, qkv_bias=True)	(197, 1024)	
**block_18**	**TransformerBlock(dim=1024, n_head=16, qkv_bias=True)**	**(197,1024)**	
block_19	TransformerBlock(dim=1024, n_head=16, qkv_bias=True)	(197, 1024)	
block_20	TransformerBlock(dim=1024, n_head=16, qkv_bias=True)	(197, 1024)	
block_21	TransformerBlock(dim=1024, n_head=16, qkv_bias=True)	(197, 1024)	
block_22	TransformerBlock(dim=1024, n_head=16, qkv_bias=True)	(197, 1024)	
**blocks_end**	**TransformerBlock(dim=1024, n_head=16, qkv_bias=True)**	**(197,1024)**	
	LayerNorm(768, eps=1e-05, elementwise_affine=True)	(197,1024)	
	slice(0,:)	(1024)	
**final**	**Linear(1024, 1000, bias=True)**	**(1000)**	**1000**

The TransformerBlock components of the architecture have the following structure:

1. input
2. LayerNorm(dim)
3. Attention(dim, num_heads, qkv_bias)
4. Residual: Add (1) to output of (3)
5. LayerNorm(d_model)
6. MLP Layer:<br>a. Linear (d_model, d_model * 4)<br>b. GELU<br>c. Linear (d_model * 4, d_model)
7. Residual: Add output of (4) to output of (6)

LowpassAlexNet

Modifications were made to the AlexNet architecture to reduce aliasing. The model consists of 5 convolutional layers, 5 weighted-average-pooling (HannPooling) layers, and 2 fully connected layers (plus one classification layer).

Model stage names and number of features are only given for the stages used in metamer experiments.

Model Stage Name	PyTorch Operation	Output Shape	Number of Features
**natural_image**	**input**	**(3,224,224)**	**150528**
	Conv2d(3, 64, kernel_size=11, stride=1, padding=5)	(64, 224, 224)	
	ReLU	(64, 224, 224)	
	HannPooling2d(pool_size=17, stride=4, padding=5)	(64, 55, 55)	
**relu0**	**ReLU**	**(64, 55, 55)**	**193600**
	HannPooling2d(pool_size=9, stride=2, padding=2)	(64, 27, 27)	
	Conv2d(64, 192, kernel_size=5, padding=2)	(192, 27, 27)	
**relu1**	**ReLU**	**(192, 27, 27)**	**139968**
	HannPooling2d(pool_size=9, stride=2, padding=2)	(192, 13, 13)	
	Conv2d(192, 384, kernel_size=3, padding=1)	(384, 13, 13)	
**relu2**	**ReLU**	**(384, 13, 13)**	**64896**
	Conv2d(384, 256, kernel_size=3, padding=1)	(256, 13, 13)	
**relu3**	**ReLU**	**(256, 13, 13)**	**43264**
	Conv2d(256, 256, kernel_size=3, padding=1)	(256, 13, 13)	
**relu4**	**ReLU**	**(256, 13, 13)**	**43264**
	HannPooling2d (pool_size=9, stride=2, padding=2)	(256, 6, 6)	
	Dropout(p=0.5)	(9216)	
	Linear(256 * 6 * 6, 4096)	(4096)	
**fc0_relu**	**ReLU**	**(4096)**	**4096**
	Dropout(p=0.5)	(4096)	
	Linear(4096, 4096)	(4096)	
**fc1_relu**	**ReLU**	**(4096)**	**4096**
**final**	**Linear(4096, 1000)**	**(1000)**	**1000**

VOneAlexNet

VOneAlexNet was proposed in [17] and consists of 5 convolutional layers, 3 max-pooling layers, and 2 fully connected layers (plus one classification layer). Gaussian noise rather than Poisson-like noise was used during training, as proposed in [18].

Model stage names and number of features are only given for the stages used in metamer experiments.

Model Stage Name	PyTorch Operation	Output Shape	Number of Features
**natural_image**	**input**	**(3,224,224)**	**150528**

	gabors(simple_channels=256, complex_channels=256, kernel_size=25, stride=4)	(512, 56, 56)	
**v1_output**	**additive_gaussian_noise(std=4)**	**(512, 56, 56)**	**1605632***
	Conv2d(512, 64, kernel_size=1, stride=1, bias=False)	(64, 56, 56)	
	Conv2d(64, 192, kernel_size=5, stride=2, padding=2)	(192, 28, 28)	
**relu1**	**ReLU**	**(192, 28, 28)**	**150528**
	MaxPool2d(kernel_size=3, stride=2, padding=1)	(192, 14, 14)	
	Conv2d(192, 384, kernel_size=3, padding=1)	(384, 14, 14)	
**relu2**	**ReLU**	**(384, 14, 14)**	**75264**
	Conv2d(384, 256, kernel_size=3, padding=1)	(256, 14, 14)	
**relu3**	**ReLU**	**(256, 14, 14)**	**50176**
	Conv2d(256, 256, kernel_size=3, padding=1)	(256, 14, 14)	
**relu4**	**ReLU**	**(256, 14, 14)**	**50176**
	MaxPool2d(kernel_size=3, stride=2, padding=1)	(256, 7, 7)	
	Dropout(p=0.5)	(12544)	
	Linear(256 * 6 * 6, 4096)	(4096)	
**fc0_relu**	**ReLU**	**(4096)**	**4096**
	Dropout(p=0.5)	(4096)	
	Linear(4096, 4096)	(4096)	
**fc1_relu**	**ReLU**	**(4096)**	**4096**
**final**	**Linear(4096, 1000)**	**(1000)**	**1000**

*Metamers were generated from the output of the VOneNet block. We note that this model stage has more parameters than the comparison relu0 stage of AlexNet and LowpassAlexNet, but that we plot the relu0/VOneBlock on the same line in Figure 6d and Supplementary Figure 13. Subsequent stages (relu1 onwards) have similar dimensionality, although differ slightly due to the padding in the VOneAlexNet architecture.

Auditory Models

CochResNet50

The CochResNet50 model is a ResNet50 backbone architecture applied to a cochleagram representation (such that 2D convolutions learned on the cochleagram).

Model Stage Name	PyTorch Operation	Output Shape	Number of Features
**natural_audio**	**Input (waveform)**	**(40000)**	**40000**
**cochleagram**	**Cochleagram**	**(1,211,390)**	**82290**
	Conv2d(1, 64, kernel_size=7, stride=2, padding=3, bias=False)	(64, 106, 195)	
	BatchNorm2d(64)	(64, 106, 195)	

**conv1_relu1**	**ReLU**	**(64, 106, 195)**	**1322880**
	MaxPool2d(kernel_size=3, stride=2, padding=1)	(64, 53, 98)	
**layer1**	**ResNetBlock(inplanes=64, planes=64, num_blocks=3, stride=1)**	**(256, 53, 98)**	**1329664**
**layer2**	**ResNetBlock(inplanes=256, planes=128, num_blocks=4, stride=2)**	**(512, 27, 49)**	**677376**
**layer3**	**ResNetBlock(inplanes=512, planes=256, num_blocks=6, stride=2)**	**(1024, 14, 25)**	**358400**
**layer4**	**ResNetBlock(inplanes=1024, planes=512, num_blocks=3, stride=2)**	**(2048, 7, 13)**	**186368**
**avgpool**	**AdaptiveAvgPool2d(1,1)**	**(2048, 1, 1)**	**2048**
**final**	**Linear(2048, 794)**	**(794)**	**794**

The ResNetBlock components of the architecture have the following structure (same as in ResNet50 visual model):

1. input (x)
2. 1x1 Conv2d(inplanes, planes, stride=1)
3. BatchNorm2d(planes)
4. ReLU
5. 3x3 Conv2d (planes, planes, stride=1)
6. BatchNorm2d(planes)
7. ReLU
8. 1x1 Conv2d (planes, planes * expansion, stride=1)
9. BatchNorm2d(planes)
10. Residual connection on x (if inplanes !=planes * expansion): 1x1 Conv2D (inplanes, planes * expansion, stride)
11. Residual connection on x (if inplanes !=planes * expansion): BatchNorm2d(planes * expansion)
12. Add output from (9) to output from (11)
13. (Output) ReLU

Multiple of these residual blocks (num_blocks) are stacked together to form a single ResNetBlock. The expansion factor was set to four for all layers (expansion=4).

CochCNN9

The CochCNN9 architecture is based on found in [36] through a neural network architecture search. The architecture differs in that the input to the first stage of the model is not reshaped to 256x256, rather it is maintained as the 211x390 size cochleagram. The convolutional layer filters and pooling regions are similarly reshaped to maintain the approximate receptive field size in frequency and time. The model is also trained with batch normalization rather than the local response normalization used in [36].

Model Stage Name	PyTorch Operation	Output Shape	Number of Features
**natural_audio**	**Input (waveform)**	**(40000)**	**40000**
cochleagram	Cochleagram	(1,211,390)	**82290**
	BatchNorm2d(1)	(1,211,390)	
	Conv2d(1, 96, kernel_size=[7, 14], stride=[3, 3], padding='same')	(96, 71, 130)	

**relu0**	**ReLU**	**(96, 71, 130)**	**886080**
	MaxPool2d(kernel_size=[2,5] , stride=[2,2], padding='same')	(96, 36, 65)	
	BatchNorm2d(96)	(96, 36, 65)	
	Conv2d(96, 256, kernel_size=[4,8], stride=[2,2], padding='same')	(256, 18, 33)	
**relu1**	**ReLU**	**(256, 18, 33)**	**152064**
	MaxPool2d(kernel_size=[2,5] , stride=[2,2], padding='same')	(256, 9, 17)	
	BatchNorm2d(256)	(256, 9, 17)	
	Conv2d(256, 512, kernel_size=[2,5], stride=[1,1], padding='same')	(512, 9, 17)	
**relu2**	**ReLU**	**(512, 9, 17)**	**78336**
	Conv2d(512, 1024, kernel_size=[2,5], stride=[1,1], padding='same')	(1024, 9, 17)	
**relu3**	**ReLU**	**(1024, 9, 17)**	**156672**
	Conv2d(1024, 512, kernel_size=[2,5], stride=[1,1], padding='same')	(512, 9, 17)	
**relu4**	**ReLU**	**(512, 9, 17)**	**78336**
**avgpool**	**AvgPool(kernel_size=[2,5] , stride=[2,2], padding='same')**	**(512, 5, 9)**	**23040**
	Linear(512*9*5 , 4096)	(4096)	
**relufc**	**ReLU**	**(4096)**	**4096**
	Dropout(p=0.5)	(4096)	
**final**	**Linear(4096, 794)**	**(794)**	**794**

Supplementary Information References

1. Feather, J., Durango, A., Gonzalez, R. & McDermott, J. Metamers of neural networks reveal divergence from human perceptual systems. in *Advances in Neural Information Processing Systems* (2019).

2. Abadi, M. *et al.* TensorFlow: A system for large-scale machine learning. *arXiv [cs.DC]* (2016).

3. Kingma, D. P. & Ba, J. Adam: A Method for Stochastic Optimization. *arXiv [cs.LG]* (2014).

4. Geirhos, R., Temme, C. R. M. & Rauber, J. Generalisation in humans and deep neural networks. *Adv. Neural Inf. Process. Syst.* (2018).

5. Tsipras, D., Santurkar, S., Engstrom, L., Turner, A. & Madry, A. Robustness may be at odds with accuracy. *arXiv [stat.ML]* (2018).

6. Paszke, A. *et al.* PyTorch: An Imperative Style, High-Performance Deep Learning Library. in *Advances in Neural Information Processing Systems 32* (eds. Wallach, H. et al.) 8024–8035 (Curran Associates, Inc., 2019).

7. Engstrom, L., Ilyas, A., Salman, H., Santurkar, S. & Tsipras, D. Robustness (Python Library). Preprint at https://github.com/MadryLab/robustness (2019).

8. Deng, J. *et al.* ImageNet: A large-scale hierarchical image database. in *2009 IEEE Conference on Computer Vision and Pattern Recognition* (IEEE, 2009). doi:10.1109/cvpr.2009.5206848.

9. Radford, A. *et al.* Learning Transferable Visual Models From Natural Language Supervision. in *Proceedings of the 38th International Conference on Machine Learning* (eds. Meila, M. & Zhang, T.) vol. 139 8748–8763 (PMLR, 18--24 Jul 2021).

10. Yalniz, I. Z., Jégou, H., Chen, K., Paluri, M. & Mahajan, D. Billion-scale semi-supervised learning for image classification. *arXiv [cs.CV]* Preprint at http://arxiv.org/abs/1905.00546 (2019).

11. Steiner, A. P. *et al.* How to train your ViT? Data, Augmentation, and Regularization in Vision Transformers. *Transactions on Machine Learning Research* (2022).

12. Konkle, T. & Alvarez, G. A. A self-supervised domain-general learning framework for human ventral stream representation. *Nat. Commun.* **13**, 491 (2022).

13. Smith, L. N. Cyclical Learning Rates for Training Neural Networks. in *2017 IEEE Winter Conference on Applications of Computer Vision (WACV)* 464–472 (2017).

14. Geirhos, R. *et al.* ImageNet-trained CNNs are biased towards texture; increasing shape bias improves accuracy and robustness. in *International Conference on Learning Representations* (2019).

15. Huang, X. & Belongie, S. Arbitrary style transfer in real-time with adaptive instance normalization. in *2017 IEEE International Conference on Computer Vision (ICCV)* (IEEE, 2017). doi:10.1109/iccv.2017.167.

16. Serre, T., Oliva, A. & Poggio, T. A feedforward architecture accounts for rapid categorization. *Proc. Natl. Acad. Sci. U. S. A.* **104**, 6424–6429 (2007).

17. Dapello, J. *et al.* Simulating a primary visual cortex at the front of CNNs improves robustness to image perturbations. *Adv. Neural Inf. Process. Syst.* **33**, 13073–13087 (2020).

18. Dapello, J. *et al.* Neural population geometry reveals the role of stochasticity in robust perception. *Adv. Neural Inf. Process. Syst.* **34**, (2021).

19. Croce, F. *et al.* RobustBench: a standardized adversarial robustness benchmark. in *Thirty-fifth Conference on Neural Information Processing Systems Datasets and Benchmarks Track* (2021).

20. Debenedetti, E., Sehwag, V. & Mittal, P. A Light Recipe to Train Robust Vision Transformers. Preprint at https://doi.org/10.48550/ARXIV.2209.07399 (2022).

21. Salman, H., Ilyas, A., Engstrom, L., Kapoor, A. & Madry, A. Do adversarially robust imagenet models transfer better? *Adv. Neural Inf. Process. Syst.* **33**, 3533–3545 (2020).

22. Wong, E., Rice, L. & Kolter, J. Z. Fast is better than free: Revisiting adversarial training. in *International Conference on Learning Representations* (2020).

23. Nguyen, A., Yosinski, J. & Clune, J. Deep neural networks are easily fooled: High confidence predictions for unrecognizable images. in *2015 IEEE Conference on Computer Vision and Pattern Recognition (CVPR)* (IEEE, 2015). doi:10.1109/cvpr.2015.7298640.

24. Sabour, S., Cao, Y., Faghri, F. & Fleet, D. J. Adversarial Manipulation of Deep Representations. in *4th International Conference on Learning Representations, ICLR 2016, San Juan, Puerto Rico, May 2-4, 2016, Conference Track Proceedings* (eds. Bengio, Y. & LeCun, Y.) (2016).

25. Schrimpf, M. *et al.* Integrative benchmarking to advance neurally mechanistic models of human intelligence. *Neuron* **108**, 413–423 (2020).

26. Hendrycks, D. & Dietterich, T. Benchmarking Neural Network Robustness to Common Corruptions and Perturbations. in *International Conference on Learning Representations* (2018).

27. Geirhos, R. *et al.* Partial success in closing the gap between human and machine vision. in *Advances in Neural Information Processing Systems* (2021).

28. Paul, D. B. & Baker, J. M. The design for the wall street journal-based CSR corpus. in *Proceedings of the workshop on Speech and Natural Language - HLT '91* (Association for Computational Linguistics, 1992). doi:10.3115/1075527.1075614.

29. Köhn, A., Stegen, F. & Baumann, T. Mining the Spoken Wikipedia for Speech Data and Beyond. in *Proceedings of the Tenth International Conference on Language Resources and Evaluation (LREC 2016)* (eds. Chair), N. C. (conference et al.) (European Language Resources Association (ELRA), 2016).

30. Zue, V. W. & Seneff, S. Transcription and alignment of the TIMIT database. in *Recent Research Towards Advanced Man-Machine Interface Through Spoken Language* 515–525 (Elsevier, 1996).

31. Gemmeke, J. F. *et al.* Audio Set: An ontology and human-labeled dataset for audio events. in *2017 IEEE International Conference on Acoustics, Speech and Signal Processing (ICASSP)* (IEEE, 2017). doi:10.1109/icassp.2017.7952261.

32. McDermott, J. H. & Simoncelli, E. P. Sound texture perception via statistics of the auditory periphery: evidence from sound synthesis. *Neuron* **71**, 926–940 (2011).

33. Glasberg, B. R. & Moore, B. C. J. Derivation of auditory filter shapes from notched-noise data. *Hear. Res.* **47**, 103–138 (1990).

34. Chi, T., Ru, P. & Shamma, S. A. Multiresolution spectrotemporal analysis of complex sounds. *J. Acoust. Soc. Am.* **118**, 887–906 (2005).

35. Santoro, R. *et al.* Encoding of natural sounds at multiple spectral and temporal resolutions in the human auditory cortex. *PLoS Comput. Biol.* **10**, e1003412 (2014).

36. Kell, A. J. E., Yamins, D. L. K., Shook, E. N., Norman-Haignere, S. V. & McDermott, J. H. A task-optimized neural network replicates human auditory behavior, predicts brain responses, and reveals a cortical processing hierarchy. *Neuron* **98**, 630-644.e16 (2018).

37. Norman-Haignere, S., Kanwisher, N. G. & McDermott, J. H. Distinct cortical pathways for music and speech revealed by hypothesis-free voxel decomposition. *Neuron* **88**, 1281–1296 (2015).

38. Kubilius, J. *et al.* Brain-Like Object Recognition with High-Performing Shallow Recurrent ANNs. in *Advances in Neural Information Processing Systems* vol. 32 (Curran Associates, Inc., 2019).

39. Simonyan, K. & Zisserman, A. Very Deep Convolutional Networks for Large-Scale Image Recognition. *arXiv [cs.CV]* (2014).

40. He, K., Zhang, X., Ren, S. & Sun, J. Identity mappings in deep residual networks. *arXiv [cs.CV]* (2016).

41. Krizhevsky, A., Sutskever, I. & Hinton, G. E. ImageNet classification with deep convolutional neural networks. in *Advances in neural information processing systems* vol. 25 1097–1105 (2012).

42. Dosovitskiy, A. *et al.* An Image is Worth 16x16 Words: Transformers for Image Recognition at Scale. in *International Conference on Learning Representations* (2021).

43. Xie, S., Girshick, R., Dollar, P., Tu, Z. & He, K. Aggregated residual transformations for deep neural networks. in *2017 IEEE Conference on Computer Vision and Pattern Recognition (CVPR)* (IEEE, 2017). doi:10.1109/cvpr.2017.634.
