# Supplementary material for: Model metamers reveal divergent invariances between biological and artificial neural networks
Source: Nat Neurosci. 2023 Oct 16;26(11):2017–34. doi: 10.1038/s41593-023-01442-0 (PMC10620097; doi:10.1038/s41593-023-01442-0)
Supplement: Supplementary file 2 — Reporting Summary [file 41593_2023_1442_MOESM2_ESM.pdf]

nature portfolio

Corresponding author(s): Jenelle Feather and Josh McDermott

Last updated by author(s): Aug 25, 2023

Reporting Summary

Nature Portfolio wishes to improve the reproducibility of the work that we publish. This form provides structure for consistency and transparency in reporting. For further information on Nature Portfolio policies, see our Editorial Policies and the Editorial Policy Checklist.

Statistics

For all statistical analyses, confirm that the following items are present in the figure legend, table legend, main text, or Methods section.

n/a	Confirmed	
☐	☒	The exact sample size (*n*) for each experimental group/condition, given as a discrete number and unit of measurement
☐	☒	A statement on whether measurements were taken from distinct samples or whether the same sample was measured repeatedly
☐	☒	The statistical test(s) used AND whether they are one- or two-sided<br>*Only common tests should be described solely by name; describe more complex techniques in the Methods section.*
☒	☐	A description of all covariates tested
☐	☒	A description of any assumptions or corrections, such as tests of normality and adjustment for multiple comparisons
☐	☒	A full description of the statistical parameters including central tendency (e.g. means) or other basic estimates (e.g. regression coefficient) AND variation (e.g. standard deviation) or associated estimates of uncertainty (e.g. confidence intervals)
☐	☒	For null hypothesis testing, the test statistic (e.g. *F*, *t*, *r*) with confidence intervals, effect sizes, degrees of freedom and *P* value noted<br>*Give P values as exact values whenever suitable.*
☒	☐	For Bayesian analysis, information on the choice of priors and Markov chain Monte Carlo settings
☒	☐	For hierarchical and complex designs, identification of the appropriate level for tests and full reporting of outcomes
☐	☒	Estimates of effect sizes (e.g. Cohen's *d*, Pearson's *r*), indicating how they were calculated

Our web collection on statistics for biologists contains articles on many of the points above.

Software and code

Policy information about availability of computer code

Data collection	Amazon Mechanical Turk was used to collect the online human behavioral data with custom experiment code, provided at https://github.com/jenellefeather/model_metamers_pytorch
Data analysis	For data analysis and model training we used a Python 3.8.2 conda environment, including PyTorch 1.5.0 (details of full conda environment are provided at https://github.com/jenellefeather/model_metamers_pytorch), and when models or GPU hardware required operations above PyTorch 1.5.0 we used a conda environment with PyTorch 1.12.1. Power analysis was performed with G*Power 3. Voxelwise encoding analysis used RidgeCV from the scikit learn library version 0.23.1. ANOVAs were performed with MATLAB 2021a.

For manuscripts utilizing custom algorithms or software that are central to the research but not yet described in published literature, software must be made available to editors and reviewers. We strongly encourage code deposition in a community repository (e.g. GitHub). See the Nature Portfolio guidelines for submitting code & software for further information.

Data

Policy information about availability of data

All manuscripts must include a data availability statement. This statement should provide the following information, where applicable:

- Accession codes, unique identifiers, or web links for publicly available datasets
- A description of any restrictions on data availability
- For clinical datasets or third party data, please ensure that the statement adheres to our policy

Human data (auditory and visual human recognition performance and summarized fMRI data), and trained model checkpoints are available at https://github.com/jenellefeather/model_metamers_pytorch. The same repository also includes an interface to view/listen to the generated metamers used in the human recognition experiments. The Word-Speaker-Noise training dataset is available from the authors upon request.

Human research participants

Policy information about studies involving human research participants and Sex and Gender in Research.

Reporting on sex and gender	Gender was self-reported by study participants. A gender-based analysis was not performed as we were interested in the comparison between humans and computational models, and did not investigate individual differences in human behavior.
Population characteristics	See "Behavioural & social sciences study design".
Recruitment	Online participants were recruited on Amazon's Mechanical Turk platform, using a geographic filter to restrict participation to individuals with IP addresses in the United States or Canada. During recruitment, participants were told that they would "Listen to audio clips and report the word that is heard" (auditory experiment) or "Choose a category for a presented image from 16 categories" (visual experiment). In principle, the fact that participants were self-selected could have induced biases in overall performance levels.
Ethics oversight	The study was approved by the Committee on the Use of Humans as Experimental Subjects at MIT.

Note that full information on the approval of the study protocol must also be provided in the manuscript.

Field-specific reporting

Please select the one below that is the best fit for your research. If you are not sure, read the appropriate sections before making your selection.

☐ Life sciences ☒ Behavioural & social sciences ☐ Ecological, evolutionary & environmental sciences

For a reference copy of the document with all sections, see nature.com/documents/nr-reporting-summary-flat.pdf

Behavioural & social sciences study design

All studies must disclose on these points even when the disclosure is negative.

Study description	This quantitative study examines whether humans can recognize synthetic auditory and visual stimuli. We tested participants from Canada and the U.S.A. recruited online. Human data was compared to computational models, and all human behavioral comparisons were within-participant.
Research sample	Online participants were used for convenience. We did not screen for age or self-reported gender. Based on our previous experience running online experiments, this sample was representative of typical online participant cohorts.<br><br>Visual Behavioral Experiments:<br>A total of 417 participants completed all or part of the online visual study. Of these, 182 self-identified as female, 227 as male, and 8 did not report; mean age=40.9, minimum age=20, maximum age=78. Of the 270 participants that passed the full screening criteria, 125 self-identified as female, 143 as male, and 2 did not report; mean age=42.1, minimum age=20, maximum age=78.<br><br>Auditory Behavioral Experiments:<br>A total of 377 participants completed all or part of the online auditory study. Of these, 149 self-identified as female, 201 as male, 4 identified as non-binary, and 23 did not report; mean age=38.7, minimum age=3, maximum age=77. Of the 120 participants that passed the full screening criteria, all self-reported normal hearing, and 45 self-identified as female, 68 as male, and 7 chose not to report; mean age=39.0, minimum age=22, maximum age=77.
Sampling strategy	We used convenience sampling. Online participants were recruited on Amazon's Mechanical Turk platform, using a geographic filter to restrict participation to individuals with IP addresses in the United States or Canada.<br><br>The number of participants was determined based on a pilot experiment with 10 participants and a power analysis for the smallest

anticipated effect, as this estimated an upper bound on the sample sizes that would be needed across experiments. This power analysis resulted in a target sample size of 20 participants for each of the vision and auditory experiments.

Batches of Amazon Turk HITs were posted until we met the 20 participant criteria required for each experiment. Due to the nature of amazon turk and the number of participants who would fail the exclusion criteria, we occasionally had more participants pass than the 20 participants required. We kept all of this data and noted the "N" for each experiment.

Data collection

Online participants were recruited through Amazon's Mechanical Turk platform, using a geographic filter to restrict participation to individuals logging on from the United States or Canada. Participants completed the study at their own computer and entered their own data. For audio experiments, participants completed a brief "headphone-check" experiment intended to help ensure that they were wearing headphones or earphones. Blinding was not applicable to this study as the analysis was automated.

Timing

Online experiments were conducted between June 2021 and December 2022.

Data exclusions

To increase data quality for behavioral data collected on Amazon Mechanical Turk we pre-estabilished exclusion criteria based on in-lab and online pilot experiments.

Vision Behavioral Experiments:
Before the main experiment, participants completed a 12-trial demo experiment which was used as a screen to remove participants who were distracted, misunderstood the task instructions, had browser incompatibilities, or were otherwise unable to complete the task. Participants were only allowed to start the main experiment if they correctly answered 7/12 correct on the demo experiment, which was the minimum that were correctly answered for these same demo stimuli by 16 in-lab participants in a pilot experiment. 341/417 participants passed the demo experiment and chose to move onto the main experiment.

Within each main vision experiment, participants completed 16 catch trials. These catch trials each consisted of an image that exactly matched the icon for one of the classes. Participant data was only included in the analysis if the participant got 15/16 of these catch trials correct (270/341 participants were included).

Auditory Behavioral Experiments:
As with the vision experiments, before the main experiment, participants completed a demo experiment of 12 trials without feedback which was used to screen out poorly performing participants. A screening criteria was set at 5/12, which was the minimum for 16 in-lab participants in earlier work. 154/224 participants passed the demo experiment and chose to move onto the main experiment.

Participants completed 16 catch trials within the main experiment, consisting of a single word corresponding to one of the classes. Participant data was only included in the analysis if the participant got 15/16 of these trials correct (this inclusion criterion removed 8/154 participants). As the audio experiment was long in duration, some participants chose to leave the experiment early and their data was excluded from analysis (23/154). An additional 3 participants were excluded due to self-reported hearing loss.

Non-participation

23 participants chose to leave the auditory study early and their data was excluded from the analysis. Participants could choose to leave the study early for any reason, and were not required to tell us why.

Randomization

There were a total of 12 vision experiments and 6 audio experiments, and each participant could only complete one experiment of each type (we used Amazon Mechanical Turk qualifications to prevent participants from repeating an experiment). Only one experiment of each type was posted at a time, and the group assignment was convenience based relying on the worker pool at at that time.

Gender, age, and other participant demographics were only tabulated after all experimental data was collected and were not used for the experiment assignment.

Reporting for specific materials, systems and methods

We require information from authors about some types of materials, experimental systems and methods used in many studies. Here, indicate whether each material, system or method listed is relevant to your study. If you are not sure if a list item applies to your research, read the appropriate section before selecting a response.

Materials & experimental systems

n/a	Involved in the study
☒	☐ Antibodies
☒	☐ Eukaryotic cell lines
☒	☐ Palaeontology and archaeology
☒	☐ Animals and other organisms
☒	☐ Clinical data
☒	☐ Dual use research of concern

Methods

n/a	Involved in the study
☒	☐ ChIP-seq
☒	☐ Flow cytometry
☐	☒ MRI-based neuroimaging

Magnetic resonance imaging

Experimental design

Design type	Block Design
Design specifications	Data was first published in Norman-Haignere et al. 2015, and was re-analyzed for this paper. The sounds were presented in a block design with five presentations of each two-second sound. To prevent sounds from being played at the same time as scanner noise, a single fMRI volume was collected following each sound presentation ("sparse scanning"). This resulted in a 17-second block. Blocks were grouped into 11 runs with 15 stimuli each and four blocks of silence. Silence blocks were the same duration as the stimulus blocks and were used to estimate the baseline response.
Behavioral performance measures	Participants performed a sound-intensity discrimination task to motivate them to attend to the sounds. One sound in the block of five was presented 7dB lower than the other four (the quieter sound was never the first sound) and participants were instructed to press a button when they heard the quieter sound. Sounds were presented with MR-compatible earphones (Sensimetrics S14) at 75 dB SPL for the louder sounds and 68dB SPL for the quieter sounds.

Acquisition

Imaging type(s)	Functional
Field strength	MR data was collected on a 3T Siemens Trio scanner with a 32-channel head coil at the Athinoula A. Martinos Imaging Center of the McGovern Institute for Brain Research at MIT.
Sequence & imaging parameters	Repetition time (TR) was 3.4 s (acquisition time was only 1 s due to sparse scanning), echo time (TE) was 30 ms, and flip angle was 90 degrees. For each run, the five initial volumes were discarded to allow homogenization of the magnetic field. In-plane resolution was 2.1 x 2.1 mm (96 x 96 matrix), and slice thickness was 4 mm with a 10% gap, yielding a voxel size of 2.1 x 2.1 x 4.4 mm. iPAT was used to minimize acquisition time. T1-weighted anatomical images were collected in each subject (1mm isotropic voxels) for alignment and surface reconstruction.
Area of acquisition	Each functional volume consisted of fifteen slices oriented parallel to the superior temporal plane, covering the portion of the temporal lobe superior to and including the superior temporal sulcus.

Diffusion MRI ☐ Used ☒ Not used

Preprocessing

Preprocessing software	fMRI preprocessing followed that published in Norman-Haignere et al. 2015. Functional volumes were preprocessed using FSL and in-house MATLAB scripts. Volumes were skull-stripped, and voxel time courses were linearly detrended. Each run was aligned to the anatomical volume using FLIRT and BBRegister. These preprocessed functional volumes were then resampled to vertices on the reconstructed cortical surface computed via FreeSurfer, and were smoothed on the surface with a 3mm FWHM 2D Gaussian kernel to improve SNR. All analyses were done in this surface space, but for ease of discussion we refer to vertices as ''voxels'' in this paper. For each of the three scan sessions, the mean response of each voxel (in the surface space) to each stimulus block was estimated by averaging the response of the second through the fifth acquisitions after the onset of each block (the first acquisition was excluded to account for the hemodynamic lag). These signal-averaged responses were converted to percent signal change (PSC) by subtracting and dividing by each voxel's response to the blocks of silence. These PSC values were then downsampled from the surface space to a 2mm isotropic grid on the FreeSurfer-flattened cortical sheet.
Normalization	Data were not aligned to a standard space, as analysis was performed within localized voxels in each participant.
Normalization template	Data was not normalized.
Noise and artifact removal	Volumes were corrected for motion and slice time.
Volume censoring	None

Statistical modeling & inference

Model type and settings	We performed two analyses of the fMRI data. (1) An encoding model mapping neural network activations to the fMRI responses and (2) an RSA analysis between the neural network activations and the fMRI data.
Effect(s) tested	For each ROI, we test for a Spearman rank-ordered correlation between model metamer recognizability and the (1) variance in the voxel responses explained by the encoding model (2) the Spearman correlation between the fMRI representational dissimilarity matrix and the model representational dissimilarity matrix.

Specify type of analysis: ☐ Whole brain ☒ ROI-based ☐ Both

Anatomical location(s): We used functional ROIs, identified and tested using independent data.

Statistic type for inference (See Eklund et al. 2016)	Encoding analysis was performed voxel-wise. RSA analysis was performed cluster-wise within each ROI.

Correction

We test 5 ROIs (all auditory, tonotopic, pitch, speech, and music). For the evaluated Spearman correlations, we Bonferroni-corrected the p-values for the correlation between model metamer recognizability and variance explained by multiplying the p-value by 5 (the number of tests performed).

Models & analysis

n/a	Involved in the study
☒	☐ Functional and/or effective connectivity
☒	☐ Graph analysis
☐	☒ Multivariate modeling or predictive analysis

Multivariate modeling and predictive analysis

Features were extracted from deep neural network model stages and a regularized linear regression model was fit between the model activations and each voxel's response, fitting the model using 83/165 sounds and testing the predictions on the remaining 82 sounds. Full details of the modeling procedure are included in the Methods section.
